# Asymmetric ring contraction of 2-hydroxypyranones by borrowing hydrogen biocatalysis

**DOI:** 10.1039/d5sc02591e

**Published:** 2025-08-29

**Authors:** Yuchang Liu, Adam O'Connell, J. D. Rolfes, Jan Deska

**Affiliations:** a Department of Chemistry, University of Helsinki A.I. Virtasen aukio 1 00560 Helsinki Finland liuych@tib.cas.cn jan.deska@helsinki.fi; b National Engineering Laboratory for Industrial Enzymes, Tianjin Engineering Research Center of Biocatalytic Technology, National Center of Technology Innovation for Synthetic Biology PR China; c Tianjin Institute of Industrial Biotechnology, Chinese Academy of Sciences Tianjin 300308 PR China; d Albert Hofmann Institute for Physiochemical Sustainability Albert-Schweitzer-Str. 22 32602 Vlotho Germany

## Abstract

Ring contraction reactions facilitate easy access to carbo- and heterocyclic scaffolds from readily available precursors and have therefore enjoyed great popularity as a strategy in organic synthesis for a long time. By repurposing commercial alcohol dehydrogenases as borrowing hydrogen biocatalysts, we were able to develop a rare example of an enzymatic ring contraction methodology, where racemic 2-hydroxypyranones can be converted in an enantioconvergent manner to the corresponding 5-membered butenolides. The overall transformation is redox self-sufficient without the need for cofactor recycling systems and delivers γ-lactones in excellent optical purities. To underline the synthetic value of this ring contraction, the methodology was successfully applied in the preparation of an *Osmunda butenolide* and in the formal total synthesis of *threo*-cavernosine. Moreover, the biocatalytic tool was incorporated into a multi-step cascade consisting of six enzymes, achieving the formal enantioselective dearomatization of a furfuryl alcohol to deliver the corresponding saturated γ-lactone in >99% *ee*.

## Abstract 2

Alcohol dehydrogenases catalyze a ring contraction reaction to yield butenolides in high optical purity. The borrowing hydrogen process is applied in the total synthesis of cavernosine and in a multi-enzyme cascade for the valorization of furans.

## Graphical abstract

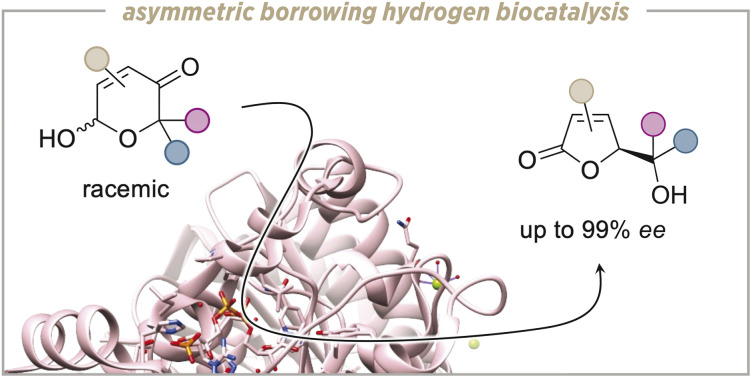

## Introduction

The skeletal rearrangement of existing carbo- or heterocyclic scaffolds to create enlarged or contracted cyclic architectures has for a long time been a logical consideration to address challenging ring structures in synthetic-organic chemistry. These ring contraction and ring expansion reactions can offer attractive alternatives in retrosynthetic analysis and planning, in particular when cyclization reactions are hampered by for example unfavorable ring sizes.^1–4^ Furthermore, the contraction/extension approach allows for the easy conversion of readily available or abundant cyclic precursors to yield more complex, value-added products.^5–7^ Traditional ring contraction reactions often proceed through the migration of bonds to electron-deficient intermediates, including Wagner–Meerwein,^8,9^ (semi)pinacol,^10^ and Wolff rearrangements,^11^ or through skeletal realignment of anionic intermediates such as in Favorskii,^12,13^ and benzilic acid rearrangements.^14^ Other types of ring contractions operate through extrusion of small molecules as exemplified in Ramberg–Bäcklund olefinations (loss of SO_2_),^15–18^ the decomposition of diazenes (loss of N_2_),^19–22^ or photochemical decarbonylations (loss of CO).^23–26^ But ring contraction reactions are not entirely limited to the synthetic chemistry world, and also nature does occasionally take advantage of similar transformations in the biosynthesis of complex metabolites. Examples of these natural ring contractions include the biosynthesis of rare sugars such as the rearrangement of activated pyranoses to UDP-*d*-apiose,^27^ the generation of the corrin core of vitamin B_12_ through the natural contractase CbiH_60_,^28^ the conversion of a benzodiazepinedione to the quinolone alkaloid viridicatin,^29^ or the xanthone contractions to cyclopentadienols such as coniothyrione,^30^ and the remisporines.^31,32^ On the other hand, synthetic biotransformations addressing ring contractions *in vitro* have so far remained scarce at best.^33–35^

**Scheme 1:**
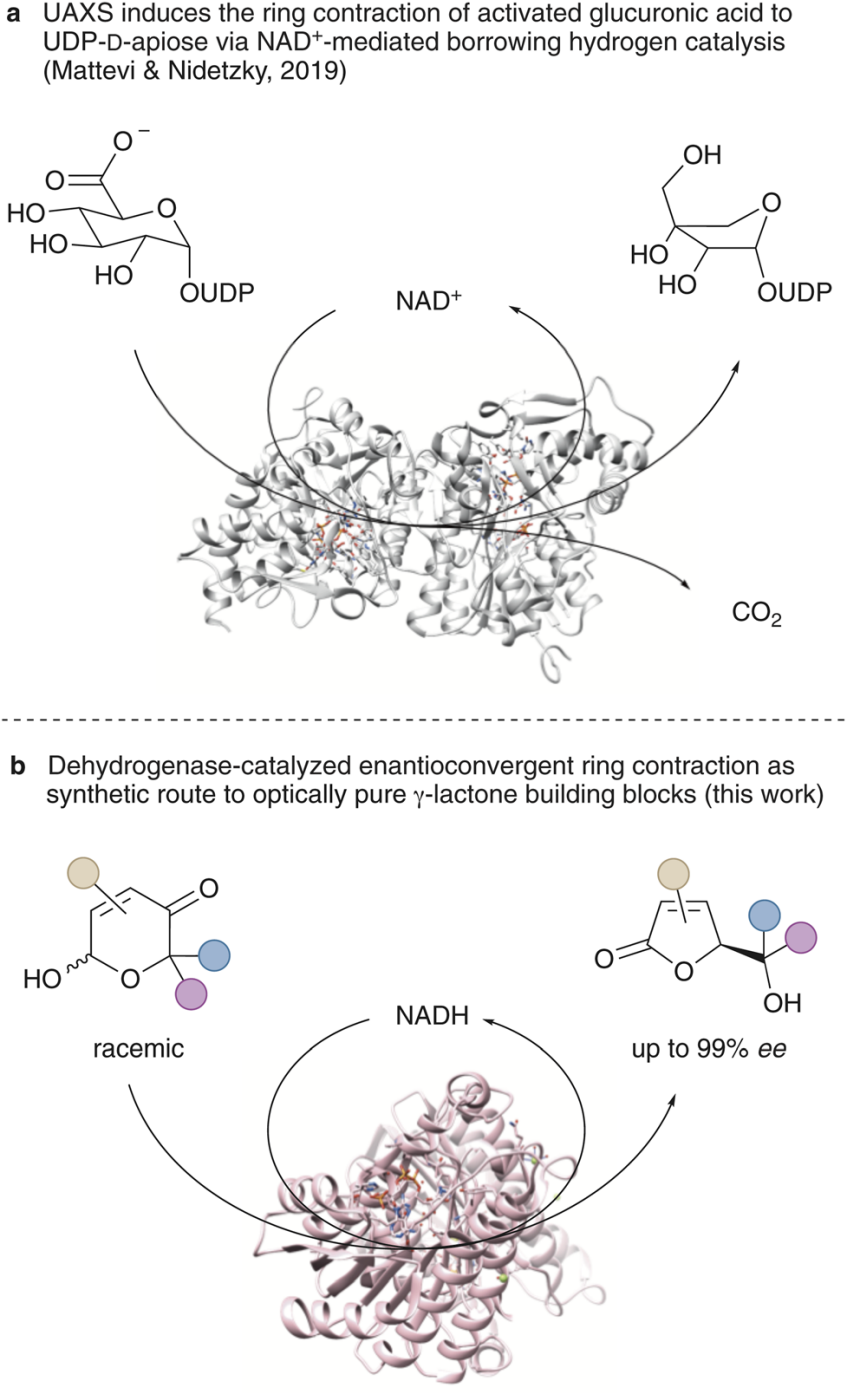
Redox-neutral enzymatic ring contractions based on the borrowing hydrogen principle: (a) *in vivo* production of rare carbohydrates; (b) general biocatalysis tool for the preparation of enantioenriched γ-butenolides.

While our recent research has focused to a significant extent on the (stereo)selective and biocatalytic synthesis of five-membered oxygen heterocycles,^36–43^ the herein described methodology exploiting an enzymatic ring contraction for the production of γ-butenolides has developed purely serendipitously and as a result of an unanticipated and undesired side reaction. Even though certain similarities to the UDP-*d*-apiose biosynthesis (*via* UAXS) deciphered by Mattevi and Nidetzky are apparent (Scheme 1a) – specifically the redox self-sufficient nature of the UAXS applying a nicotinamide cofactor to mediate a natural borrowing hydrogen catalysis cycle – the focus of the title reaction is on synthetic utility to deliver γ-lactone building blocks in high optical purity. This unprecedented biocatalytic synthesis tool based on readily available dehydrogenases exploits a much more rare inverse borrowing hydrogen strategy in which the reduced cofactor acts as H-donor and the substrate undergoes a reduction–oxidation sequence (Scheme 1b). The study covers a detailed account on the underlying pyranone chemistry, an in-depth discussion on substrate scope and limitations as well as the incorporation of the new contractase module into a complex multi-enzyme *in vitro* cascade.

## Results & discussion

The ring contraction of pyranones: from an undesired side reactivity to the blueprint for a new catalysis pathway.

**Scheme 2:**
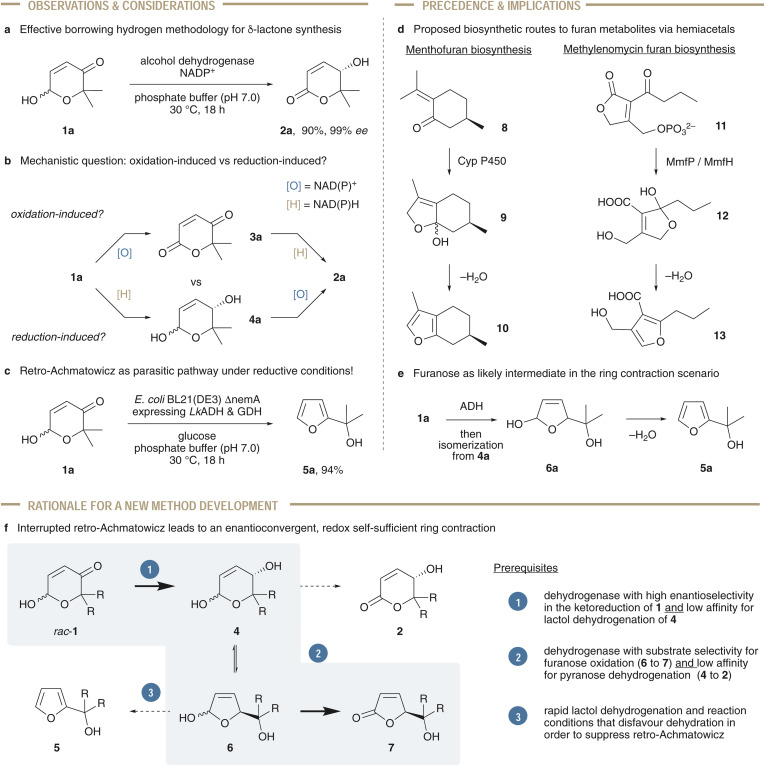
Development of a blueprint for the investigation of an enantioselective butenolide synthesis through borrowing hydrogen ring contraction.

In our quest to discover new synthetic applications that make use of readily available, commercial enzyme formulations,^44–46^ we recently reported on an isomerization system, where alcohol dehydrogenases convert easy-to-obtain racemic Achmatowicz pyranones to the corresponding optically pure δ-lactones in an enantioconvergent and redox-self-sufficient manner.^47^ Taking inspiration from an iridium-catalyzed protocol,^48^ this biocatalytic interpretation of the classical borrowing hydrogen catalysis opens new opportunities for the utilization of enzymes (Scheme 2a). Nevertheless, the study did not clearly answer mechanistic details (Scheme 2b), such as whether the overall redox-neutral process would proceed through a ketolactone intermediate (3a) *via* oxidation of the pyranone as suggested for the iridium catalysis, or through initial reduction and formation of a transient hydroxylactol (4a). While the former route resembles the typical borrowing hydrogen steps where the catalyst temporarily removes H_2_ equivalents from the substrate and stores them as transition metal hydride species (or a reduced enzyme cofactor),^49–52^ the latter one would constituted an inverse borrowing hydrogen pathway with NAD(P)H being the borrowing entity. Surprisingly, when trying to incorporate the enzymatic methodology into an *in vivo* tool by co-expressing the necessary proteins in a bacterial host, some cellular factories – particularly in a more reductive environment – delivered five-membered aromatic furan products as main metabolites (Scheme 2c).^53^ As 1a is usually obtained through oxidation of furfuryl alcohol 5a in an Achmatowicz oxidation,^54–57^ a retro-Achmatowicz pathway was proposed in which, under strongly reducing conditions, the pyranone substrates are suffering an undesired ring contraction. Biosynthetic studies on the origin of furan metabolites have deciphered pathways where either oxidative or hydrolytic steps provide five-membered hemiacetals that apparently undergo spontaneous dehydration to the aromatic heterocycles (Scheme 2d).^58,59^ In line with this natural precedence, it seemed likely that a reduction-induced isomerization may in fact deliver a furanose intermediate that would dehydrate to the undesired aromatic side product (Scheme 2e). Even if such a retro-Achmatowicz scenario might be of certain academic interest and can in fact be an interesting tool in a cascade reaction setting,^60^ the synthetic value of this particular kind of defunctionalization would be at least questionable. On the other hand, we speculated that interception of the intermediates arising from the proposed pyranose–furanose isomerization would allow us to deviate the biocatalytic pathway and turn the undesired ring contraction to a novel and synthetically more appealing heterocyclic methodology. In our design, we imagined that interruption of the retro-Achmatowicz transformation by dehydrogenation of the five-membered hemiacetal would enable us to produce gamma-lactones in a stereoselective fashion (Scheme 2f). In this overall redox-self-sufficient process – *i.e.* without the need of any sacrificial reductants or oxidants – biomass-derived Achmatowicz pyranones could be directly converted to synthetically highly valuable γ-butenolides. As the reaction would be induced by a dehydrogenase-catalyzed reduction, the racemic, configurationally labile starting materials could be converted to optically enriched products in an enantioconvergent fashion. In order to redirect the original protocol, the catalyst discovery has to focus on a few key aspects. In the first step of the catalysis, high enantioselectivity has to be paired with the inability to irreversibly oxidize the six-membered hydroxylactol 4 further to the δ-lactone (2). While a similar effect could be achieved by modulation of the redox environment (*i.e.* in a stepwise reduction–oxidation scenario), enzymes with the intrinsic deficiency or even better with a certain preference for five-membered furanoses (6) may allow for a true one-pot cascade catalysis. These qualities could of course also be combined in an enzyme-pair with designated redox tasks. Lastly, the reaction conditions themselves (or rapid oxidation of 6 to yield 7) would need to disfavor the undesired dehydration to the competing furans (5).

### Development of an effective method for the redox self-sufficient ring contraction of pyranones

**Scheme 3:**
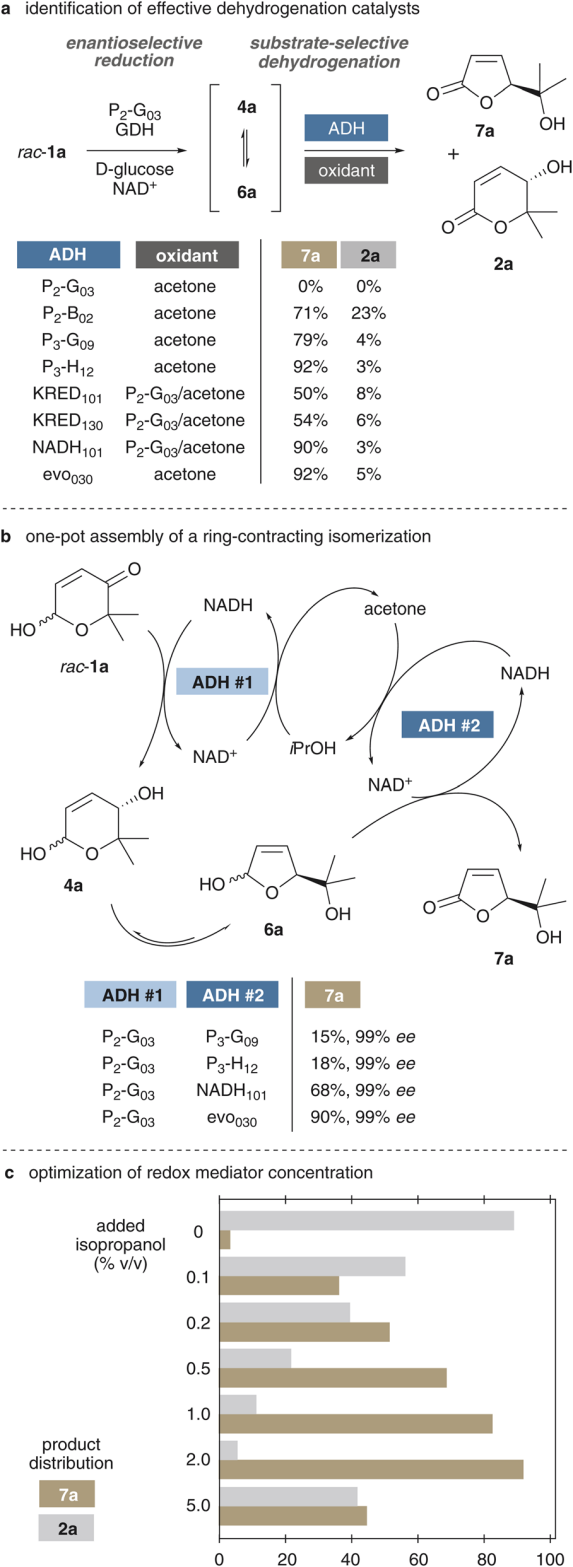
Selection of biocatalysts and redox mediators for the single-step ring-contracting redox isomerization of pyranone 1a to butenolide 7a. Product ratios and yields were determined by GC-FID against bromobenzene as standard.

Based on our previous work on a geometry-conservative redox isomerization,^47^ the commercial alcohol dehydrogenase P_2_-G_03_ from the Codexis KRED enzyme kit was quickly identified as suitable tool for the intended ring-contraction methodology, combining excellent stereoselectivity in the ketoreduction with the inability to engage in subsequent hemiacetal dehydrogenation of 4a/6a in a reductive environment. In a first screening to identify biocatalysts that would be capable of selectively oxidizing the furanose intermediates, model substrate *rac*-1a was initially reduced by means of P_2_-G_03_ together with glucose dehydrogenase and glucose in order to render the reduction fully irreversible. Under these conditions (*rac*-1a (6.5 mM), P_2_-G_03_ (1 mg mL^−1^), GDH (10 U/ml), d-glucose (10 mM), NAD^+^ (2 mM) KP_i_ buffer (1 mL, 50 mM, pH 7.5), 30 °C, 200 rpm), 1a was quickly consumed, without the observation of any of the corresponding lactone products. The reactions were run in a pH 7.5 phosphate buffer to suppress acid-induced dehydration (*i.e.* retro-Achmatowicz reactions). Supplementing the reaction mixture with an excess of acetone as oxidant and a second alcohol dehydrogenase, in selected cases, clean conversion of the presumptive pyranose–furanose mixture to the desired butenolide 7a was observed. Out of a set of 26 commercial biocatalysts, seven ADHs showed significant amounts of lactone products, and most of them actually featured high selectivities for the five-membered product (SI Scheme S1). While most modern commercial alcohol dehydrogenases have been optimized to operate with isopropanol or acetone as cheap co-substrates for direct cofactor recycling by the same enzyme, the enzymes KRED_101_, KRED_130_ and NADH_101_ showed poor performance, so that these biocatalysts were complemented with P_2_-G_03_ to translate the acetone additive into an oxidative environment. Particularly P_3_-H_12_ and evo_030_ stood out with excellent butenolide selectivities and conversions >95% (Scheme 3a). The differences in selectivities (*e.g.* 3 : 1 for P_2_-B_02_*vs.* 30 : 1 for P_3_-H_12_) indicate that the pyranose–furanose interconversion is reversible and that the selectivity in product formation is at least partially governed by catalyst selectivity according to the Curtin–Hammett principle (see also SI Table S1). Interestingly, as opposed to the *in vivo* experiments, the undesired aromatization was not detected under any circumstances. With these promising results in hand, we next aimed for a true cascade catalysis without distinct reduction and oxidation periods, a more challenging approach with seemingly contradictory demands on redox balances of the two individual biocatalysts. Even though the two ADHs may directly interact *via* oxidized or reduced cofactors, the necessity of a moderately reductive environment, to avoid immediate pyranose dehydrogenation to the δ-lactones, prompted us to incorporate a redox mediator into the design (Scheme 3b & SI Scheme S1). Most alcohol dehydrogenases can typically convert acetone to isopropanol, and *vice versa*, *via* consumption of nicotinamide cofactors. We envisaged that isopropanol as additive would thus enable a quick reduction of the pyranone in order to force the formation of the furanose–pyranose mixture. A second dehydrogenase with affinity for the furanose substrate could subsequently employ the resulting acetone to *in situ* generate its oxidized NAD(P)^+^ and irreversibly transform the hemiacetal to the desired butenolide. To our very delight, at 1% isopropanol (v/v) four of the previously identified dehydrogenation biocatalysts paired in a constructive manner with the reductase P_2_-G_03_, giving rise to γ-lactone 7a in enantiopure form. The ADH evo_030_ distinguished itself with a great performance and the P_2_-G_03_/evo_030_/*i*PrOH assembly delivered the butenolide in 90% yield. Further refinement of the system by modulation of the redox mediator revealed the actual impact of isopropanol (Scheme 3c). In absence of any alcohol, the P_2_-G_03_/evo_030_ couple conducted the redox isomerization without significant ring contraction and high selectivity for the δ-lactone (operating through an oxidation-induced pathway, see Scheme 2b). However, already at 0.2% isopropanol, the desired γ-lactone was produced as major entity, and at 2% of the redox mediator, an excellent selectivity of 95% 7a at 99% *ee* was observed. Further increase of the mediator led to overall inhibition of the process and somewhat surprisingly to a loss in selectivity. In theory, it would of course have been possible to further refine the system to employ a single enzyme for both ketoreduction and dehydrogenation, yet, we opted to continue with the P_2_-G_03_/evo_030_ couple to hopefully have a robust methodology at hand without the need for individual enzyme screenings for each structurally related substrate.

### Scope & limitations of the biocatalytic ring contraction

**Scheme 4:**
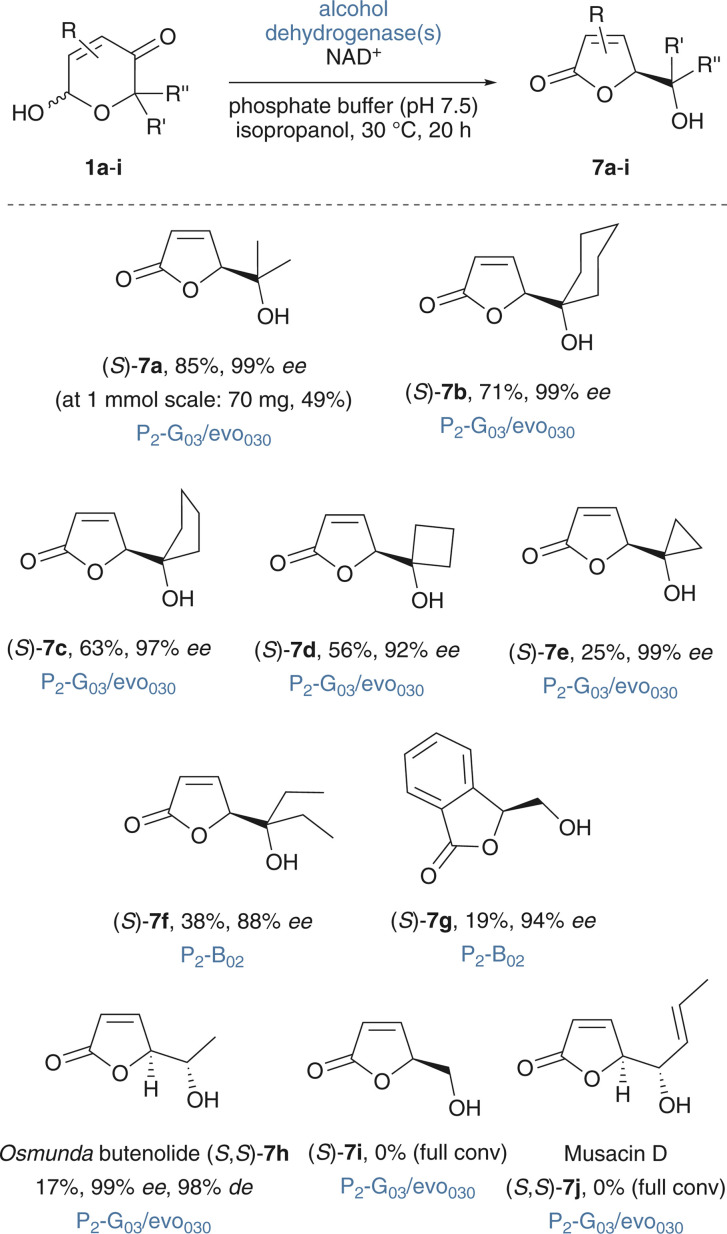
Scope & limitations of the ADH-mediated ring contraction. Reaction conditions: 1 (10–20 mg) P_2_-G_03_/evo_030_ (1.5 mg mL^−1^ each) or P_2_-B_02_ (1.5 mg mL^−1^), NAD^+^ (0.6 mg mL^−1^), *i*PrOH (20 μL mL^−1^) KP_i_ buffer (1 mL mg^−1^1, 50 mM, pH 7.5), 30 °C, 20 h; isolated yields after column chromatography. Absolute configurations were assigned in analogy to the non-contracted δ-lactones 2 which were produced by the same set of dehydrogenases and of which the stereochemistry was studied by vibrational circular dichroism;^47^ all products that were not part of the VCD study were assigned based on HPLC elution order.

Consequently, the new methodology was evaluated in terms of its applicability to structurally related pyranones (Scheme 4). Gratifyingly, a set of cycloalkanol-derived substrates (1b–1e) performed more or less equally well and provided high butenolide selectivities of >88% while maintaining good to excellent enantiomeric purity of the products. The method could also be simply scaled up and 1 mmol of *rac-*1a delivered 70 mg (49%) of the desired butenolide (*S*)-7a. The geminal diethyl-derivative 1f and the aromatic 1g did not react well with the P_2_-G_03_/evo_030_ couple and 7f and 7g were best obtained with an alternative single-enzyme option, in which the alcohol dehydrogenase P_2_-B_02_ acts both as ketoreductase and as dehydrogenation catalyst for the hemiacetal-to-butenolide conversion. In both cases, high enantioselectivities were achieved but selectivities for the five-membered *O*-heterocyclic products remained lower, and significant amounts of the δ-lactone side products were observed. As previously mentioned, γ-butenolides are highly attractive synthesis targets and the structural motif is widespread in natural bioactive secondary metabolites. Obviously, we were eager to also test the applicability of the ring contraction approach as tool in natural product synthesis. The methyl-substituted pyranone 1h was easily obtained *via* oxidative ring expansion of the corresponding furfural-derived optically pure secondary alcohol, and we were very pleased to find that the P_2_-G_03_/evo_030_ couple mediated the redox isomerization under ring contraction. Hence, the new methodology was successfully applied for the stereoselective synthesis the plant-derived butenolide 7h that was previously isolated from several *Osmunda* species,^61,62^ albeit at only moderate yield. On the other hand, attempts to perform ring contractions of 1i and 1j remained unsuccessful and the isomerization of 1j to musacin D (7j), a fungal metabolite found in *Sepedonium chrysospermum*,^63^ stalled instead on the hemiacetal stage (>99% conv. of 1j).

**Scheme 5:**
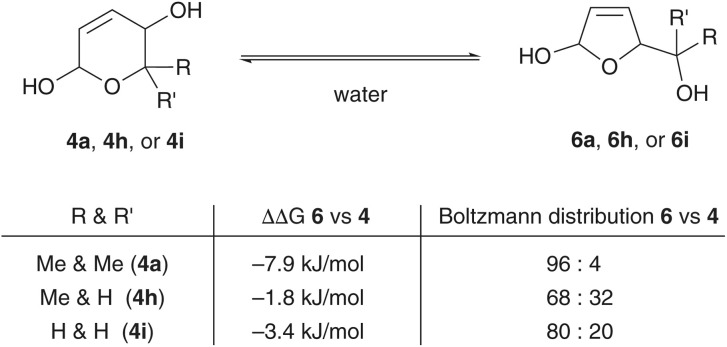
*Ab initio* energy calculations provide insights on preferences and biases in the pyranose–furanose isomerization.

Even though we believe that the process relies on a substrate-selective dehydrogenation catalyst with preference for the oxidation of the furanose intermediate (SI Table S1), we were uncertain whether the general thermodynamic bias for one hemiacetal over another may play a critical role in statistically disfavoring the ring contraction as in for example the case of 1i or 1j. In absence of detailed spectroscopic data on the furanose–pyranose mixtures, we turned to computational tools to gain insights. DLPNO-CCSD(T) *ab initio* energy calculations on DFT optimized structures revealed that in the comparative study involving the reduced intermediates of the pyranone substrates 1a, 1h, and 1i, the ring-contracted furanose (6) would be favored in all cases over the pyranose (3), yet with no clear or decisive bias (Scheme 5). It seems therefore more plausible that not energetics or a lack of selectivity of the dehydrogenative enzymes prohibits from effective ring contractions. In all low yielding or non-productive examples, it is worth noting that primary or secondary alcohol groups would be liberated during the ring contraction (see also SI Scheme S1). These species, however, are no longer innocent spectators but can engage in ADH-mediated dehydrogenations leading to aldehyde/ketone side products and skewing the overall redox balance (with the accumulation of NAD(P)H). This undesired removal of the oxidizing nicotinamide cofactor would thus prevent hemiacetal dehydrogenation and inhibit the completion of the redox self-sufficient catalysis cycle.

**Scheme 6:**
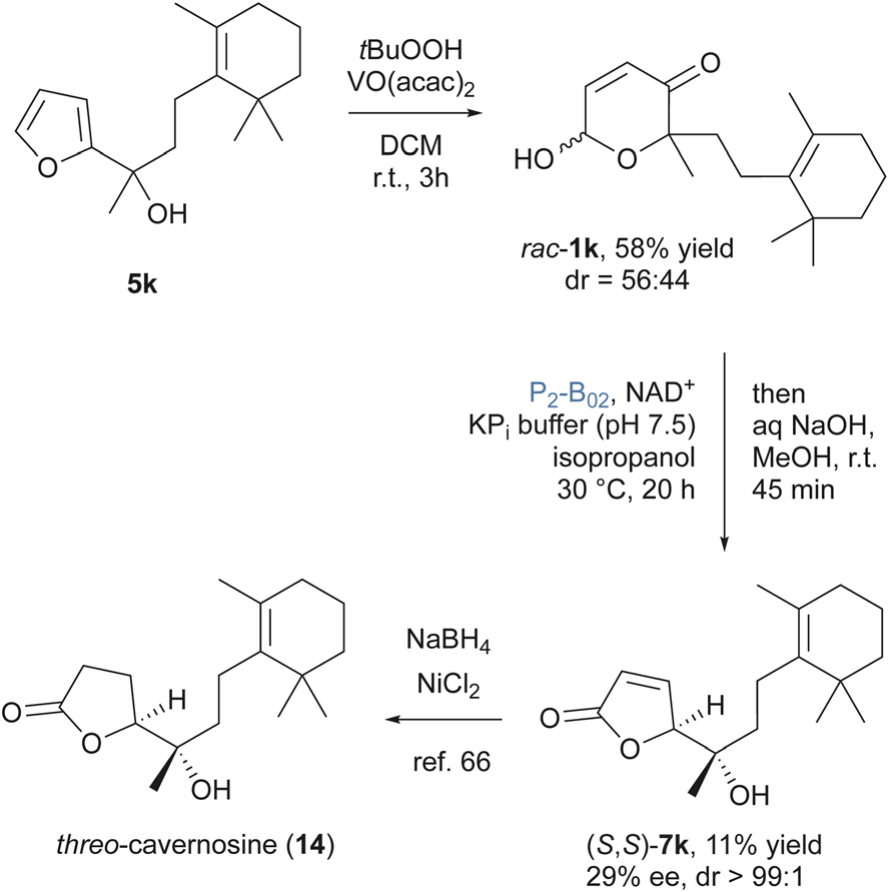
Chemoenzymatic formal total synthesis of *threo*-cavernosine.

Based on these insights, we refocused on possible target structures matching the favored substitution patterns and delivering butenolides with redox-inactive tertiary alcohol side chains. To further investigate the synthetic scope and limitations of the ring contraction strategy, a total synthesis approach based on this key transformation was designed to address the cavernosine terpenoid family.^64,65^ The terpenoid cavernosine (14) was isolated from marine sponges of the *Fasciospongia* and *Cacospongia* genera. It features the characteristic tertiary alcohol group as sole substituent in the 5-position of a γ-lactone, and we aimed to produce the butenolide 7k that represents the direct precursor of caverosine in Jefford's total synthesis (Scheme 6).^66^ In analogy to our other substrates, the key intermediate *rac*-1k was obtained from the corresponding furan 5k through Achmatowicz oxidation. Utilizing a directed protocol employing VO(acac)_2_ and *t*BuOOH under Sharpless epoxidation conditions, to avoid oxidation of the highly nucleophilic cyclohexene moiety, delivered the racemic pyranone in a decent yield of 58%. In contrast to the other tested pyranones, 1k poses a serious challenge for the alcohol dehydrogenase biocatalysts as the stereogenic center introduces the sterically highly demanding trimethylcyclohexene side chain. A small set of ADHs was tested and only P_2_-B_02_ facilitated conversion of 1k at acceptable rates. Possibly as a result of the bulky nature of the substrate, in addition to the desired butenolide 7k, also the corresponding δ-lactone 2k and the retro-Achmatowicz product 5k were observed. With the synthetic goal in mind, without purification, we opted to conduct an immediate base-induced transformation of the residual 2k to convert all isomerization products to 7k, which was isolated in modest 11% yield. Strikingly, however, the biotransformation of 1k proceeded with an alternative mode of stereocontrol. Apparently, the bulky terpene side chain imposes a strong induction to overwrite the enzyme's native face selectivity, so that the dehydrocarvernosine 7k was isolated as single diastereomer, with only moderate enantiopurity of 29% *ee*. Comparison with the spectral data of Jefford's study, we could conclude that this chemoenzymatic strategy thus enables the preparation of *threo*-cavernosine. Future investigations will look further into this highly interesting stereoinduction competition in order to identify more selective dehydrogenases for a more effective kinetic resolution process.

### Enzymatic ring contraction modules complement the biocatalytic machinery to assemble synthetic multi-step cascades

**Scheme 7:**
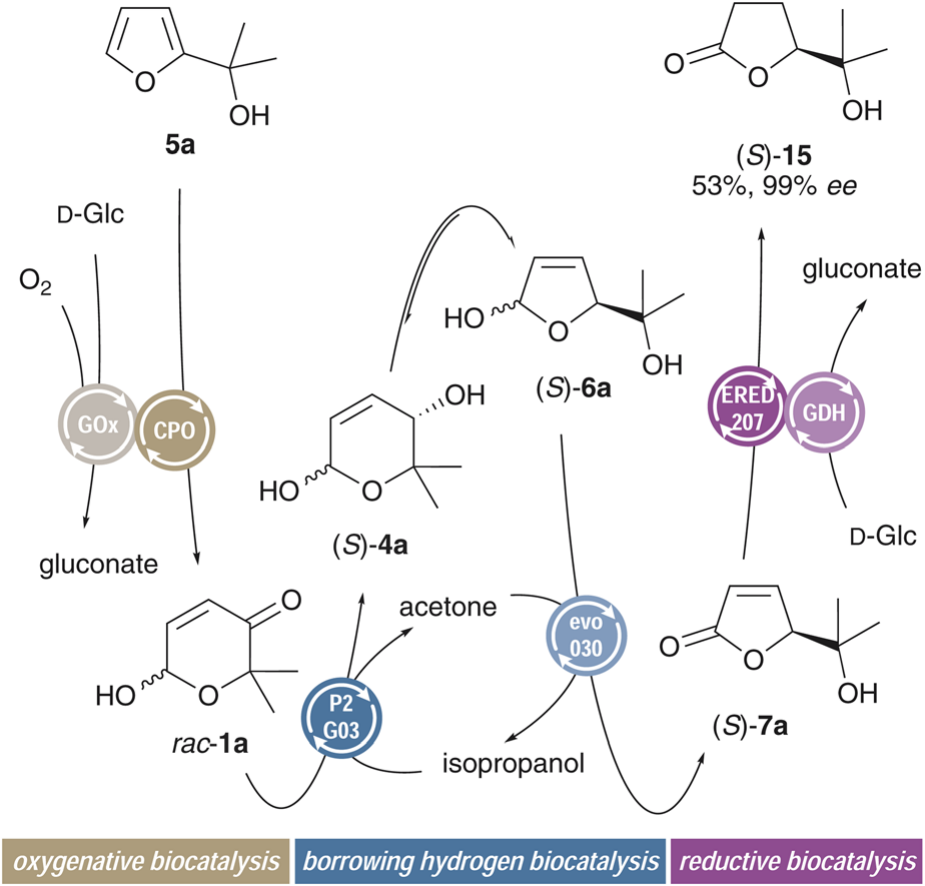
Assembly of a formal enantioselective dearomatization of furfuryl alcohols with the borrowing hydrogen ring contraction as central element in a multi-enzyme cascade.

As the Achmatowicz pyranones can be directly synthesized from the lignocellulose-derived five-membered furfuryl alcohols, it is easy to imagine a scenario where the six-membered O-heterocycles would only act as intermediate in a multi-catalytic cascade. The combination of a ring-expanding Achmatowicz oxidation with the herein presented ring-contracting borrowing hydrogen redox isomerization and a final enoate reduction would thus render an overall process in which the furan starting materials could be dearomatized in an enantioselective fashion through a transient pyranone. One of the major benefits of biocatalysis in general is the excellent mutual tolerance of different enzymes and the opportunity to merge multiple biocatalytic functions in a one-pot fashion, thereby eliminating isolation and purification steps that typically contribute to unnecessary product loss and overall poor sustainability factors. Thus, more step-economic and elegant catalytic cascades can be implemented (Scheme 7). Best results were obtained in a sequential biocatalysis setup where furfuryl alcohol 5a was first incubated with chloroperoxidase and glucose oxidase in order to furnish the pyranone intermediate. After adjustment of the pH of the buffer and addition of P_2_-G_03_ and evo_030_, clean formation of the dearomatized five-membered O-heterocycle was observed and 7a was generated in enantiopure form, whereupon addition of an enoate reductase (ERED_207_) and glucose dehydrogenase delivered the saturated γ-lactone 15 in excellent optical purity. Here, an overall yield of 53% translates to an average yield per chemical step of 81%. The sequential three-step one-pot process involving six distinct enzyme catalysts thus enables a streamlined preparation of a saturated O-heterocyclic building block through a formal enantioselective water addition to the biorefinery waste furan, where the central borrowing hydrogen biocatalysis is complemented with both oxidative and reductive enzyme modules.

## Conclusion

In summary, we herein report on the development of a new application of alcohol dehydrogenase enzymes as biocatalysts for the redox-neutral isomerization of racemic Achmatowicz pyranones to enantioenriched butenolides. The methodology relies on a reduction–oxidation sequence, similar to the classical borrowing hydrogen catalysis principle, where isopropanol acts as initial hydrogen source and where the dehydrogenase stores an oxidation equivalent in form of acetone as redox mediator, which after ring contraction of the resulting pyranose to a furanose can be utilized in an irreversible dehydrogenation to yield the five-membered lactone products. This unprecedented type of ring contraction performs particularly well on 6,6-disubstituted pyranones and the resulting butenolides can be obtained in high yields and with excellent optical purities. The biocatalytic module cannot only be employed as stand-alone asymmetric synthesis tool but is also well compatible with enzymatic cascades as illustrated in the valorization of a biomass-derived furan to an enantiopure γ-butyrolactone through a six-enzyme catalysis sequence. Future studies on this system will address alternative redox mediator systems to overcome limitations regarding the production of γ-lactones bearing primary and secondary alcohol functionalities, as well as broader utilization in the natural product synthesis of more complex lactone metabolites.

## Author contributions

Conceptualization: YCL, AOC, JD; methodology, investigation, data curation: YCL, AOC; formal analysis: JDR; project administration, resources, funding acquisition: JD; writing, visualization: YCL, JD.

## Conflicts of interest

The authors do not declare any competing interests.

## Supplementary Material

SC-016-D5SC02591E-s001

## Data Availability

Primary data for this article, including NMR fid's and chromatograms are available at Zenodo at https://doi.org/10.5281/zenodo.16910331. The data supporting this article have been included as part of the SI. Supplementary information is available. See DOI: https://doi.org/10.1039/d5sc02591e.

## References

[cit1] Zhang X.-M., Tu Y.-Q., Zhang F.-M., Chen Z.-H., Wang S.-H. (2017). Chem. Soc. Rev..

[cit2] Jurczyk J., Lux M. C., Adpressa D., Kim S. F., Lam Y., Yeung C. S., Sarpong R. (2021). Science.

[cit3] Zhang Z., Qian X., Gu Y., Gui J. (2024). Nat. Prod. Rep..

[cit4] Sharma R., Arisawa M., Takizawa S., Salem M. S. H. (2025). Org. Chem. Front..

[cit5] Hui C., Craggs L., Antonchick A. P. (2022). Chem. Soc. Rev..

[cit6] Silva L. F. (2002). Tetrahedron.

[cit7] Chen L., Li G., Zu L. (2022). Org. Chem. Front..

[cit8] Creary X. (1991). Chem. Rev..

[cit9] Wakchaure V. N., DeSnoo W., Laconsay C. J., Leutzsch M., Tsuji N., Tantillo D. J., List B. (2024). Nature.

[cit10] Song Z.-L., Fan C.-A., Tu Y.-Q. (2011). Chem. Rev..

[cit11] Kirmse W. (2002). Eur. J. Org Chem..

[cit12] Lee E., Yoon C. H. (1994). J. Chem. Soc. Chem. Commun..

[cit13] Jamison T. F., Shambayati S., Crowe W. E., Schreiber S. L. (1997). J. Am. Chem. Soc..

[cit14] Burke A. J., Moutayakine A. (2021). Chem. Commun..

[cit15] Nicolaou K. C., Sarlah D., Wu T. R., Zhan W. (2009). Angew. Chem., Int. Ed..

[cit16] Song Z., Meng S., Wang Q. (2022). ChemistrySelect.

[cit17] Pasetto P., Naginskaya J. (2018). Tetrahedron Lett..

[cit18] Becker K. B., Labhart M. P. (1983). Helv. Chim. Acta.

[cit19] Schultz P. G., Dervan P. B. (1981). J. Am. Chem. Soc..

[cit20] Hui C., Brieger L., Strohmann C., Antonchick A. P. (2021). J. Am. Chem. Soc..

[cit21] Kennedy S. H., Dherange B. D., Berger K. J., Levin M. D. (2021). Nature.

[cit22] Hui C., Wang S., Xu C. (2022). Chin. Chem. Lett..

[cit23] Isaji H., Sako K., Takemura H., Tatemitsu H., Shinmyozu T. (1998). Tetrahedron Lett..

[cit24] Nicolaou K. C., Gray D., Tae J. (2001). Angew. Chem., Int. Ed..

[cit25] Nicolaou K. C., Gray D. L. F., Tae J. (2004). J. Am. Chem. Soc..

[cit26] Natarajan A., Ng D., Yang Z., Garcia-Garibay M. A. (2007). Angew. Chem., Int. Ed..

[cit27] Savino S., Borg A. J. E., Dennig A., Pfeiffer M., De Giorgi F., Weber H., Dubey K. D., Rovira C., Mattevi A., Nidetzky B. (2019). Nat. Catal..

[cit28] Moore S. J., Biedendieck R., Lawrence A. D., Deery E., Howard M. J., Rigby S. E. J., Warren M. J. (2013). J. Biol. Chem..

[cit29] Kishimoto S., Hara K., Hashimoto H., Hirayama Y., Champagne P. A., Houk K. N., Tang Y., Watanabe K. (2018). Nat. Commun..

[cit30] Ondeyka J. G., Zink D., Basilio A., Vicente F., Bills G., Diez M. T., Motyl M., Dezeny G., Byrne K., Singh S. B. (2007). J. Nat. Prod..

[cit31] Kong F., Carter G. T. (2003). Tetrahedron Lett..

[cit32] Nie Q., Sun C., Liu S., Li Q., Zotova M., Zhu T., Gao X. (2025). J. Am. Chem. Soc..

[cit33] Nagel R., Alexander L. E., Stewart C. E., Peters R. J. (2023). Proc. Natl. Acad. Sci. U. S. A..

[cit34] Bajpai L. K., Bhaduri A. P. (1996). J. Mol. Catal. B: Enzym..

[cit35] Guo Y.-Y., Tian Z.-H., Ma C., Han Y.-C., Bai D., Jiang Z. (2023). Chem. Sci..

[cit36] Manzuna Sapu C., Bäckvall J.-E., Deska J. (2011). Angew. Chem., Int. Ed..

[cit37] Manzuna Sapu C., Deska J. (2013). Org. Biomol. Chem..

[cit38] Naapuri J., Rolfes J. D., Keil J., Manzuna Sapu C., Deska J. (2017). Green Chem..

[cit39] Skrobo B., Schlörer N. E., Neudörfl J.-M., Deska J. (2018). Chem.–Eur. J..

[cit40] Naapuri J. M., Åberg G. A., Palomo J. M., Deska J. (2021). ChemCatChem.

[cit41] Naapuri J. M., Losada-Garcia N., Rothemann R. A., Pichardo M. C., Prechtl M. H. G., Palomo J. M., Deska J. (2022). ChemCatChem.

[cit42] Naapuri J. M., Wagner P. K., Hollmann F., Deska J. (2022). ChemistryOpen.

[cit43] Naapuri J. M., Losada-Garcia N., Deska J., Palomo J. M. (2022). Nanoscale.

[cit44] Thiel D., Doknić D., Deska J. (2014). Nat. Commun..

[cit45] Jäger C., Haase M., Koschorreck K., Urlacher V. B., Deska J. (2023). Angew. Chem., Int. Ed..

[cit46] Kiefer A. F., Liu Y.-C., Gummerer R., Jäger C., Deska J. (2023). Angew. Chem., Int. Ed..

[cit47] Liu Y.-C., Merten C., Deska J. (2018). Angew. Chem., Int. Ed..

[cit48] Wang H.-Y., Yang K., Bennett S. R., Guo S., Tang W. (2015). Angew. Chem., Int. Ed..

[cit49] Hamid M. H. S. A., Allen C. L., Lamb G. W., Maxwell A. C., Maytum H. C., Watson A. J. A., Williams J. M. J. (2009). J. Am. Chem. Soc..

[cit50] Corma A., Navas J., Sabater M. J. (2018). Chem. Rev..

[cit51] Reed-Berendt B. G., Latham D. E., Dambatta M. B., Morrill L. C. (2021). ACS Cent. Sci..

[cit52] Alexandridis A., Quintard A. (2024). ChemCatChem.

[cit53] Liu Y.-C., Wu Z.-L., Deska J. (2023). ChemSusChem.

[cit54] Achmatowicz O., Bukowski P., Szechner B., Zwierzchowska Z., Zamojski A. (1971). Tetrahedron.

[cit55] Lefebvre Y. (1972). Tetrahedron Lett..

[cit56] Deska J., Thiel D., Gianolio E. (2015). Synthesis.

[cit57] Ghosh A. K., Brindisi M. (2016). RSC Adv..

[cit58] Bertea C. M., Schalk M., Karp F., Maffei M., Croteau R. (2001). Arch. Biochem. Biophys..

[cit59] Zhou S., Malet N. R., Song L., Corre C., Challis G. L. (2020). Chem. Commun..

[cit60] Zhang X., Tong Y., Li G., Zhao H., Chen G., Yao H., Tong R. (2022). Angew. Chem., Int. Ed..

[cit61] Carpinteyro Diaz A. E., Herfindal L., Holmelid B., Brede C., Andersen H. L., Vedeler A., Fossen T. (2024). Molecules.

[cit62] Feng X., Cai M., Zhu L., Zheng S., Chen X. (2024). Nat. Prod. Res..

[cit63] Mitova M. I., Stuart B. G., Cao G. H., Blunt J. W., Cole A. L. J., Munro M. H. G. (2006). J. Nat. Prod..

[cit64] Braekman J. C., Daloze D., Bertau R., Macedo De Abreu P. (1982). Bull. Soc. Chim. Belg..

[cit65] Zhang X., Li P.-L., Qin G.-F., Li S., De Voogd N. J., Tang X.-L., Li G.-Q. (2019). Mar. Drugs.

[cit66] Jefford C. W., Jaggi D., Bernardinelli G., Boukouvalas J. (1987). Tetrahedron Lett..

