# Supplementary material for: Asymmetric ring contraction of 2-hydroxypyranones by borrowing hydrogen biocatalysis
Source: Chem Sci. 2025 Aug 29;16(38):17667–74. doi: 10.1039/d5sc02591e (PMC12418039; doi:10.1039/d5sc02591e)
Supplement: SC-016-D5SC02591E-s001 [file SC-016-D5SC02591E-s001.pdf]

Supplementary Information (SI) for Chemical Science.
This journal is © The Royal Society of Chemistry 2025

# Asymmetric Ring Contraction of 2-Hydroxypyranones by Borrowing Hydrogen Biocatalysis

*Yuchang Liu*, Adam O'Connell, J. D. Rolfes, Jan Deska**

National Engineering Laboratory for Industrial Enzymes and Tianjin Engineering Research Center of Biocatalytic Technology, National Center of Technology Innovation for Synthetic Biology, Tianjin 300308, PR China

Tianjin Institute of Industrial Biotechnology, Chinese Academy of Sciences, Tianjin 300308, PR China

liuych@tib.cas.cn

Department of Chemistry, University of Helsinki, A.I. Virtasen aukio 1, 00560 Helsinki, Finland

jan.deska@helsinki.fi

**Table of contents**

General remarks……………………………..…………………..……2

Catalyst screening and general enzyme cascade design……..…….3

Computational Molecular Calculations………………………..…….5

Synthesis and analysis of products……………………………...…11

Preparation of substrates……………………………………………16

NMR spectra of the products……………………....…………………18

HPLC and GC traces…………………………………………..……..30

Supplementary references…………………………..……..……..39

**General Remarks**

Alcohol dehydrogenases were purchased from Codexis (Codex Ketoreductase Screening Kit) and from evocatal GmbH. Ene reductases were purchased from Codexis. Nicotinamide cofactors were obtained from Carbolution Chemicals GmbH. Glucose dehydrogenase (*Pseudomonas* sp.) was purchased from Sigma Aldrich. All reactions that were carried out under argon atmosphere were performed with dry solvents using anhydrous conditions. Dry solvents were taken from a solvent drying system MB-SPS-800 from M-Braun. Commercially available reagents were used without further purification. Enzymatic reactions were performed under non-inert conditions on an orbital shaker in caped glass vials. Column chromatography was performed with silica gel from Merck (Millipore 60, 40-60 µm, 240-400 mesh). Reactions were monitored by thin layer chromatography (TLC) on Machery-Nagel precoated silica gel plates (TLC Silica gel 60 F254). The TLC plates were visualized using UV light and staining with a basic potassium permanganate solution. $^{1}H$ and $^{13}C$ NMR-spectra were recorded on a Bruker AV-400 instrument at 20 °C. Chemical shifts are reported in parts per million (ppm) calibrated using residual non-deuterated solvents as internal reference [$CHCl_3$ at δ = 7.26 ppm ($^{1}H$ NMR) and 77.16 ppm ($^{13}C$ NMR). Infrared spectra were recorded on a Bruker ALPHA Eco-ATR spectrometer, absorption bands are reported in wave numbers [$cm^{-1}$]. High-resolution mass spectrometry was performed on an Agilent 6530 (Q-TOF) mass spectrometer. Optical rotations were measured on an Autopol VI – automatic polarimeter from Rudolph Research Analytical. Gas chromatography was performed on a Hewlett Packard HP 6890 Series GC System using a Beta DEX$^{TM}$ 120 fused silica capillary column (30 m x 0.25 mm), Helium, 1.0 ml/min; temperature program: 50 °C (5 min) / 5 °C · $min^{-1}$ / 110 °C (10 min)/ 5 °C · $min^{-1}$ / 10 °C $min^{-1}$ / 180°C (6 min) / 10 °C · $min^{-1}$ / 200 (20 min). High-performance liquid chromatography analysis was performed on an Agilent 1100 system with a G1312A binary pump and a G1312B diode array detector using analytical Daicel Chiralpak column (250 mm x 4.6 mm; AD). Absolute configurations were assigned in analogy to the non-contracted delta-lactones **2** which were produced by the same set of dehydrogenases and of which the stereochemistry was studied by vibrational circular dichroism;[1] all products that were not part of the VCD study were assigned based on HPLC elution order.

## Catalyst screening and general enzyme cascade design

*Screening of catalysts for the dehydrogenation of 3a/6a*

In a 2 ml Eppendorf tube, *rac*-**1a** (6.5 mM) was dissolved in phosphate buffer (1 ml, 50 mM, pH 7.5), $P_2$-$G_{03}$ (1 mg/mL), glucose dehydrogenase (0.1 mg/mL), D-glucose (10 mM) and $NAD^+$ (2 mM) were added and the solution was incubated at 30 °C, 200 rpm for 10 h. To the same reaction mixture, alcohol dehydrogenase (2 mg/mL) and acetone (67 µmol, 67 mM, 5 µL) was added, followed by incubation at 30 ºC, 200 rpm for 15 h on an incubation shaker. The reaction mixture was extracted with ethyl acetate (3 x 0.5 ml). The combined organic phases were dried over anhydrous $Na_2SO_4$, concentrated under reduced pressure and re-dissolved into ethyl acetate (0.5 ml) containing the internal standard bromobenzene (5 mM). **GC** (50 °C (5 min) /5 °C $min^{-1}$ / 110 °C (10 min) /5 °C $min^{-1}$ /10 °C $min^{-1}$ / 180°C (6 min) / 10 °C $min^{-1}$ /200 (20min)): $t_R$ (**1a**) = 32.4 min, $t_R$ (**7a**) = 36.7 min, $t_R$ (**2a**) = 37.3 min.

**Supplementary Table S1** Screening of catalysts for the biocatalytic dehydrogenation of **3a**/**6a**.

| enzyme | cofactor regeneration | δ-lactone (2a) | γ-lactone (7a) | |
|---|---|---|---|---|
| | | Yield (%) | Yield (%) | ee (%) |
| $P_1$-$A_{04}$ | acetone | 0 | 0 | – |
| $P_1$-$B_{02}$ | acetone | 18 | 0 | – |
| $P_3$-$H_{12}$ | acetone | 3 | 92 | 99 |
| $P_1$-$B_{05}$ | acetone | 0 | 0 | – |
| $P_2$-$H_{07}$ | acetone | 0 | 0 | – |
| $P_3$-$G_{09}$ | acetone | 4 | 79 | 99 |
| $P_1$-$B_{12}$ | acetone | 0 | 7 | 99 |
| $evo_{030}$ | acetone | 5 | 92 | 99 |
| $evo_{200}$ | acetone | 0 | 0 | – |
| $P_2$-$D_{12}$ | acetone | 2 | 11 | 99 |
| $P_1$-$H_{10}$ | acetone | 0 | 0 | – |
| $P_2$-$B_{02}$ | acetone | 71 | 23 | 99 |
| $P_2$-$C_{02}$ | acetone | 25 | 8 | 99 |
| $P_2$-$C_{11}$ | acetone | 0 | 0 | – |
| $P_1$-$B_{10}$ | acetone | 13 | 9 | 99 |
| $P_2$-$D_{03}$ | acetone | 32 | 9 | 99 |
| $P_1$-$H_{08}$ | acetone | 6 | 10 | 99 |
| $P_2$-$D_{11}$ | acetone | 0 | 5 | 99 |
| $P_1$-$C_{01}$ | acetone | 0 | 4 | – |
| $P_2$-$G_{03}$ | acetone | 0 | 0 | – |
| $P_3$-$B_{03}$ | acetone | 0 | 0 | – |
| $NADH_{101}$[a] | $P_2$-$G_{03}$ / acetone | 3 | 90 | 99 |
| $NADH_{110}$[a] | $P_2$-$G_{03}$ / acetone | 12 | 2 | 99 |
| $KRED_{101}$[a] | $P_2$-$G_{03}$ / acetone | 8 | 50 | 99 |
| $KRED_{119}$[a] | $P_2$-$G_{03}$ / acetone | 0 | 0 | – |
| $KRED_{130}$[a] | $P_2$-$G_{03}$ / acetone | 6 | 54 | 99 |

[a] Cofactor $NAD^+$ was regenerated from acetone by the addition of $P_2$-$G_{03}$ (1 mg/ml).

*Detailed relationship of substrates & cofactors*

The following scheme complements the reaction pathway from Scheme 3b, to highlight the reversibility and irreversibility of individual steps, as well as undesired side reactions that may occur with primary and secondary alcohol derivatives.

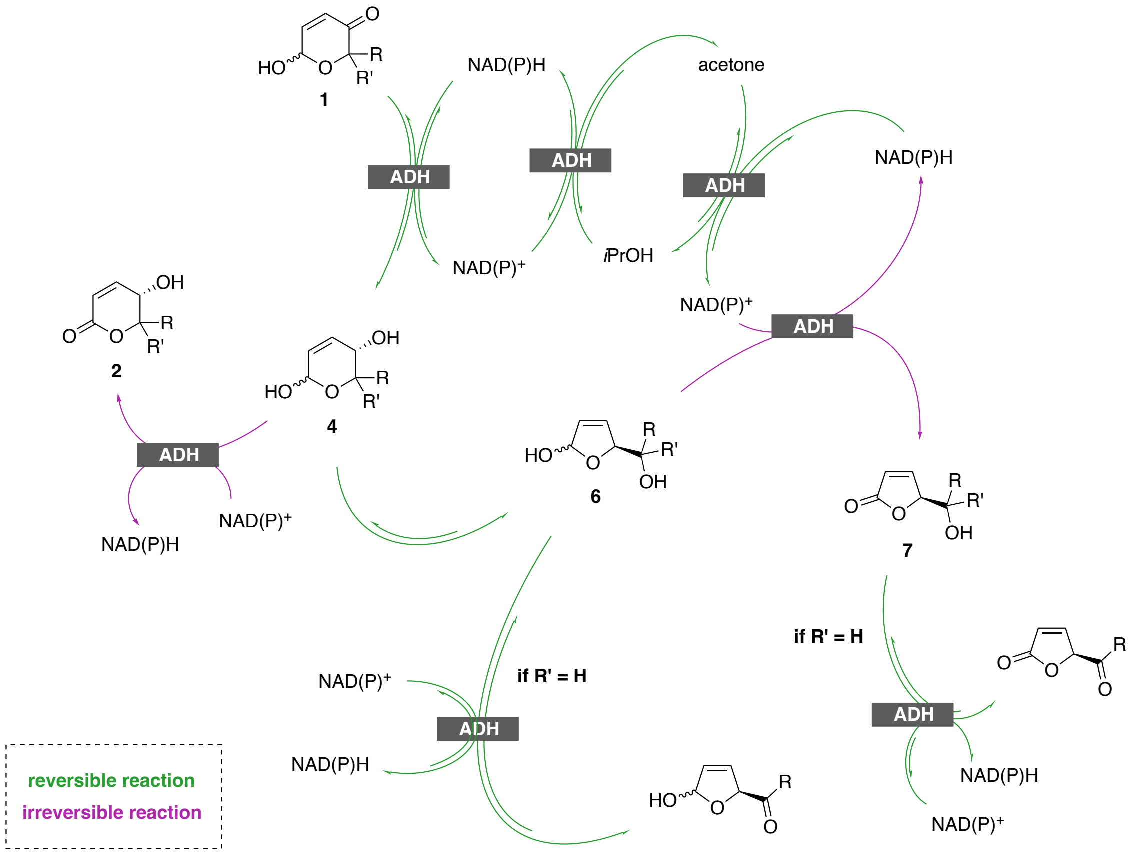


**Supplementary Scheme 1** Overview on all possible transformations of substrates, intermediates and products through ADH-mediated oxidations/reductions.

## Computational Molecular Calculations

**Computational Methods:** All computations were performed with the ORCA program package.[2,3] Geometry optimizations were performed as density functional theory (DFT) calculations with the PBE0 functional [4] and the def2-TZVP basis set [5] including Grimme's D3 dispersion correction with the Becke–Johnson damping scheme (ORCA keyword "D3").[6,7] The resolution of identity approximation [8] was used for the Coulomb integrals with the def2/J auxiliary basis set [9] and for the Hartree-Fock exchange terms with the chain of spheres (COSX) approximation.[10] Starting geometries were built from scratch using the molecular builder in the Avogadro[11] program. Local minima were confirmed through frequency analysis. Self-consistent field (SCF) and optimization convergence criteria were set tightly (ORCA keywords "TightSCF" and "TightOpt").

For single point energies, domain-based pair natural orbital coupled cluster calculations with single and double, and perturbative triple excitations (DLPNO-CCSD(T))[12-16] were performed, with the def2-QZVPP basis sets[4] and def2-QZVPP/C auxiliary basis sets.[17] SCF convergence criteria were set tightly, and solvation in water was modelled with the solvation model based on density (SMD).[18]

Gibbs Free Energies were calculated by addition of the thermal corrections of Gibbs Free Energy from the DFT geometry optimization calculations to the electronic energies of the single point calculations.

**Geometries and Energies:**

**6a**

| | | | |
|---|---|---|---|
| O | 0.0000000000 | 1.4171052518 | 0.0000000000 |
| O | -1.8030385761 | 1.7858776944 | 2.0014442700 |
| O | 0.0468806167 | -0.5380356883 | 1.2904044290 |
| C | -3.3833684317 | 2.8964465014 | 0.6527446788 |
| C | -1.1488997299 | 3.9256584259 | 1.1218240572 |
| C | -1.2669263590 | -0.3649022323 | -0.7104343434 |
| C | -2.0182796498 | 0.7144535643 | -0.8541363969 |
| C | -1.2837029349 | 1.9139904143 | -0.3530679197 |
| C | -1.9079350854 | 2.6312263550 | 0.8675744644 |
| C | 0.0000000000 | 0.0000000000 | 0.0000000000 |
| H | -1.2826395324 | 4.6288890859 | 0.2964414596 |
| H | -1.5137121423 | 4.3912418525 | 2.0389538038 |
| H | -0.0798154003 | 3.7267244302 | 1.2280094570 |
| H | -3.9414982362 | 1.9596797867 | 0.6278328537 |
| H | -3.7743802008 | 3.5038461146 | 1.4705492541 |
| H | -3.5489326639 | 3.4335020056 | -0.2841199737 |

| | | | |
|---|---|---|---|
| H | -1.1537024699 | 2.6535144703 | -1.1544078719 |
| H | -0.8364761475 | -0.4410826075 | 1.6684265000 |
| H | -0.8587047762 | 1.6700621226 | 2.1625589519 |
| H | 0.9212812566 | -0.3322489840 | -0.4840075406 |
| H | -1.5166005334 | -1.3843093959 | -0.9704718773 |
| H | -3.0143082784 | 0.7586845603 | -1.2721104522 |

Electronic Energy:

-498.996300344827

Correction to Gibbs Free Energy:

0.15331604

**4a**

| | | | |
|---|---|---|---|
| O | -2.0802532138 | 2.4052651546 | -1.9590041061 |
| O | 1.1975499450 | -0.4279356250 | -0.5616329501 |
| O | -0.0000000000 | 1.4055349207 | 0.0000000000 |
| C | -1.1890986674 | -0.5522388097 | -0.7259724798 |
| C | -2.2258204093 | 0.2023504435 | -1.0652823582 |
| C | -2.2668913455 | 1.6705318436 | -0.7621424144 |
| C | -1.2491315731 | 2.0233956741 | 0.3335939573 |
| C | 0.0000000000 | 0.0000000000 | 0.0000000000 |
| C | -1.7517307330 | 1.5609670551 | 1.6965811642 |
| C | -0.9612700058 | 3.5087835892 | 0.3685279950 |
| H | -1.2297360342 | 2.1362911338 | -2.3209776086 |
| H | -3.2646079906 | 1.9526364916 | -0.4120249441 |
| H | 1.2215162450 | -0.1108470289 | -1.4708369428 |
| H | 0.0244293603 | -0.3801205119 | 1.0315032618 |
| H | -1.1488473477 | -1.6109939657 | -0.9615557830 |
| H | -3.0589125703 | -0.2117459467 | -1.6245093302 |
| H | -2.0566923064 | 0.5131060164 | 1.6962283793 |
| H | -2.6151090555 | 2.1592427804 | 1.9933932188 |
| H | -0.9694709711 | 1.6994835236 | 2.4451646962 |

| | | | |
|---|---|---|---|
| H | -0.5409599121 | 3.8444906327 | -0.5778815345 |
| H | -0.2502453643 | 3.7242218484 | 1.1677058095 |
| H | -1.8798120568 | 4.0695052169 | 0.5515701379 |

Electronic Energy:

-498.993266775400

Correction to Gibbs Free Energy:

0.15327967

**6h**

| | | | |
|---|---|---|---|
| O | 0.0000000000 | 1.4176928659 | 0.0000000000 |
| O | -1.8627254200 | 1.7586819067 | 1.9876780363 |
| O | 0.0459814636 | -0.5375129981 | 1.2902772084 |
| C | -1.2748449220 | 3.9425570439 | 1.1372236814 |
| C | -1.2687246850 | -0.3671364652 | -0.7077350192 |
| C | -2.0196090594 | 0.7122193934 | -0.8520875547 |
| C | -1.2834551715 | 1.9115679696 | -0.3560463893 |
| C | -1.9256431114 | 2.6025259215 | 0.8573247358 |
| C | 0.0000000000 | 0.0000000000 | 0.0000000000 |
| H | -1.4071633468 | 4.6269809413 | 0.2958952245 |
| H | -1.7143229703 | 4.3951194942 | 2.0271029523 |
| H | -0.2014783460 | 3.8140034857 | 1.3024742032 |
| H | -1.1550935843 | 2.6536943987 | -1.1551150414 |
| H | -0.8386070647 | -0.4416867084 | 1.6658515883 |
| H | -0.9256025130 | 1.6676931593 | 2.1994299387 |
| H | 0.9215451343 | -0.3319544493 | -0.4836131616 |
| H | -1.5215918352 | -1.3884385025 | -0.9568300722 |
| H | -3.0241294271 | 0.7603668086 | -1.2515047051 |
| H | -2.9911071484 | 2.7430632014 | 0.6490668968 |

Electronic Energy:

-459.739688501758

Correction to Gibbs Free Energy:

0.12680232

**4h**

| | | | |
|---|---|---|---|
| O | 0.0000000000 | 1.4067279059 | 0.0000000000 |
| O | 1.1873235604 | -0.4417257030 | -0.5647939317 |
| O | -2.1753830992 | 2.4920028524 | -1.7711656722 |
| C | -1.0452546051 | 3.4086993993 | 0.7200305746 |
| C | 0.0000000000 | 0.0000000000 | 0.0000000000 |
| C | -1.2426390169 | 1.9435608748 | 0.4321791544 |
| C | -2.3033368875 | 1.6643674024 | -0.6292142446 |
| C | -2.2488884584 | 0.2172158661 | -1.0192124265 |
| C | -1.2023380600 | -0.5412748898 | -0.7181316991 |
| H | -1.9773460867 | 3.8523808238 | 1.0761039364 |
| H | -0.2784169671 | 3.5400673066 | 1.4840710481 |
| H | -0.7409518165 | 3.9382630677 | -0.1827556747 |
| H | -3.0846472616 | -0.1806599236 | -1.5861749840 |
| H | -1.1582404007 | -1.5915003080 | -0.9890626595 |
| H | -1.5528424434 | 1.4253311585 | 1.3538622798 |
| H | 0.0210958541 | -0.3642888597 | 1.0394763848 |
| H | 1.1910041858 | -0.1717902222 | -1.4894195664 |
| H | -3.2877627283 | 1.9011133341 | -0.2130622654 |
| H | -1.3208367733 | 2.2900121159 | -2.1662643359 |

Electronic Energy:

-459.739083424814

Correction to Gibbs Free Energy:

0.12688001

**6i**

| | | | |
|---|---|---|---|
| O | 0.0000000000 | 1.4171720046 | 0.0000000000 |
| O | -1.8993255838 | 1.8024197186 | 1.9559446346 |
| O | 0.0108785821 | -0.5365766776 | 1.2921001434 |
| C | -1.2497284982 | -0.3679631658 | -0.7405722881 |
| C | -1.9976775126 | 0.7108086234 | -0.9032924174 |
| C | -1.2737403381 | 1.9118285141 | -0.3901539158 |
| C | -1.9330279199 | 2.6041736543 | 0.8023640719 |
| C | 0.0000000000 | 0.0000000000 | 0.0000000000 |
| H | -1.1272726343 | 2.6543061866 | -1.1861109536 |
| H | -0.8863346890 | -0.4569184285 | 1.6399860846 |
| H | -0.9669694819 | 1.6677209491 | 2.1616078519 |
| H | 0.9342716061 | -0.3326704804 | -0.4578223716 |
| H | -1.4947999807 | -1.3892097062 | -0.9977035205 |
| H | -2.9913553707 | 0.7588175497 | -1.3290461457 |
| H | -2.9816286902 | 2.8158266443 | 0.5797712111 |
| H | -1.4260565224 | 3.5612275369 | 0.9770355210 |

Electronic Energy:

-420.483412552062

Correction to Gibbs Free Energy:

0.1005291

**4i**

| | | | |
|---|---|---|---|
| O | 0.0000000000 | 1.4082525613 | 0.0000000000 |
| O | 1.1786328742 | -0.4393456592 | -0.5818120909 |
| O | -2.2559045931 | 2.5080372931 | -1.6871997395 |
| C | 0.0000000000 | 0.0000000000 | 0.0000000000 |
| C | -1.2335587186 | 1.9307970424 | 0.4492750551 |
| C | -2.3313092176 | 1.6522042533 | -0.5602985839 |
| C | -2.2708470604 | 0.2121746237 | -0.9765804777 |
| C | -1.2128796386 | -0.5410798149 | -0.7027566606 |

| | | | |
|---|---|---|---|
| H | -3.1120684091 | -0.1840614246 | -1.5364616502 |
| H | -1.1651765848 | -1.5875685119 | -0.9873610102 |
| H | -1.0975892062 | 3.0061569405 | 0.5680160827 |
| H | -1.4987802169 | 1.4990042868 | 1.4256313355 |
| H | 0.0356787036 | -0.3637861499 | 1.0392773121 |
| H | 1.1673262956 | -0.1732119756 | -1.5075284973 |
| H | -3.3013758785 | 1.8681433416 | -0.1020041877 |
| H | -1.4035951282 | 2.3455248583 | -2.1043841999 |

Electronic Energy:

-420.482365163138

Correction to Gibbs Free Energy:

0.10077234

## Synthesis and analysis of products

**(*S*)-5-(2-hydroxypropan-2-yl)furan-2(5H)-one** ((*S*)-**7a**)

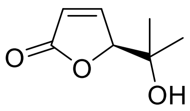

**Representative general procedure (semi-preparative)**: In a 50 ml Erlenmeyer flask, **1a** (10 mg, 70 µmol) was dissolved in phosphate buffer (10 ml, 50 mM, pH 7.5), KRED $P_2$-$G_{03}$ (15 mg) from *Codexis*, *evo*$_{030}$ (15 mg) from *evo Catal GmbH*, $NAD^+$ (6.6 mg, 10 µmol) and 2-propanol (2.6 mmol, 200 µl, 2% (v/v)) were added and the solution was incubated at 30 °C for 20 h. The reaction mixture was extracted with ethyl acetate (3 x 15 mL). The combined organic phases were dried over anhydrous $Na_2SO_4$ and concentrated under reduced pressure. The residue was purified by column chromatography ($SiO_2$, hexane/ethyl acetate, 1/1) yielding (*S*)-**7a** (8.5 mg, 60 µmol, 85%, 99% ee) as colorless oil.

**Preparative scale protocol**: In a 500 ml Erlenmeyer flask, **1a** (142 mg, 1.0 mmol) was dissolved in phosphate buffer (142 ml, 50 mM, pH 7.5), KRED $P_2$-$G_{03}$ (213 mg), *evo*$_{030}$ (213 mg), $NAD^+$ (93.7 mg, 141 µmol) and 2-propanol (2.84 ml, 2% (v/v)) were added and the solution was incubated at 30 °C for 20 h. The reaction mixture was extracted with ethyl acetate (3 x 100 mL). The combined organic phases were dried over anhydrous $Na_2SO_4$ and concentrated under reduced pressure. The residue was purified by column chromatography ($SiO_2$, hexane/ethyl acetate, 1/1) yielding (*S*)-**7a** (70.0 mg, 492 µmol, 49%) as colorless oil.

**$[\alpha]^{20}_D$**: –1.1 (c 0.5, $CHCl_3$). ***$R_f$*** = 0.22 (hexane/ethyl acetate 1/1). **$^1$H NMR** (400 MHz, $CDCl_3$) δ [ppm] = 7.52 (dd, *J* = 5.8 Hz, *J* = 1.5 Hz, 1H), 6.23 (dd, *J* = 8.8 Hz, *J* = 2.1 Hz, 1H), 4.90 (m, 1H), 1.31 (s, 1H), 1.43 (s, 6H). **$^{13}$C NMR** (100 MHz, $CDCl_3$) δ [ppm] = 173.0, 153.5, 123.0, 89.3, 71.7, 25.4. **FT-IR** (ATR): ν [cm$^{-1}$] = 3425 (s), 2955 (m), 1734 (s), 1168 (m), 1093 (m), 826 (m). **HPLC** (Chiralpak AD, hexane/isopropanol 90/10, 0.5 mL/min, 210 nm): $t_R$ ((*R*)-**7a**) = 21.6 min, $t_R$ ((*S*)-**7a**) = 27.4 min. **ESI-HRMS** $C_7H_{10}O_3$ (M+H) calc. 142.0630, found: 142.0701.

**(*S*)-5-(1-hydroxycyclohexyl)furan-2(5H)-one** ((*S*)-**7b**)

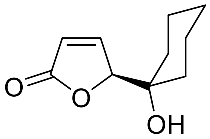

According to the general procedure, **1b** (10 mg, 55 µmol) was reacted in presence of KRED $P_2$-$G_{03}$ (15 mg), *evo*$_{030}$ (15 mg), $NAD^+$ (6.6 mg, 10 µmol) and 2-propanol (200 µl, 2% (v/v)). The residue was purified by column chromatography ($SiO_2$, hexane/ethyl acetate, 1/1) yielding (*S*)-**7b** (7.1 mg, 39 µmol, 71%, 99% ee) as colorless oil. **$[\alpha]^{20}_D$**: –0.95 (c 0.39, $CHCl_3$). ***$R_f$*** = 0.25 (hexane/ethyl acetate 1/1). **$^1$H NMR** (400 MHz, $CDCl_3$) δ [ppm] = 7.54 (dd, *J* = 5.8 Hz, *J* = 1.5 Hz, 1H), 6.21 (dd, *J* = 5.8 Hz, *J* = 2.1 Hz, 1H), 4.88 (m, 1H), 1.45-1.72 (m, 10 H). **$^{13}$C NMR** (100 MHz, $CDCl_3$) δ [ppm] = 173.4, 153.9, 123.2, 89.7, 73.0, 33.9, 33.8, 25.8, 21.5, 21.4. **FT-IR** (ATR): ν [cm$^{-1}$] = 3430 (s), 2955 (m), 1736 (s), 1168 (m), 1091

(m), 824 (m). **HPLC** (Chiralpak AD, hexane/isopropanol 90/10, 0.5 mL/min, 210 nm): $t_R$ ((*R*)-**7b**) = 27.5 min, $t_R$ ((*S*)-**7b**) = 37.9 min. **ESI-HRMS** $C_7H_{10}O_3$ (M+H) calc. 182.0943, found: 182.1016.

**(*S*)-5-(1-hydroxycyclopentyl)furan-2(5H)-one** ((*S*)-**7c**)

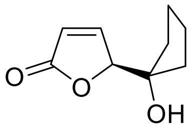

According to the general procedure, **1c** (10 mg, 59 µmol) was reacted in presence of KRED $P_2$-$G_{03}$ (15 mg), *evo*$_{030}$ (15 mg), $NAD^+$ (6.6 mg, 10 µmol) and 2-propanol (200 µl, 2% (v/v)). The residue was purified by column chromatography ($SiO_2$, hexane/ethyl acetate, 1/1) yielding (*S*)-**7c** (6.3 mg, 37 µmol, 63%, 97% ee) as colorless oil. **$[\alpha]^{20}_D$**: –1.0 (c 0,41, $CHCl_3$). ***$R_f$*** = 0.25 (hexane/ethyl acetate 1/1). **$^1$H NMR** (400 MHz, $CDCl_3$) δ [ppm] = 7.51 (dd, *J* = 5.7 Hz, *J* = 1.6 Hz, 1H), 6.24 (dd, *J* = 5.8 Hz, *J* = 2.1 Hz, 1H), 5.00 (dd, *J* = 2.1 Hz, *J* = 1.6 Hz, 1H), 1.86-1.92 (m, 2H), 1.68-1.74 (m, 6H). **$^{13}$C NMR** (100 MHz, $CDCl_3$) δ [ppm] = 173.5, 154.3, 123.4, 88.6, 82.4, 37.9, 36.9, 24.4, 24.3. **FT-IR** (ATR): ν [$cm^{-1}$] = 3423 (s), 2955 (m), 1734 (s), 1168 (m), 1093 (m), 826 (m). **HPLC** (Chiralpak AD, hexane/isopropanol 90/10, 0.5 mL/min, 210 nm): $t_R$ ((*R*)-**7c**) = 25.8 min, $t_R$ ((*S*)-**7c**) = 35.5 min. **ESI-HRMS** $C_7H_{10}O_3$ (M+H) calc. 168.0786, found: 168.0859.

**(*S*)-5-(1-hydroxycyclobutyl)furan-2(5H)-one** ((*S*)-**7d**)

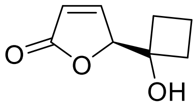

According to the general procedure, **1d** (10 mg, 65 µmol) was reacted in presence of KRED $P_2$-$G_{03}$ (15 mg), *evo*$_{030}$ (15 mg), $NAD^+$ (6.6 mg, 10 µmol) and 2-propanol (200 µl, 2% (v/v)). The residue was purified by column chromatography ($SiO_2$, hexane/ethyl acetate, 1/1) yielding (*S*)-**7d** (5.6 mg, 36 µmol, 56%, 92% ee) as colorless oil. **$[\alpha]^{20}_D$**: –0.95 (c 0,51, $CHCl_3$). ***$R_f$*** = 0.25 (hexane/ethyl acetate 1/1). **$^1$H NMR** (400 MHz, $CDCl_3$) δ [ppm] = 7.52 (dd, *J* = 5.8 Hz, *J* = 1.6 Hz, 1H), 6.27 (dd, *J* = 5.8 Hz, *J* = 2.1 Hz, 1H), 5.10 (m, 1H), 1.31 (s, 1H), 2.10-2.32 (m, 4H), 1.88-1.98 (m, 1H), 1.61-1.73 (m, 1H). **$^{13}$C NMR** (100 MHz, $CDCl_3$) δ [ppm] = 173.4, 153.2, 123.7, 87.1, 75.1, 32.7, 32.3, 12.4. **FT-IR** (ATR): ν [$cm^{-1}$] = 3429 (s), 2955 (m), 1737 (s), 1167 (m), 1091 (m), 829 (m). **HPLC** (Chiralpak AD, hexane/isopropanol 90/10, 0.5 mL/min, 210 nm): $t_R$ ((*R*)-**7d**) = 25.5 min, $t_R$ ((*S*)-**7d**) = 35.0 min. **ESI-HRMS** $C_7H_{10}O_3$ (M+H) calc. 154.0630, found: 154.0635.

**(*S*)-5-(1-hydroxycyclopropyl)furan-2(5H)-one** ((*S*)-**7e**)

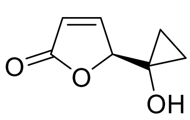

According to the general procedure, **1e** (20 mg, 143 µmol) in phosphate buffer (20 ml, 50 mM, pH 7.5) was reacted in presence of KRED $P_2$-$G_{03}$ (30 mg), *evo*$_{030}$ (30 mg), $NAD^+$ (13.2 mg, 20 µmol) and 2-propanol (400 µl, 2% (v/v)). The residue was purified by column chromatography ($SiO_2$, hexane/ethyl acetate, 1/1) yielding (*S*)-**7e** (5.0 mg, 36 µmol, 25%, 99% ee) as colorless oil. **$[\alpha]^{20}_D$**: –0.55 (c 0.2, $CHCl_3$). ***$R_f$*** = 0.25 (hexane/ethyl acetate 1/1). **$^1$H NMR** (400 MHz, $CDCl_3$) δ [ppm] = 7.50 (dd, *J* = 5.7 Hz, *J* = 1.6 Hz, 1H), 6.25 (dd, *J* = 5.8 Hz, *J* = 2.0 Hz, 1H), 4.77 (t, *J* = 1.8 Hz, 1H), 2.79 (bs, 1H), 0.95-1.05 (m, 2H), 0.81-0.86 (m, 1H), 0.72-0.77 (m, 1H). **$^{13}$C NMR** (100 MHz, $CDCl_3$) δ [ppm] = 173.4, 154.0, 123.5, 88.0, 55.1, 13.1, 10.9. **FT-IR** (ATR): ν [cm$^{-1}$] = 3429 (s), 2955 (m), 1732 (s), 1167 (m), 1091 (m), 830 (m). **HPLC** (Chiralpak AD, hexane/isopropanol 90/10, 0.5 mL/min, 210 nm): $t_R$ ((*R*)-**7e**) = 22.4 min, $t_R$ ((*S*)-**7e**) = 28.5 min. **ESI-HRMS** $C_7H_{10}O_3$ (M+H) calc. 140.0473, found: 140.0433.

**(*S*)-5-(3-hydroxypentan-3-yl)furan-2(5H)-one** ((*S*)-**7f**)

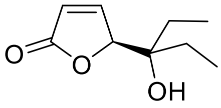

According to the general procedure, **1f** (20 mg, 118 µmol) in phosphate buffer (20 ml, 50 mM, pH 7.5) was reacted in presence of KRED $P_2$-$B_{02}$ (20 mg), $NADP^+$ (13.2 mg, 20 µmol) and 2-propanol (400 µl, 2% (v/v)). The residue was purified by column chromatography ($SiO_2$, hexane/ethyl acetate, 1/1) yielding (*S*)-**7f** (7.6 mg, 45 µmol, 38%, 88% ee) as colorless oil. **$[\alpha]^{20}_D$**: –0.8 (c 0.28, $CHCl_3$). ***$R_f$*** = 0.29 (hexane/ethyl acetate 1/1). **$^1$H NMR** (400 MHz, $CDCl_3$) δ [ppm] = 7.52 (dd, *J* = 5.8 Hz, *J* = 1.6 Hz, 1H), 6.17 (dd, *J* = 5.8 Hz, *J* = 2.1 Hz, 1H), 5.01 (dd, *J* = 2.1 Hz, *J* = 1.6 Hz, 1H), 1.52-1.72 (m, 4H), 0.92-0.98 (m, 6H). **$^{13}$C NMR** (100 MHz, $CDCl_3$) δ [ppm] = 173.1, 153.9, 122.7, 87.0, 75.5, 28.1, 27.6, 7.7, 7.3. **FT-IR** (ATR): ν [cm$^{-1}$] = 3429 (s), 2955 (m), 1737 (s), 1167 (m), 1091 (m), 823 (m). **HPLC** (Chiralpak AD, hexane/isopropanol 90/10, 0.5 mL/min, 210 nm): $t_R$ ((*R*)-**7f**) = 21.5 min, $t_R$ ((*S*)-**7f**) = 26.0 min. **ESI-HRMS** $C_7H_{10}O_3$ (M+H) calc. 170.0943, found: 170.0899.

**(*S*)-3-(hydroxymethyl)isobenzofuran-1(3H)-one** ((*S*)-**7g**)

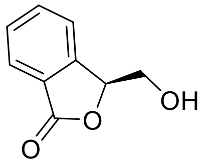

According to the general procedure, **1g** (20 mg, 122 µmol) in phosphate buffer (20 ml, 50 mM, pH 7.5) was reacted in presence of KRED $P_2$-$B_{02}$ (20 mg), $NADP^+$ (13.2 mg, 20 µmol) and 2-propanol (400 µl,

2% (v/v)). The residue was purified by column chromatography ($SiO_2$, hexane/ethyl acetate, 1/1) yielding (*S*)-**7g** (3.8 mg, 23 μmol, 19%, 94% ee) as colorless oil. **$^1$H NMR** (400 MHz, $CDCl_3$): δ [ppm] = 7.92 (d, *J* = 7.4 Hz, 1H), 7.70 (dt, *J* = 7.5 Hz, *J* = 1.1 Hz, 1H), 7.59 (t, *J* = 7.5 Hz, 1H), 7.53 (d, *J* = 7.6 Hz, 1H), 5.56 (dd, *J* = 5.7 Hz, *J* = 3.6 Hz, 1H), 4.14 (dd, *J* = 12.4 Hz, *J* = 3.6 Hz, 1H), 3.91 (dd, *J* = 12.4 Hz, *J* = 5.7 Hz, 1H), 1.94 (bs, 1H). **$^{13}$C NMR** (100 MHz, $CDCl_3$) δ [ppm] = 146.5, 134.3, 129.6, 125.9, 122.1, 81.7, 64.1. **GC** (50 °C (5 min)/5 °C min$^{-1}$ / 110 °C (10 min) /5 °C min$^{-1}$ /10 °C min$^{-1}$ / 180°C (6 min) / 10 °C min$^{-1}$ /200 (20 min)): $t_R$ ((*S*)-**7g**) = 49.2 min, $t_R$ ((*R*)-**7g**) = 49.8 min.

**(*S*)-5-((S)-1-hydroxyethyl)furan-2(5H)-one** ((*S,S*)-**7h**)

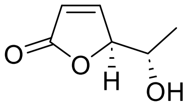

According to the general procedure, (*S*)-**1h** (20 mg, 156 μmol) in phosphate buffer (20 ml, 50 mM, pH 7.5) was reacted in presence of KRED $P_2$-$B_{02}$ (20 mg), *evo* 1.1. 30 (20 mg), $NADP^+$ (13.2 mg, 20 μmol) and 2-propanol (400 μl, 2% (v/v)). The residue was purified by column chromatography ($SiO_2$, hexane/ ethyl acetate, 1/1) yielding (*S,S*)-**7h** (3.4 mg, 27 μmol, 17%, 98% de) as colorless oil. **$[\alpha]^{20}_D$**: –11.3 (c 0,33, $CHCl_3$). ***$R_f$*** = 0.16 (hexane/ethyl acetate 1/1). **$^1$H NMR** (400 MHz, $CDCl_3$): δ [ppm] = 7.44 (dd, *J* = 5.8 Hz, *J* = 1.6 Hz, 1H), 6.20 (dd, *J* = 5.8 Hz, *J* = 2.0 Hz, 1H), 4.92 (ddd, *J* = 5.5 Hz, *J* = 2.0 Hz, *J* = 1.6 Hz, 1H), 3.92 (quint, *J* = 6.1 Hz, 1H), 2.14 (br, 1H), 1.32 (d, *J* = 6.5 Hz, 1H). **$^{13}$C NMR** (100 MHz, $CDCl_3$): δ [ppm] = 172.7, 153.3, 123.1, 87.2, 68.5, 18.9. **FT-IR** (ATR): ν [cm$^{-1}$] = 3429 (s), 2955 (m), 1734 (s), 1167 (m), 1091 (m), 827 (m). **HPLC** (Chiralpak AD, hexane/isopropanol 95/5, 0.5 mL/min, 210 nm): $t_R$ ((*R,R*)-**7h**) = 30.3 min, $t_R$ ((*S,S*)-**7h**) = 41.1 min. **ESI-HRMS** $C_6H_8O_3$ (M+H) calc. 128.0473, found: 128.0556.

**(*S*,*S*)-5-(2-hydroxy-4-(2,6,6-trimethylcyclohex-1-en-1-yl)butan-2-yl)furan-2(5H)-one** ((*S*,*S*)-**7k**)

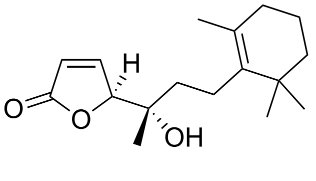

According to the general procedure, **1k** (10 mg, 36 μmol) in phosphate buffer (10 ml, 50 mM, pH 7.5) was reacted in presence of KRED $P_2$-$B_{02}$ (10 mg), $NAD^+$ (8.0 mg, 12 μmol) and 2-propanol (200 μl, 2% (v/v)). The crude mixture obtained from the biotransformation was flushed through a silica plug ($SiO_2$, cyclohexane/ethyl acetate, 9/1), and the resulting material was dissolved in MeOH (0.5 mL) and treated with 1M NaOH (1 mL) under stirring at room temperature for 45 min. The reaction mixture was then acidified with 1M HCl (1.2 mL), and the resulting aqueous phase was extracted with ethyl acetate (3 × 5 mL). The combined organic layers were dried over anhydrous $Na_2SO_4$ and concentrated under reduced pressure. Purification by column chromatography ($SiO_2$, cyclohexane/ethyl acetate, 9/1) afforded **7k** (1.1 mg, 4 μmol, 11 % yield, 29 % ee, >99 de) as a colorless oil. ***$R_f$*** = 0.19 (cyclohexane/ethyl

acetate 1/1). **$^1$H NMR** (400 MHz, $CDCl_3$) δ [ppm] = 7.47 (dd, *J* = 5.8, 1.5 Hz, 1H), 6.20 (dd, *J* = 5.8 Hz, *J* = 2.1 Hz, 1H), 4.96 (t, *J* = 1.8 Hz, 1H), 2.4 (m, 2H), 1.89 (m, 4H), 1.64 (m, 2H), 1.60 (s, 3H), 1.42 (m, 2H), 1.25 (s, 3H), 0.99 (s, 6H); **$^{13}$C NMR** (100 MHz, $CDCl_3$) δ [ppm] = 162.7, 144.7, 135.9, 127.9, 122.1, 90.5, 85.2, 67.8, 39.8, 35.1, 32.8, 28.7, 28.6, 22.9, 21.8, 19.8, 19.5; **HPLC** (Chiralpak AD, hexane/isopropanol 90/10, 0.5 mL/min, 210 nm): $t_R$ ((*S,R*)-**7k**) = 12.6 min, $t_R$ ((*S,S*)-**7k**) = 13.9 min, $t_R$ ((*R,S*)-**7k**) = 17.7 min, $t_R$ ((*S,S*)-**7k**) = 18.2 min; **ESI-HRMS** $C_{17}H_{27}O_3^+$ $[M+H]^+$ theoretical 278.1882, found 278.1922.

**(*S*)-5-(2-hydroxypropan-2-yl)dihydrofuran-2(3H)-one** ((*S*)-**15**)

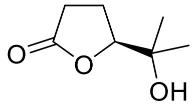

In a 50 ml Erlenmeyer flask, 2-(furan-2-yl)propan-2-ol (20 mg, 159 µmol) was dissolved in citrate buffer (20 ml, 50 mM, pH 6.0), glucose oxidase (30 U), chloroperoxidase (300 U) and glucose (43 mg, 239 µmol) were added and the solution was incubated at 30 °C. After full conversion was detected by GC, the reaction mixture was adjusted to pH 7.5 using phosphate buffer (1M, pH 8.0). KRED $P_2$-$G_{03}$ (15 mg), *evo*$_{030}$ (15 mg), $NAD^+$ (13.2 mg, 20 µmol) and 2-propanol (400 µl, 2% (v/v)) were added and the solution was incubated at 30 °C for 20 h. ERED-207 from *Codexis* (10 mg), Glucose dehydrogenase from *Sigma* (30 U) and glucose (43 mg, 239 µmol) were added and the solution was then incubated at 30 °C for 20 h. The reaction mixture was extracted with ethyl acetate (3 x 15 mL), The combined organic layers were dried over anhydrous $Na_2SO_4$ and concentrated under reduced pressure. The residue was purified by column chromatography ($SiO_2$, hexane/ethyl acetate, 1/1) yielding (*S*)-**15** (12 mg, 84 µmol, 53%) as colorless oil. **$^1$H NMR** (400 MHz, $CDCl_3$) δ [ppm] = 4.31 (t, *J* = 7.6 Hz, 1H), 2.50-2.64 (m, 2H), 2.12-2.27 (m, 2H), 1.78 (bs, 1H), 1.33 (s,1H), 1.19 (s,1H). **$^{13}$C NMR** (100 MHz, $CDCl_3$) δ [ppm] = 177.2, 86.2, 71.0, 29.0, 26.7, 24.0, 22.3. **FT-IR** (ATR): ν [$cm^{-1}$] = 3428 (s), 2955 (m), 1734 (s), 1168 (m), 1091 (m). **ESI-HRMS** $C_7H_{12}O_3$ (M+H) calc. 144.0786, found: 144.0859.

**Preparation of substrates**

Substrates **1a-1i** were prepared according to the known procedures in our previous works and the spectra were identical to the literature.[1]

**(*E*)-6-hydroxy-2-(prop-1-en-1-yl)-2H-pyran-3(6H)-one** ((*rac*)-**1j**)

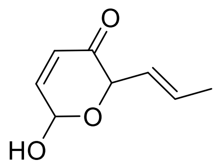

To a solution of furan (680 mg, 10.0 mmol) in anhydrous THF (20 mL) was added n-BuLi (2.5M, 1.5 equiv., 15.0 mmol) at –78 °C under argon, and the mixture was stirred for 4 h, allowing to warm up to 0 °C. Crotonaldehyde (1.5 equiv., 15.0 mmol) was added at –78 °C, and the reaction mixture was stirred for 10 h at 0 °C. The reaction was quenched with saturated aqueous $NH_4Cl$ solution (10 mL), extracted with diethyl ether (3 x 15 mL), dried over anhydrous $Na_2SO_4$, and concentrated under reduced pressure. The residue was purified by column chromatography ($SiO_2$, pentane/diethyl ether = 5/1) to give (*E*)-1-(furan-2-yl) but-2-en-1-ol as yellow oil (1.10 g, 8.0 mmol, 80%). **$^1$H NMR** (400 MHz, $CDCl_3$): $\delta$ [ppm] = 7.39 (dd, *J* = 1.6, 0.8 Hz, 1H), 6.33 (dd, J = 3.2, 1.6 Hz, 1H), 6.24 (d, *J* = 3.2 Hz, 1H), 5.74 -5.89 (m, 2H), 5.16 (t, J = 5.0, 1H), 2.05 (br, 1H) 1.76 (d, *J* = 5.8, 3H). **$^{13}$C NMR** (100 MHz, $CDCl_3$): $\delta$ [ppm] = 156.0, 142.4, 130.1, 129.1, 110.3, 106.4, 68.8, 17.8. To a solution of (*E*)-1-(furan-2-yl) but-2-en-1-ol (690 mg, 5.0 mmol) in THF/$H_2O$ (3/1, 12 mL), $NaHCO_3$ (420 mg, 2.0 equiv., 10.0 mmol), NaOAc·3$H_2O$ (410 mg, 1.0 equiv., 5.0 mmol), and NBS (890, 1.0 equiv., 5.0 mmol) were added at 0 °C. The reaction mixture was stirred for 1 h at 0 °C, then quenched with saturated aqueous $NaHCO_3$ (10 mL). The reaction mixture was extracted with ethyl acetate (3 x 15mL), dried over anhydrous $Na_2SO_4$, and concentrated under reduced pressure. The residue was purified by flash column chromatography ($SiO_2$, hexane/ethyl acetate, 3/1) to yield the (*rac*)-**1j** (561mg, 3.65 mmol, 73%) as colorless oil. **$^1$H NMR** (400 MHz, $CDCl_3$): $\delta$ [ppm] = 6.90 (dd, *J* = 10.2, J = 3.2, 1H), 6.14 (d, *J* = 10.1, 1H), 5.86-5.89 (m, 1H), 5.96 (m, 1H), 5.58-5.65 (m, 1H), 4.99 (d, *J* = 6.7, 1H), 3.06 (br, 1H), 1.78 (dt, *J* = 6.5, 3H). **$^{13}$C NMR** (100 MHz, $CDCl_3$): $\delta$ [ppm] = 184.2, 144.6, 132.5, 127.8, 124.2, 88.0, 75.4, 18.1.

**2-(furan-2-yl)-4-(2,6,6-trimethylcyclohex-1-en-1-yl)butan-2-ol** (*rac*-**5k**)

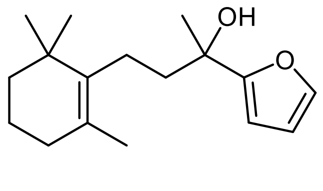

To a solution of furan (408 mg, 6.0 mmol) in anhydrous THF (15 mL) was added *n*-BuLi (2.5 M, 1.1 equiv., 5.5 mmol) dropwise at –78 °C under argon, and the mixture was stirred for 1 h, while gradually warming to 0 °C. Dihydro-β-ionone (972 mg, 5.0 mmol) was added at –78 °C, and the reaction mixture was stirred for 4 h at 0 °C. The reaction was quenched with saturated aqueous $NH_4Cl$ solution (5 mL), extracted with ethyl acetate (3 x 10 mL), dried over $Na_2SO_4$, and concentrated under reduced pressure.

The residue was purified by column chromatography ($SiO_2$, cyclohexane/ethyl acetate, 20/1) to give the title compound as yellow oil (1.04 g, 3.97 mmol, 79 %). ***R<sub>f</sub>*** = 0.55 (cyclohexane/ethyl acetate 4:1). **$^1$H NMR** (400 MHz, $CDCl_3$) δ [ppm] = 7.35 (dd, *J* = 1.8, 0.8 Hz, 1H), 6.31 (dd, *J* = 3.2, 1.8 Hz, 1H), 6.21 (dd, *J* = 3.2, 0.8 Hz, 1H), 1.92 (m, 8H), 1.57 (s, 3H), 1.52 (s, 3H), 1.39 (m, 2H), 0.94 (s, 3H), 0.93 (s, 3H); **$^{13}$C NMR** (400 MHz, $CDCl_3$) δ [ppm] = 159.5, 141.4, 136.3, 127.3, 110.0, 104.6, 71.8, 41.9, 39.8, 35.0, 32.8, 28.6, 28.5, 26.2, 22.9, 19.6, 19.5.

**6-hydroxy-2-methyl-2-(2-(2,6,6-trimethylcyclohex-1-en-1-yl)ethyl)-2H-pyran-3(6H)-one** (*rac*-**1k**)

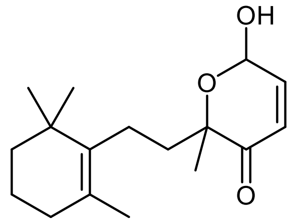


In a 25 ml round-bottom flask, a solution of vanadyl acetylacetonate (10 mg, 0.038 mmol) in dry dichloromethane (1 mL) was added dropwise to a solution of 2-(furan-2-yl)-4-(2,6,6-trimethylcyclohex-1-en-1-yl)butan-2-ol (0.50 g, 1.9 mmol) and *tert*-butyl hydroperoxide (0.23 mL, 2.38 mmol) in dry dichloromethane (5 mL), and the mixture was stirred at room temperature under argon for 3 h. The reaction was quenched with saturated aqueous $Na_2SO_4$, the layers were separated and the organic layer was concentrated *in vacuo* to afford the crude product as a yellow oil. Purification by column chromatography ($SiO_2$, cyclohexane/ethyl acetate, 5/1) yielded *rac*-**1k** as a colorless oil (307 mg, 1.10 mmol, 58 %) in a mixture of diastereoisomers (dr = 56/44). ***R<sub>f</sub>*** = 0.31 (cyclohexane/ethyl acetate 4/1). **$^1$H-NMR** (400 MHz, $CDCl_3$) [major diastereoisomer] δ [ppm] = 6.88 (dd, *J* = 10.3, 2.7 Hz, 1H), 6.09 (dd, *J* = 10.3, 1.0 Hz, 1H), 5.75 (ddd, *J* = 6.2, 2.6, 1.1Hz, 1H), 3.05 (d, *J* = 6.1 Hz, 1H), 2.03 (m, 5H), 1.62 (m, 8H), 1.39 (m, 3H), 0.97 (s, 3H), 0.95 (s, 3H); [minor diastereoisomer] δ [ppm] = 6.86 (dd, *J* = 10.3, 1.8 Hz, 1H), 6.08 (dd, *J* = 10.3, 1.3 Hz, 1H), 5.71 (dt, *J* = 7.5, 1.6 Hz, 1H), 3.01 (d, *J* = 7.5 Hz, 1H), 2.03 (m, 5H), 1.62 (m, 8H), 1.39 (m, 3H), 0.98 (s, 3H), 0.95 (s, 3H); **$^{13}$C-NMR** (400 MHz, $CDCl_3$) [major diastereoisomer] δ [ppm] = 199.0, 144.9, 136.2, 127.7, 126.8, 87.9, 82.2, 39.8, 38.0, 35.0, 32.7, 28.6, 25.6, 22.3, 21.6, 19.8, 19.5; [minor diastereoisomer] δ [ppm] = 198.8, 146.2, 136.3, 127.6, 127.3, 87.9, 82.0, 39.9, 38.9, 35.1, 32.8, 28.6, 25.6, 22.2, 21.6, 19.7, 19.5.

## NMR spectra of the products

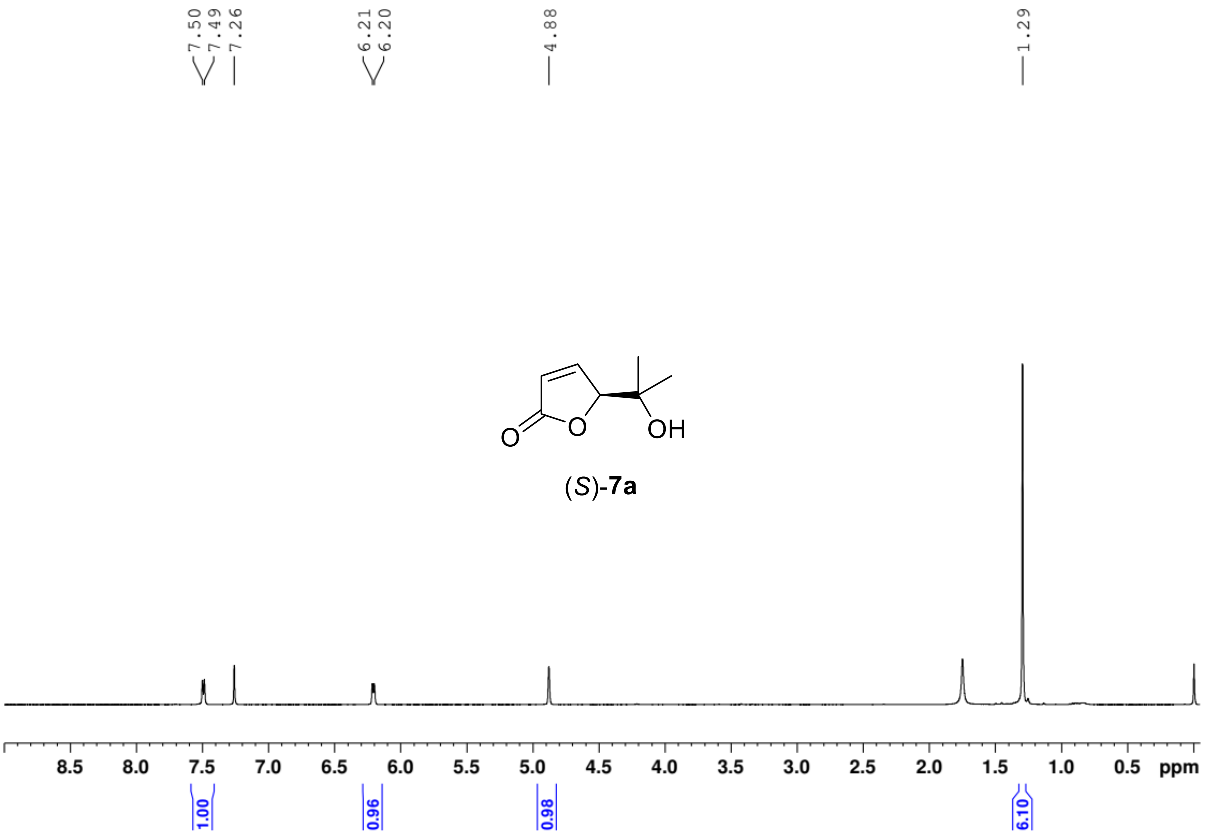


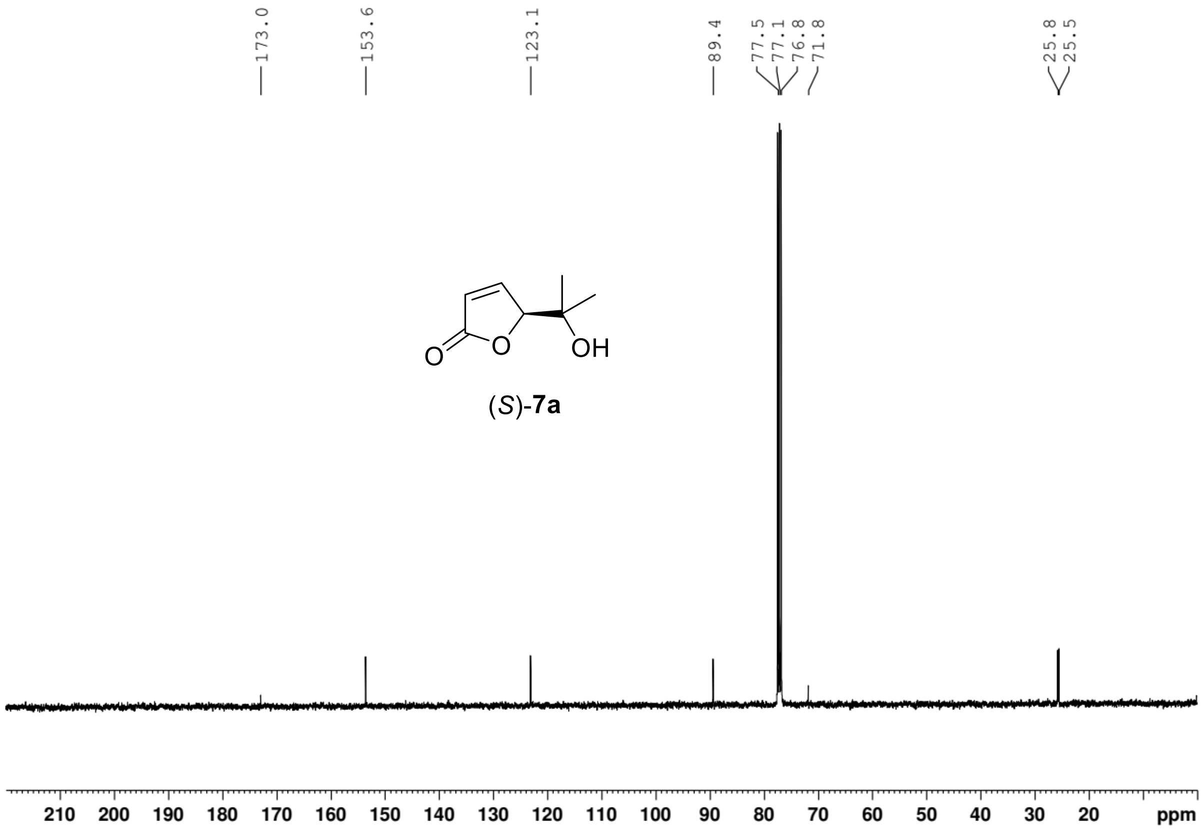

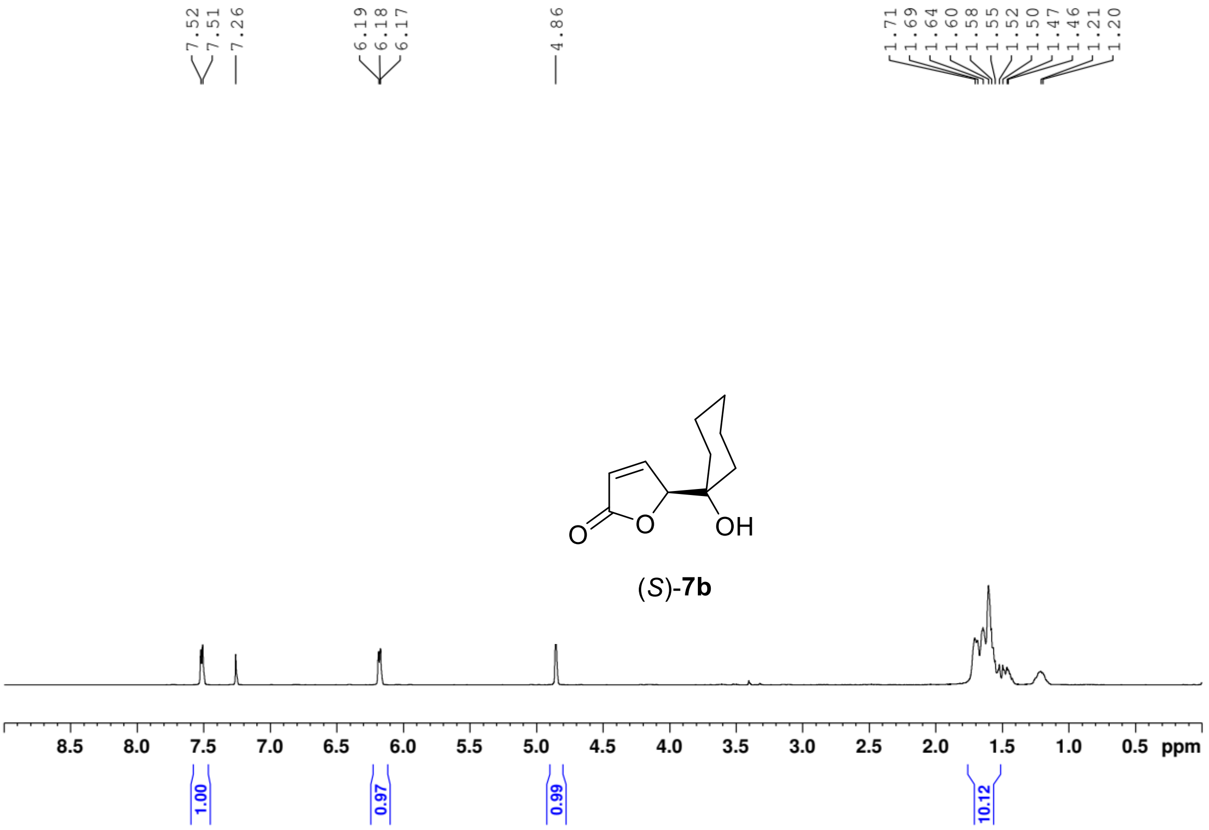
7.52
7.51
7.26
6.19
6.18
6.17
4.86
1.71
1.69
1.64
1.60
1.58
1.55
1.52
1.50
1.47
1.46
1.21
1.20
OH
O
O
(S)-7b
8.5 8.0 7.5 7.0 6.5 6.0 5.5 5.0 4.5 4.0 3.5 3.0 2.5 2.0 1.5 1.0 0.5 ppm
1.00
0.97
0.99
10.12

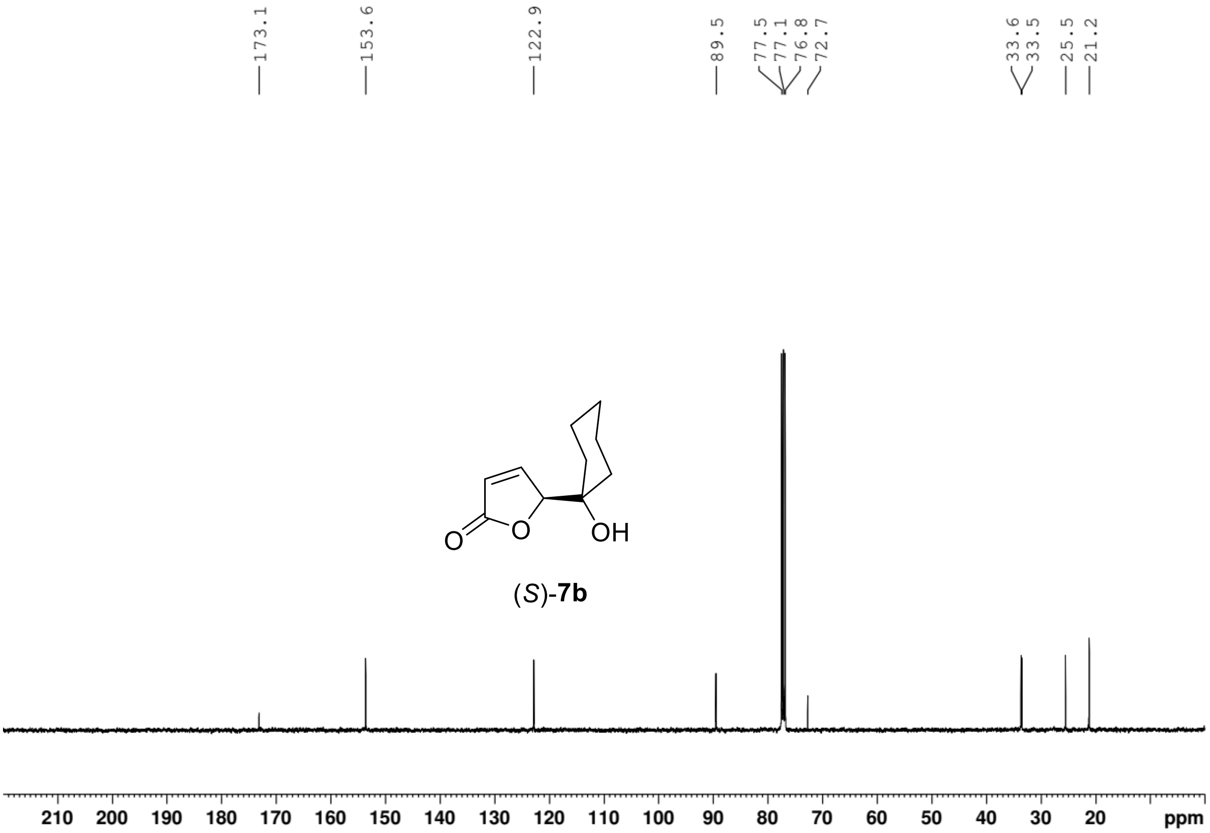
173.1
153.6
122.9
89.5
77.5
77.1
76.8
72.7
33.6
33.5
25.5
21.2
OH
O
O
(S)-7b
210 200 190 180 170 160 150 140 130 120 110 100 90 80 70 60 50 40 30 20 ppm

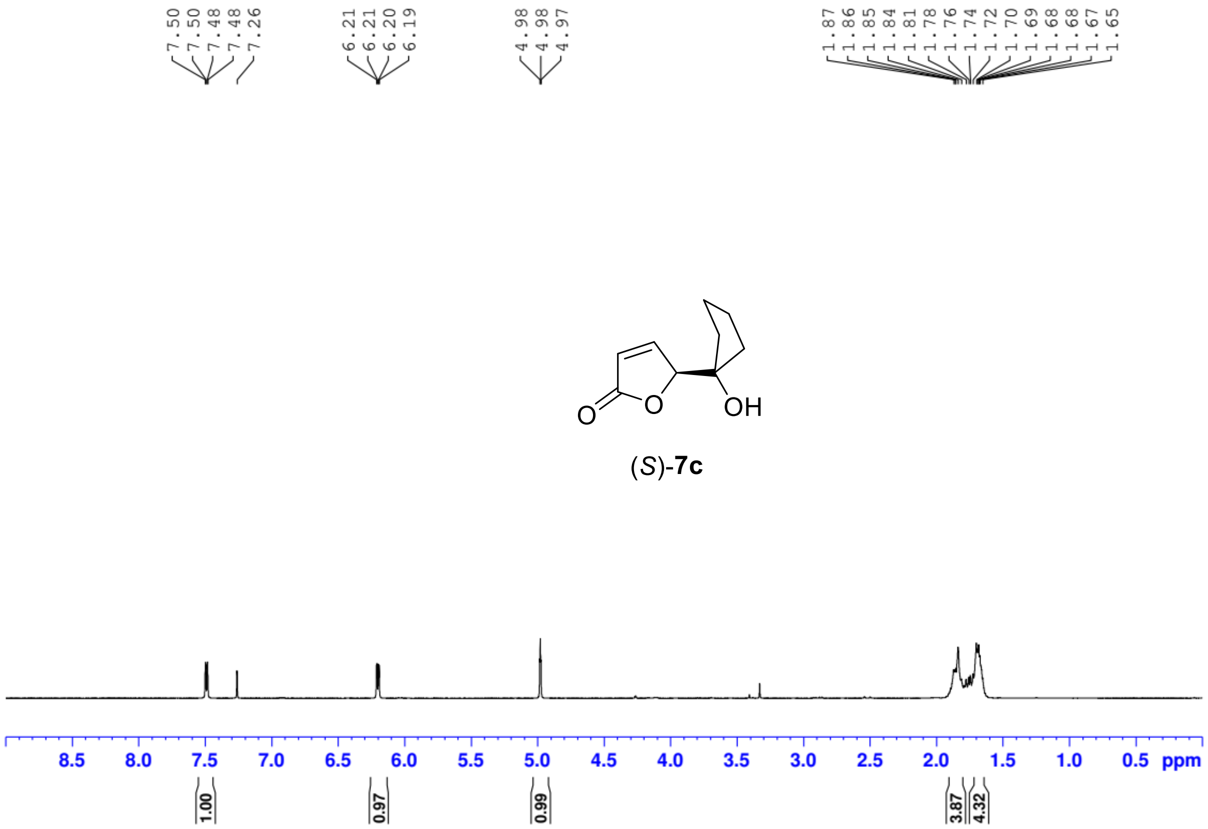
7.50
7.50
7.48
7.48
7.26
6.21
6.21
6.20
6.19
4.98
4.98
4.97
1.87
1.86
1.85
1.84
1.81
1.78
1.76
1.74
1.72
1.70
1.69
1.68
1.68
1.67
1.65
O
O
OH
(S)-7c
8.5 8.0 7.5 7.0 6.5 6.0 5.5 5.0 4.5 4.0 3.5 3.0 2.5 2.0 1.5 1.0 0.5 ppm
1.00
0.97
0.99
3.87
4.32

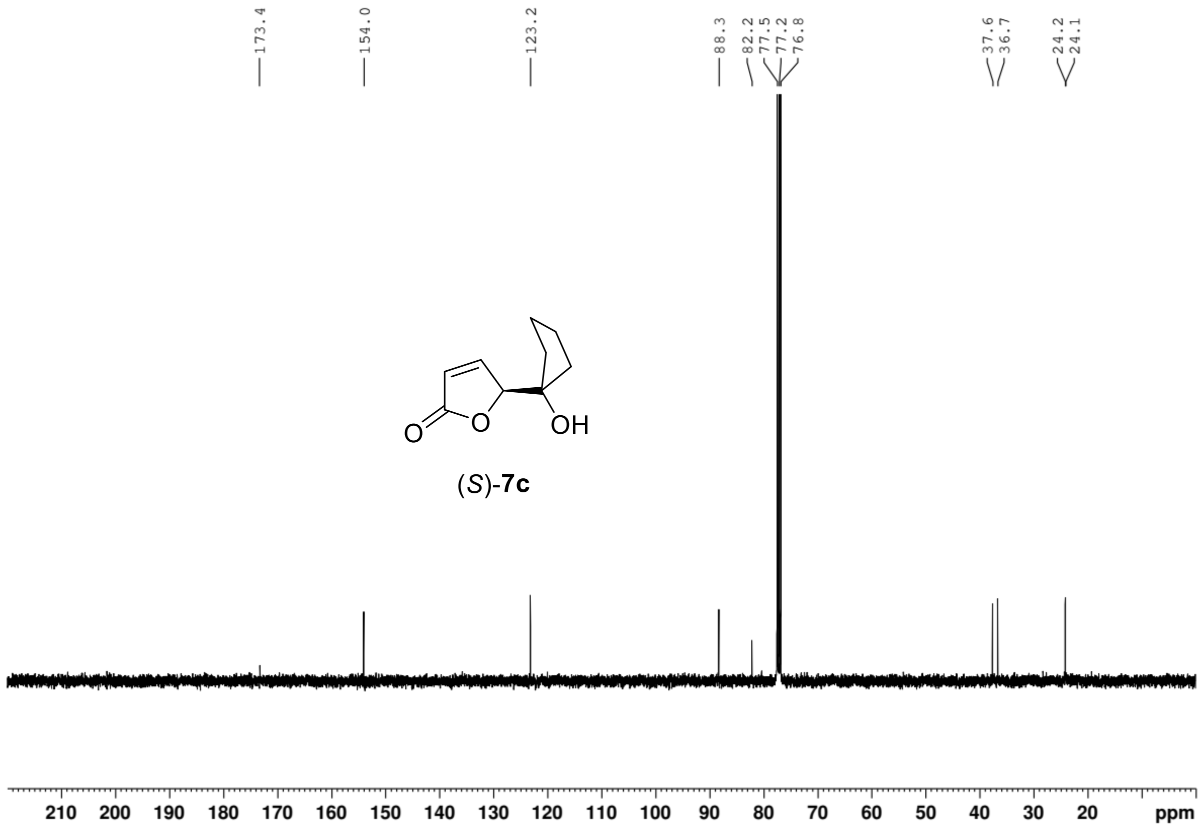
173.4
154.0
123.2
88.3
82.2
77.5
77.2
76.8
37.6
36.7
24.2
24.1
O
O
OH
(S)-7c
210 200 190 180 170 160 150 140 130 120 110 100 90 80 70 60 50 40 30 20 ppm

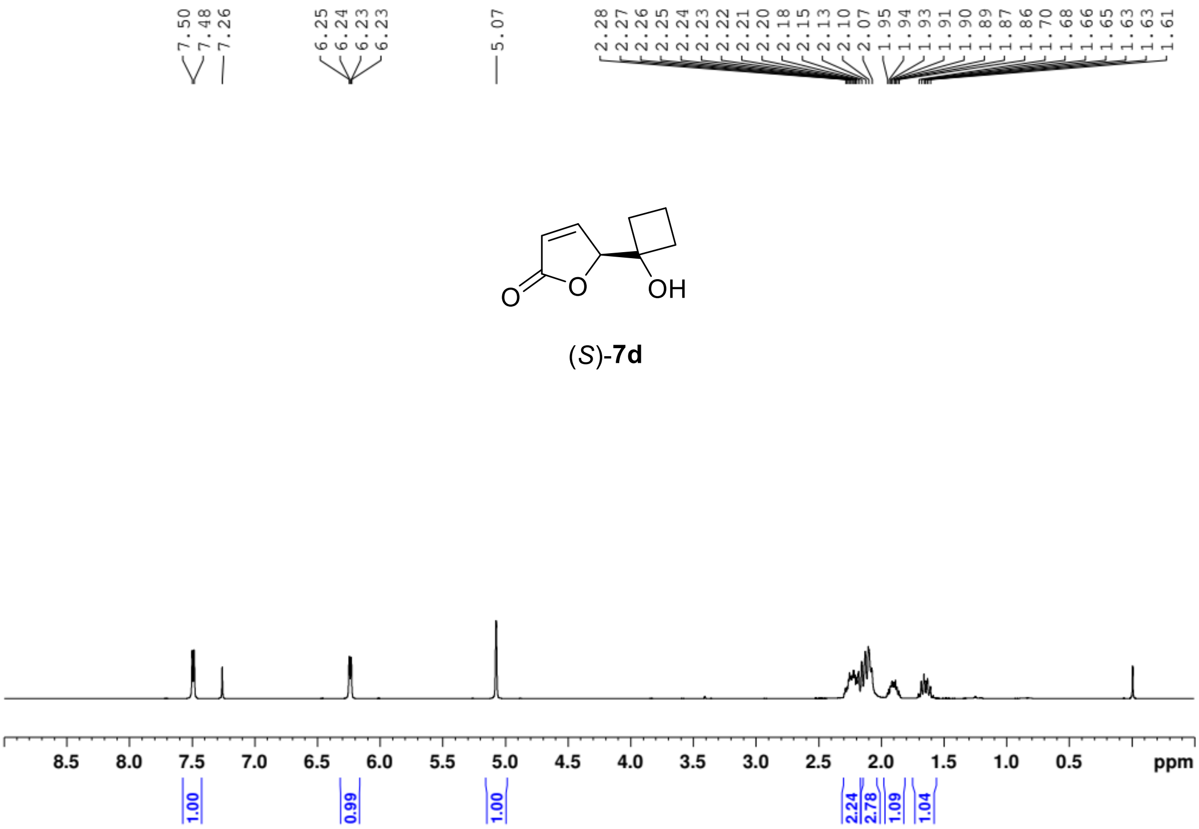
7.50
7.48
7.26
6.25
6.24
6.23
6.23
5.07
2.28
2.27
2.26
2.25
2.24
2.23
2.22
2.21
2.20
2.18
2.15
2.13
2.10
2.07
1.95
1.94
1.93
1.91
1.90
1.89
1.87
1.86
1.70
1.68
1.66
1.65
1.63
1.63
1.61
O
O
OH
(S)-7d
8.5 8.0 7.5 7.0 6.5 6.0 5.5 5.0 4.5 4.0 3.5 3.0 2.5 2.0 1.5 1.0 0.5 ppm
1.00
0.99
1.00
2.24
2.78
1.09
1.04

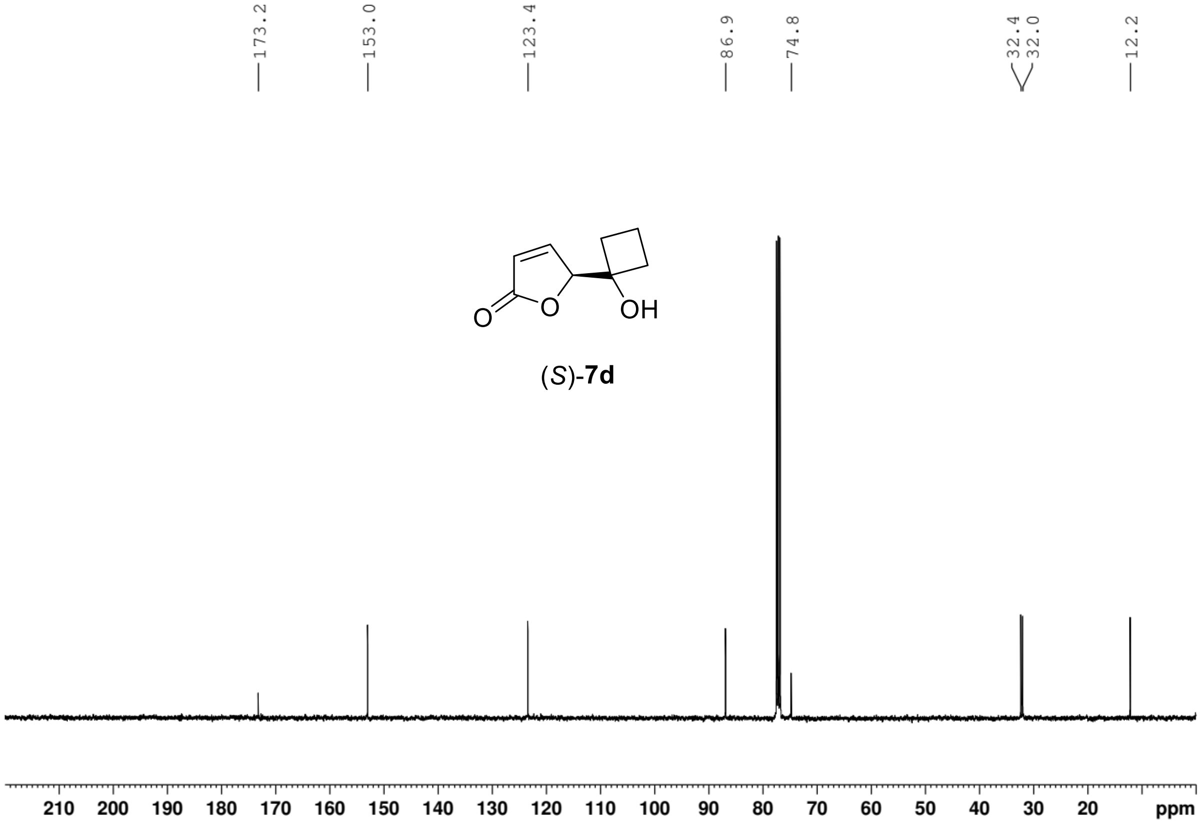
173.2
153.0
123.4
86.9
74.8
32.4
32.0
12.2
O
O
OH
(S)-7d
210 200 190 180 170 160 150 140 130 120 110 100 90 80 70 60 50 40 30 20 ppm

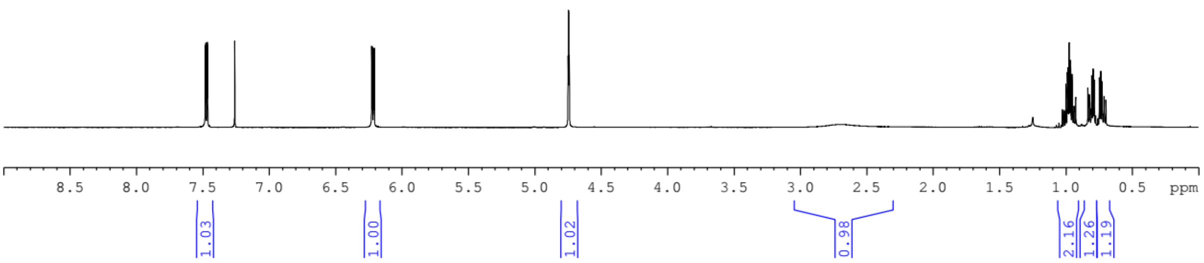
7.48
7.48
7.47
7.46
7.26
6.23
6.22
6.21
6.21
4.75
4.74
4.74
2.72
2.72
2.71
2.71
2.70
2.70
2.69
2.68
2.68
1.03
1.01
1.01
1.00
0.99
0.98
0.98
0.98
0.97
0.96
0.95
0.94
0.94
0.93
0.83
0.82
0.82
0.81
8.5 8.0 7.5 7.0 6.5 6.0 5.5 5.0 4.5 4.0 3.5 3.0 2.5 2.0 1.5 1.0 0.5 ppm
1.03
1.00
1.02
0.98
2.16
1.26
1.19

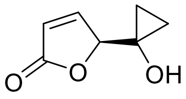
O
O
OH

(*S*)-**7e**

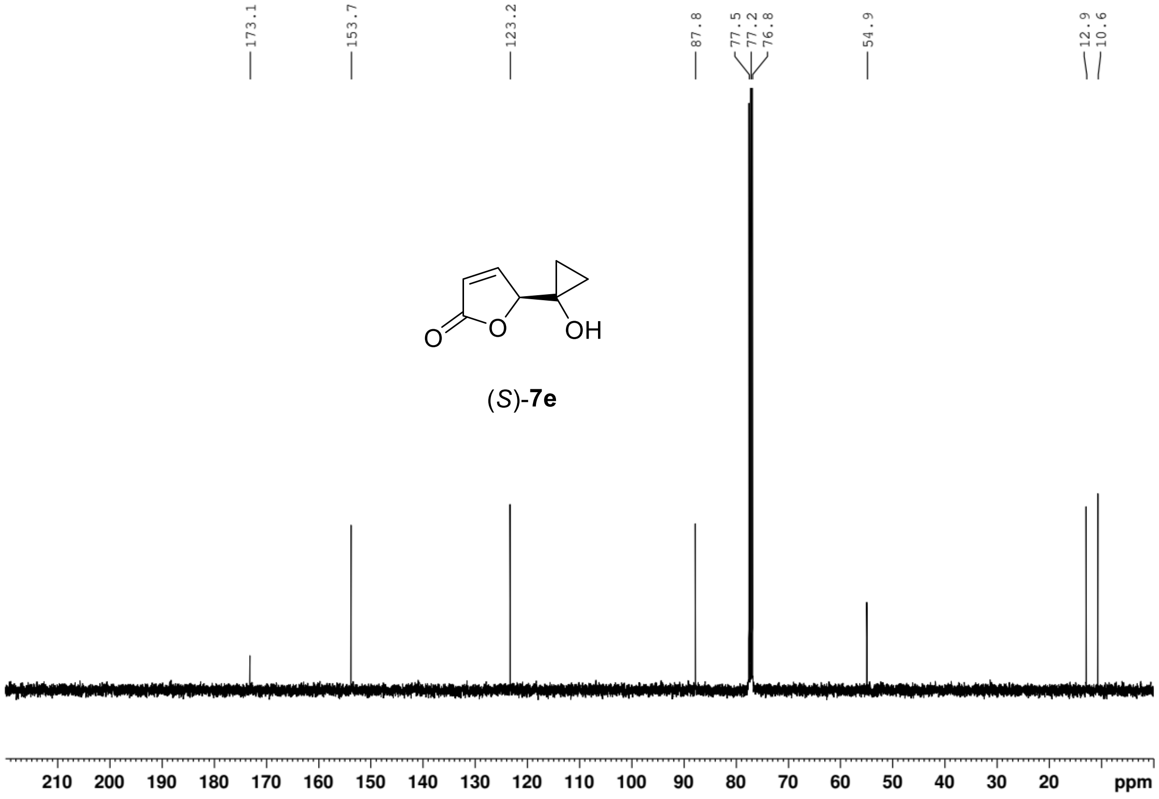
173.1
153.7
123.2
87.8
77.5
77.2
76.8
54.9
12.9
10.6
O
O
OH
(S)-7e
210 200 190 180 170 160 150 140 130 120 110 100 90 80 70 60 50 40 30 20 ppm

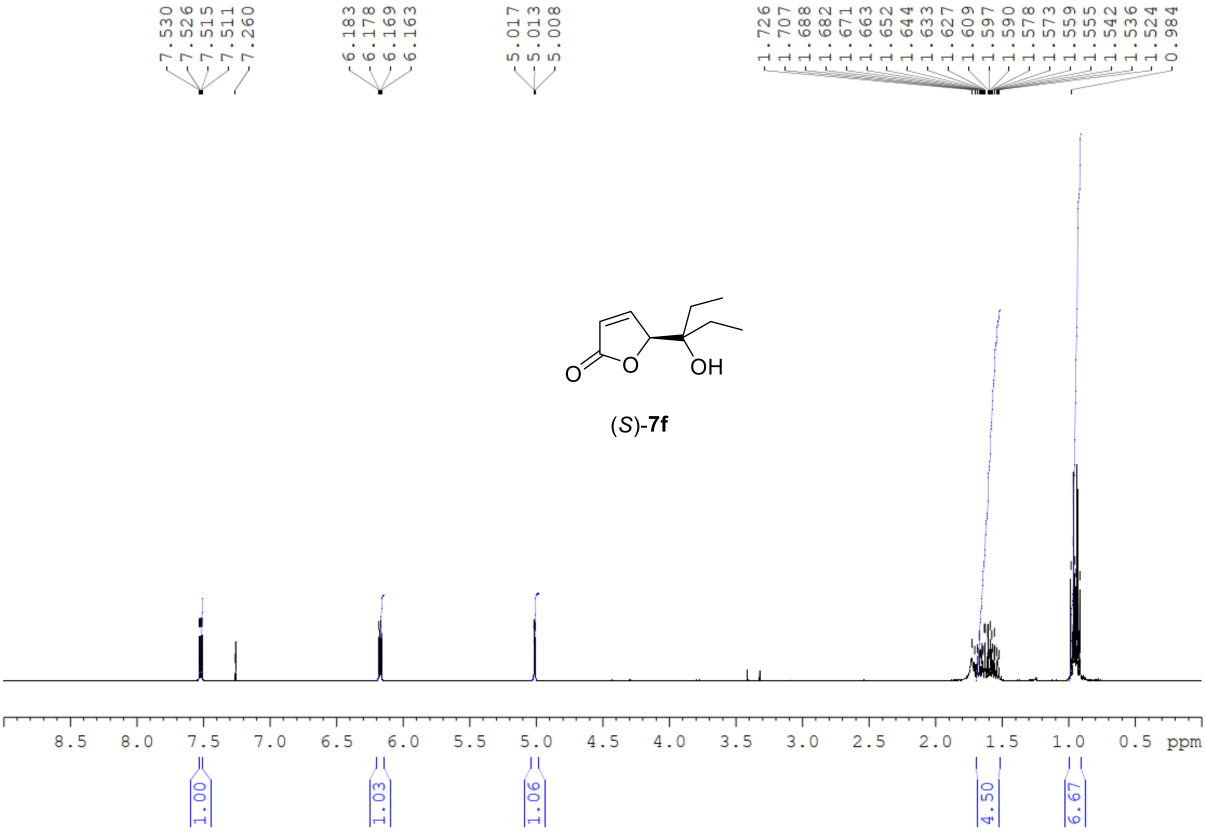
7.530
7.526
7.515
7.511
7.260
6.183
6.178
6.169
6.163
5.017
5.013
5.008
1.726
1.707
1.688
1.682
1.671
1.663
1.652
1.644
1.633
1.627
1.609
1.597
1.590
1.578
1.573
1.559
1.555
1.542
1.536
1.524
0.984
O
O
OH
(S)-7f
8.5 8.0 7.5 7.0 6.5 6.0 5.5 5.0 4.5 4.0 3.5 3.0 2.5 2.0 1.5 1.0 0.5 ppm
1.00
1.03
1.06
4.50
6.67

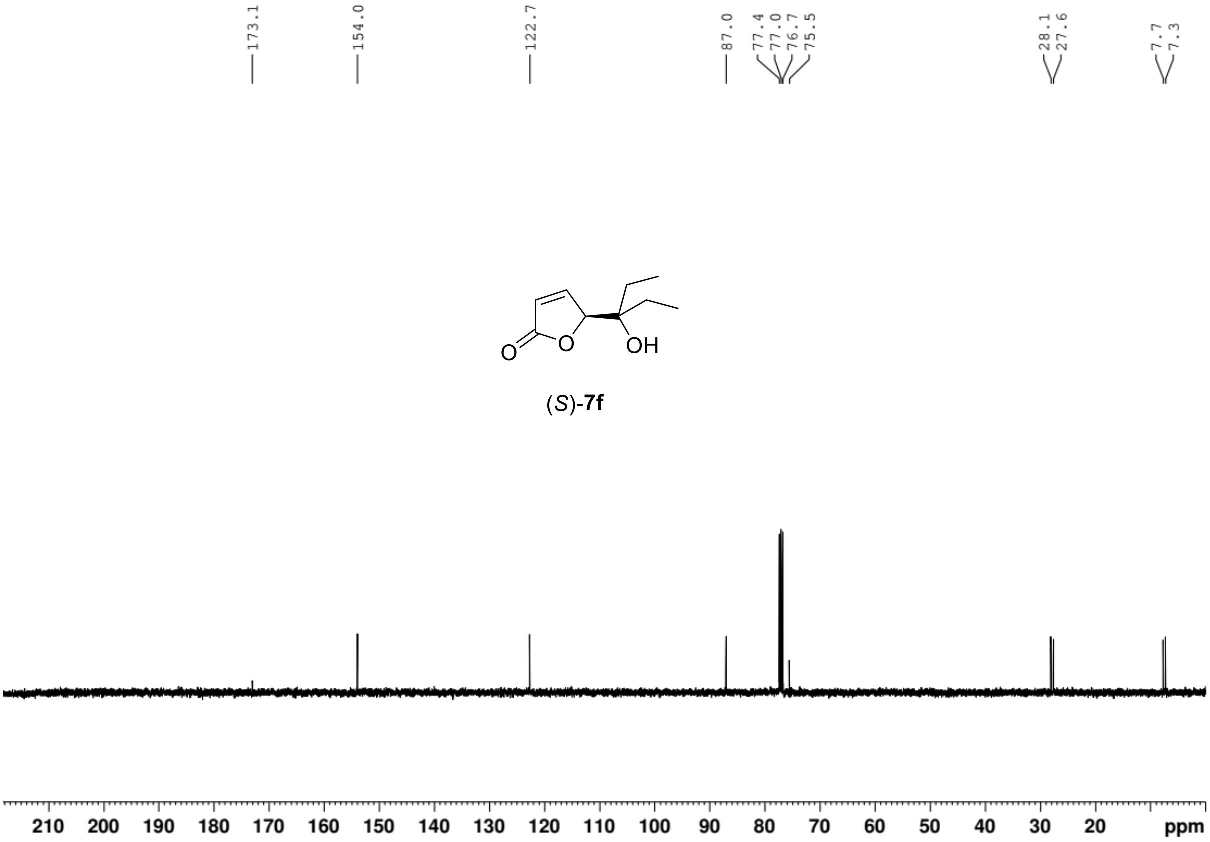
173.1
154.0
122.7
87.0
77.4
77.0
76.7
75.5
28.1
27.6
7.7
7.3
O
O
OH
(S)-7f
210 200 190 180 170 160 150 140 130 120 110 100 90 80 70 60 50 40 30 20 ppm

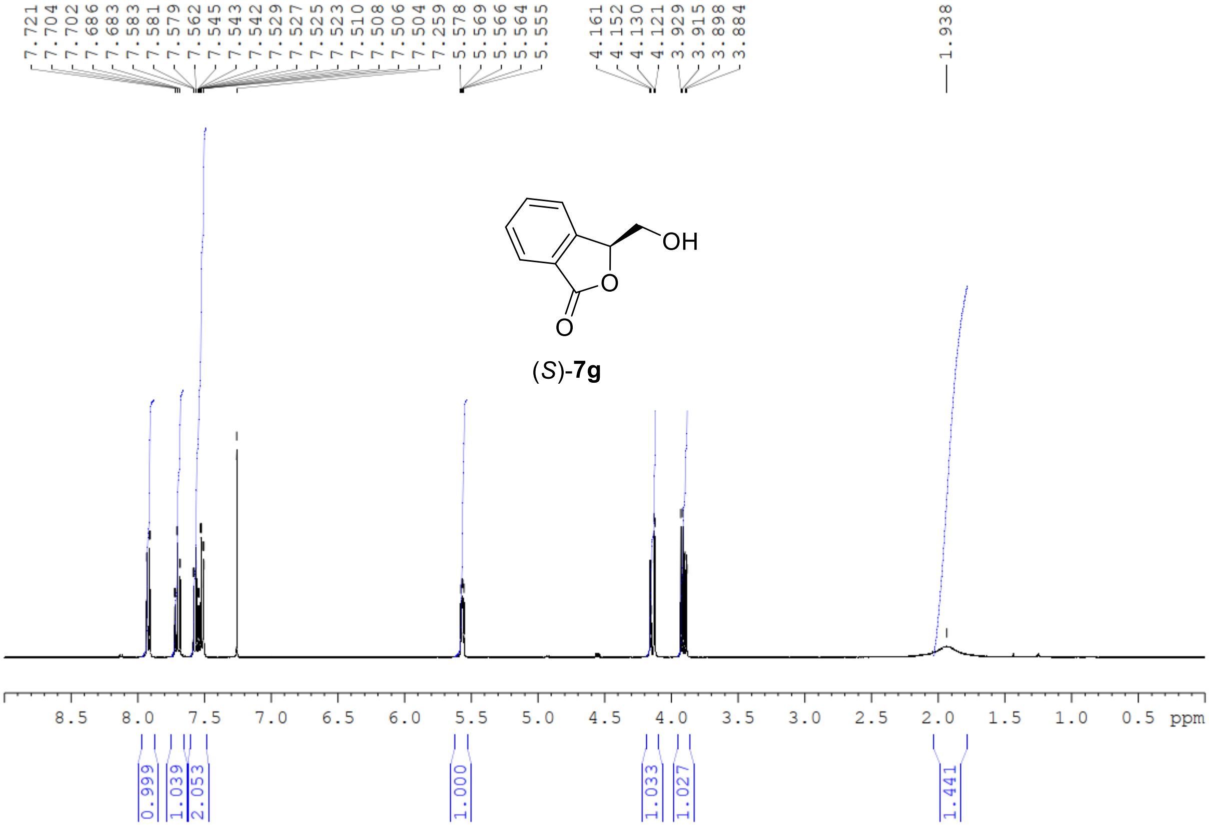
7.721 7.704 7.702 7.686 7.683 7.583 7.581 7.579 7.562 7.545 7.543 7.542 7.529 7.527 7.525 7.523 7.510 7.508 7.506 7.504 7.259 5.578 5.569 5.566 5.564 5.555
4.161 4.152 4.130 4.121 3.929 3.915 3.898 3.884
1.938
OH
O
O
(S)-7g
8.5 8.0 7.5 7.0 6.5 6.0 5.5 5.0 4.5 4.0 3.5 3.0 2.5 2.0 1.5 1.0 0.5 ppm
0.999 1.039 2.053 1.000 1.033 1.027 1.441

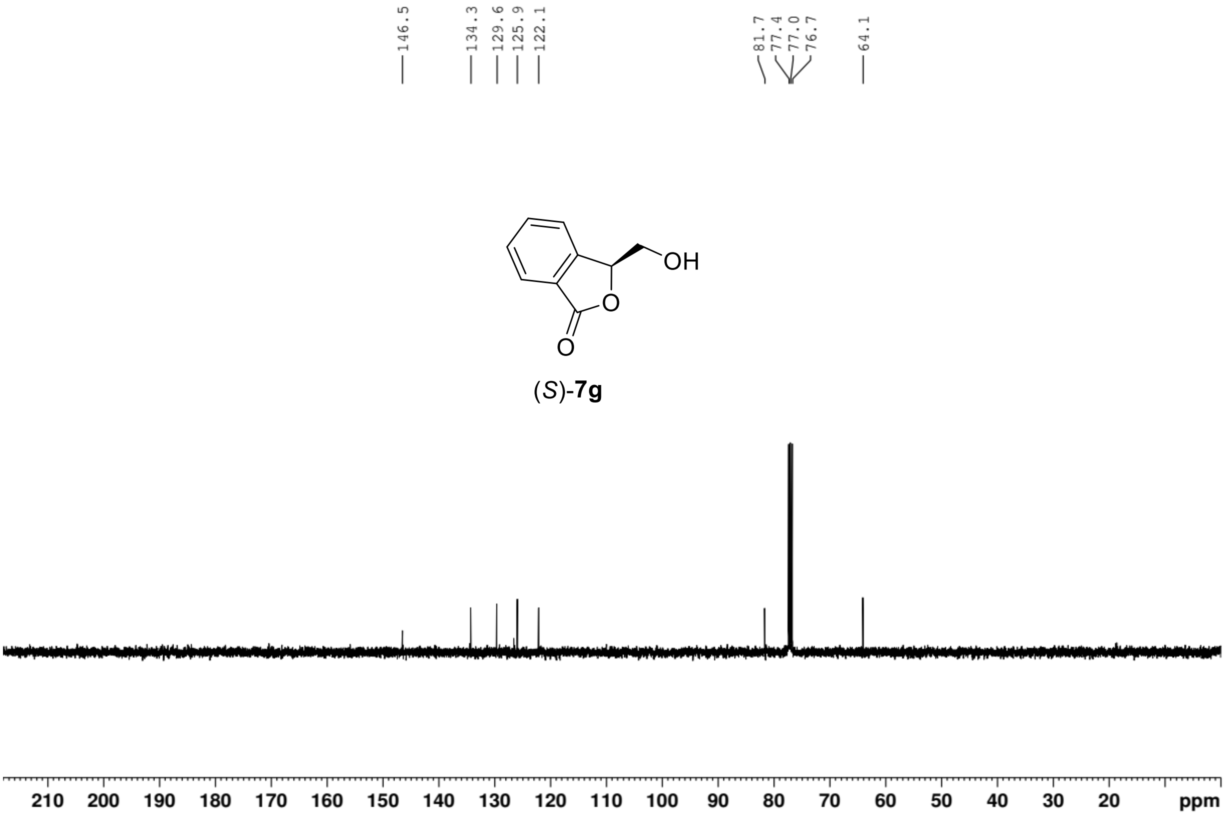
146.5 134.3 129.6 125.9 122.1 81.7 77.4 77.0 76.7 64.1
OH
O
O
(S)-7g
210 200 190 180 170 160 150 140 130 120 110 100 90 80 70 60 50 40 30 20 ppm

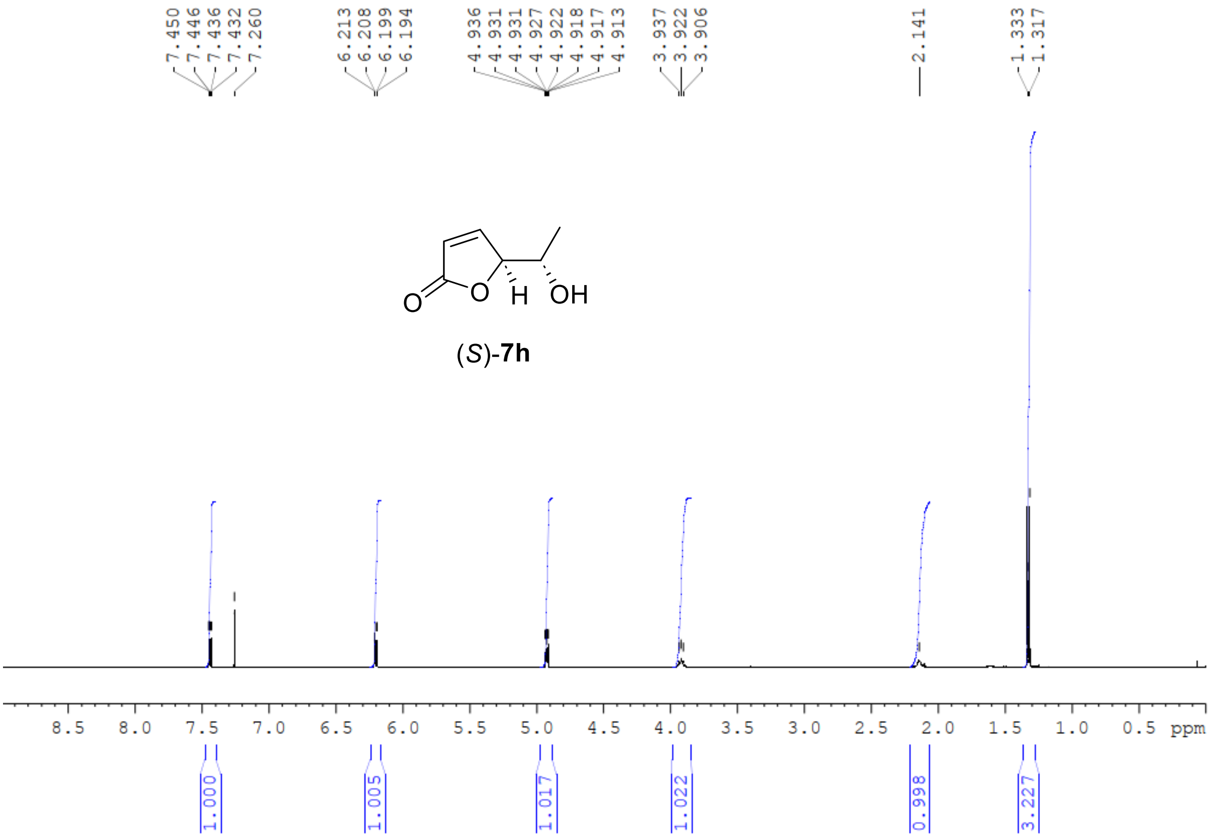
7.450
7.446
7.436
7.432
7.260
6.213
6.208
6.199
6.194
4.936
4.931
4.931
4.927
4.922
4.918
4.917
4.913
3.937
3.922
3.906
2.141
1.333
1.317
O
O
H
OH
(S)-7h
8.5 8.0 7.5 7.0 6.5 6.0 5.5 5.0 4.5 4.0 3.5 3.0 2.5 2.0 1.5 1.0 0.5 ppm
1.000
1.005
1.017
1.022
0.998
3.227

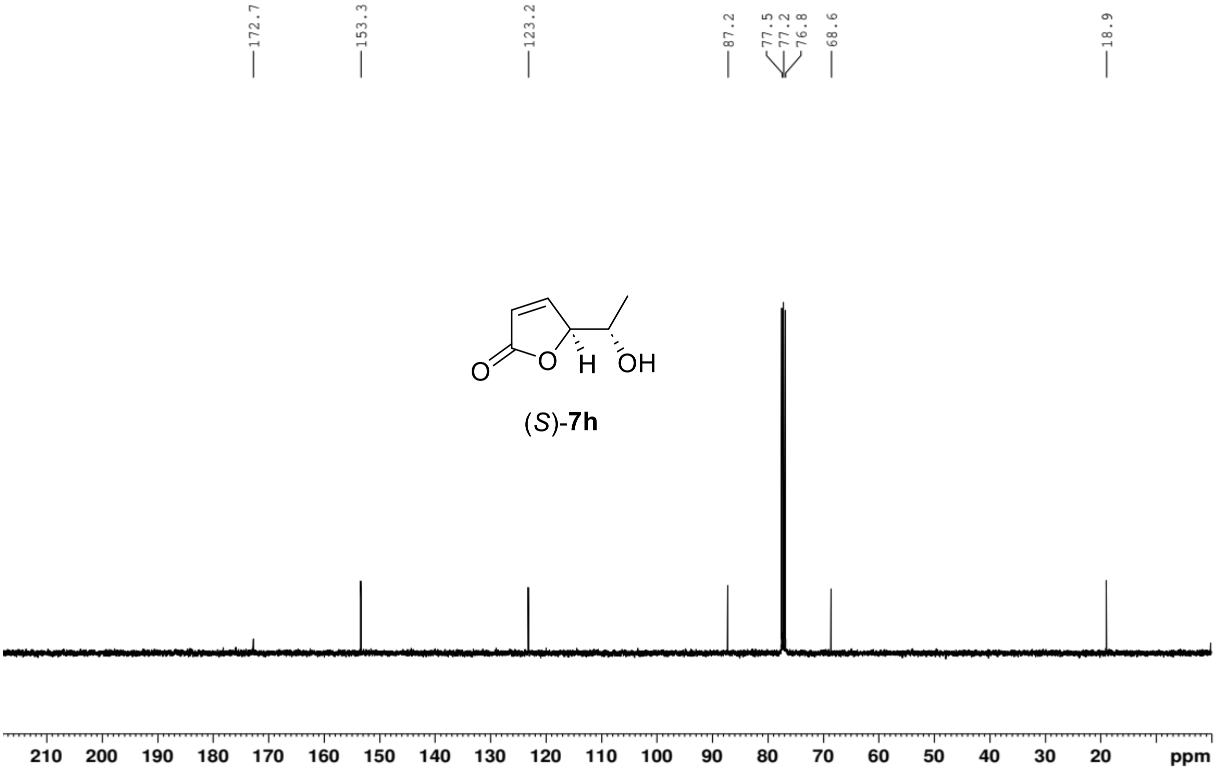
172.7
153.3
123.2
87.2
77.5
77.2
76.8
68.6
18.9
O
O
H
OH
(S)-7h
210 200 190 180 170 160 150 140 130 120 110 100 90 80 70 60 50 40 30 20 ppm

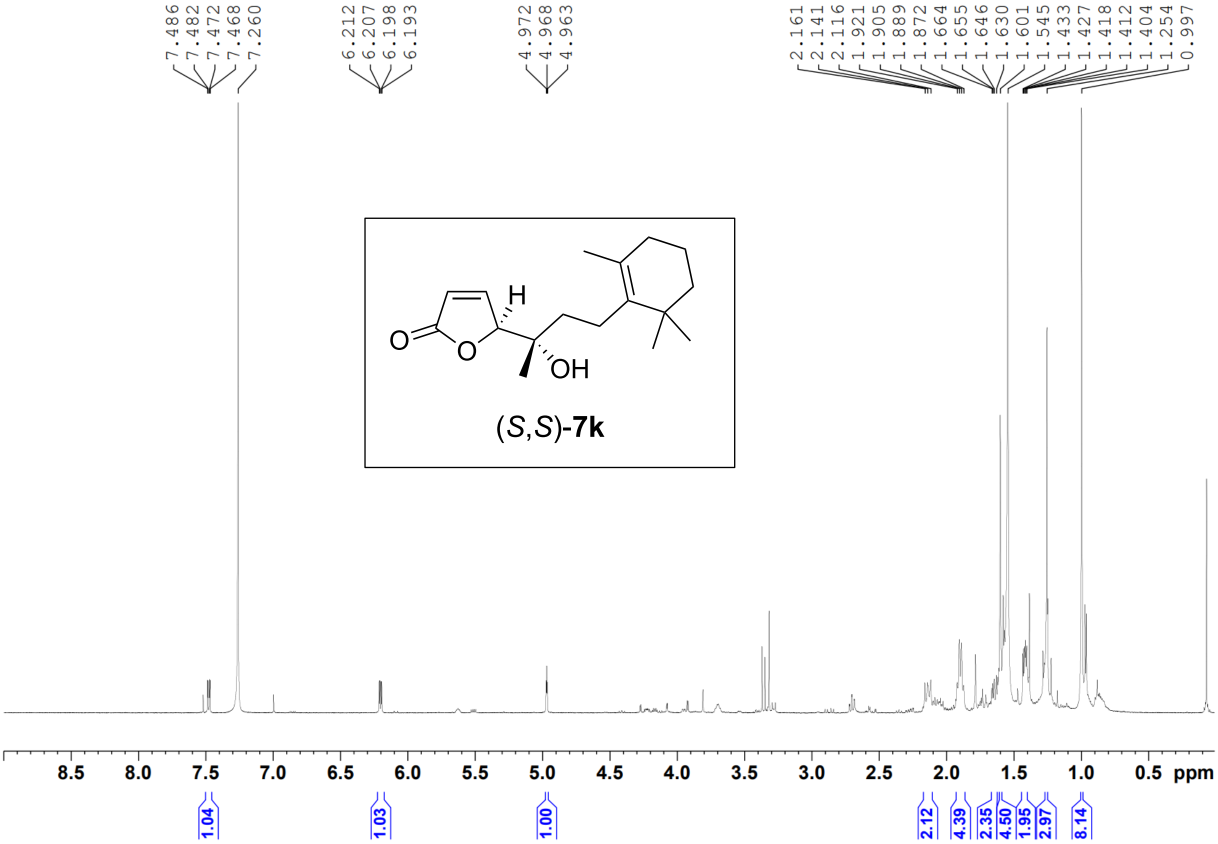
7.486
7.482
7.472
7.468
7.260
6.212
6.207
6.198
6.193
4.972
4.968
4.963
2.161
2.141
2.116
1.921
1.905
1.889
1.872
1.664
1.655
1.646
1.630
1.601
1.545
1.433
1.427
1.418
1.412
1.404
1.254
0.997
H
O
O
OH
(S,S)-7k
ppm
1.04
1.03
1.00
2.12
4.39
2.35
4.50
1.95
2.97
8.14

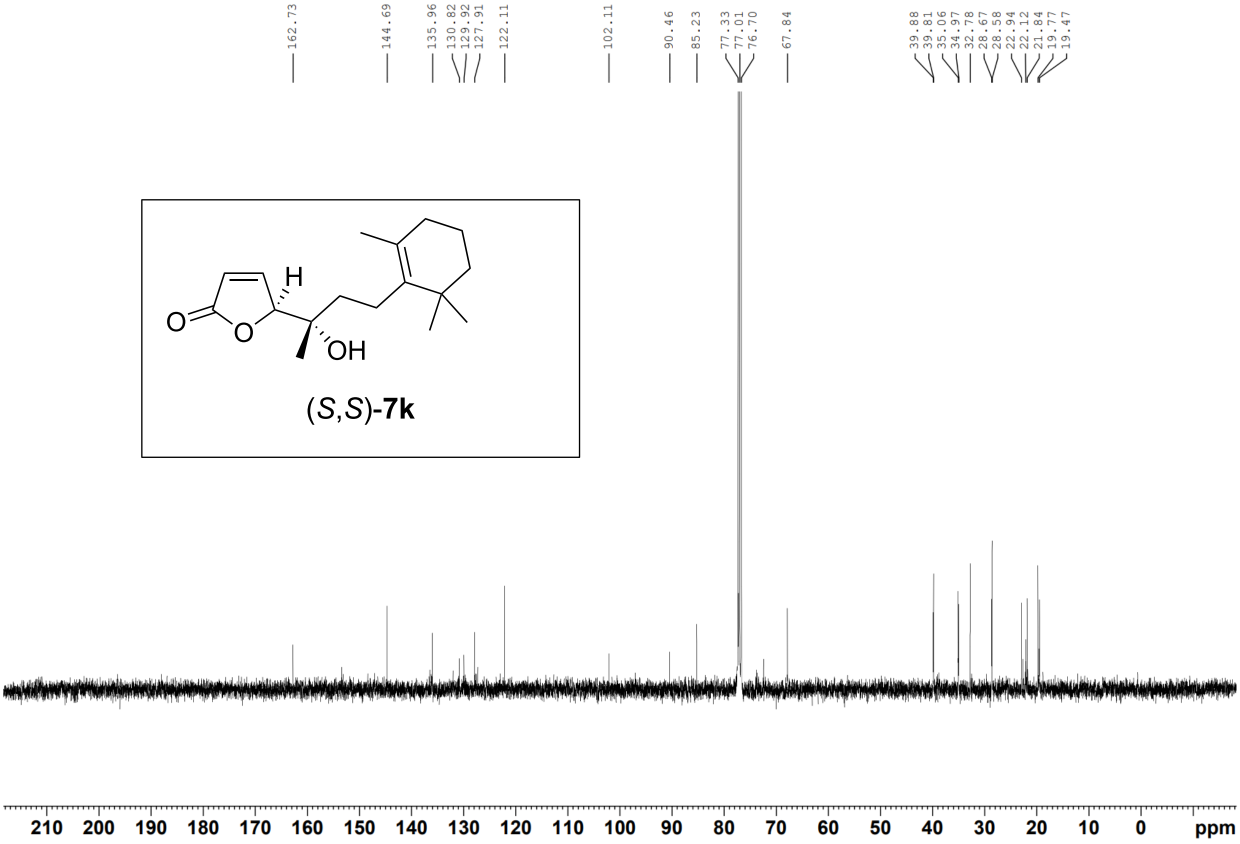
162.73
144.69
135.96
130.82
129.92
127.91
122.11
102.11
90.46
85.23
77.33
77.01
76.70
67.84
39.88
39.81
35.06
34.97
32.78
28.67
28.58
22.94
22.12
21.84
19.77
19.47
H
O
O
OH
(S,S)-7k
ppm

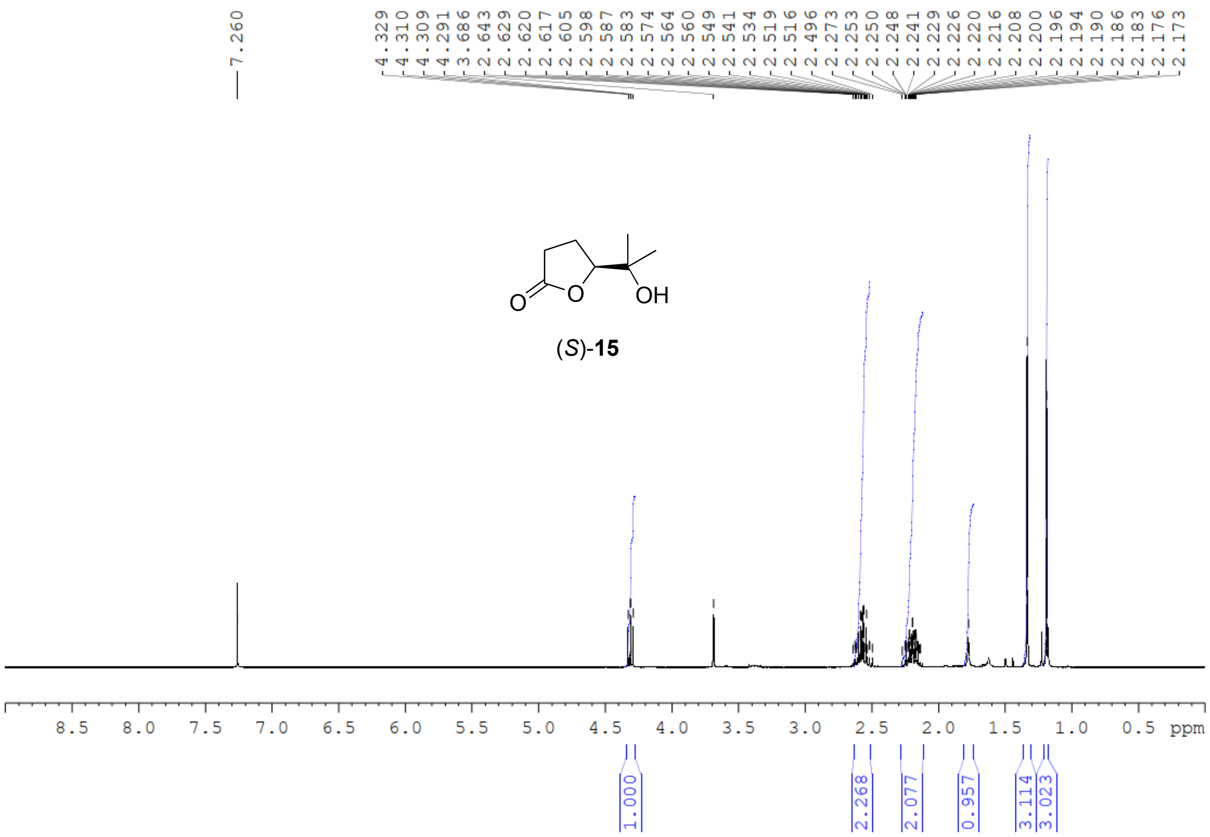
(S)-15
7.260
4.329
4.310
4.309
4.291
3.686
1.000
2.268
2.077
0.957
3.114
3.023
ppm

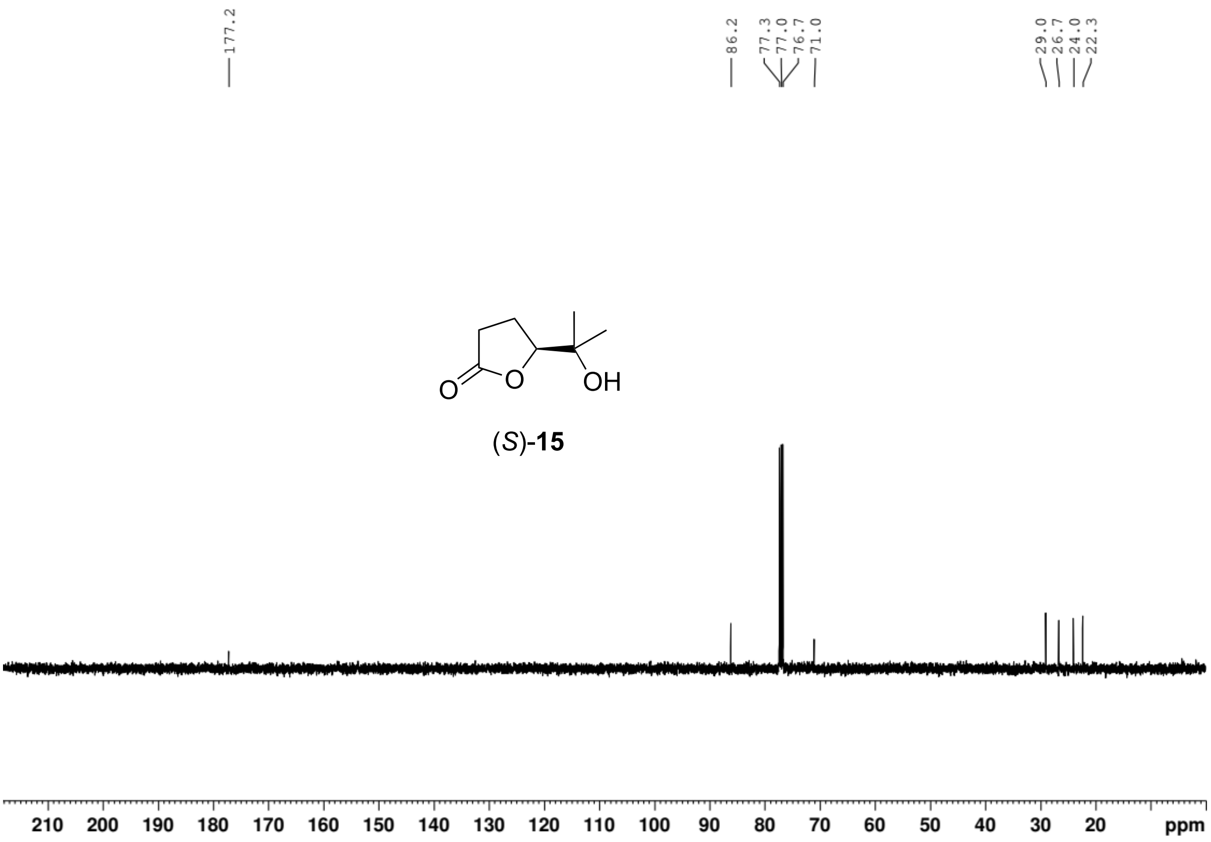
(S)-15
177.2
86.2
77.3
77.0
76.7
71.0
29.0
26.7
24.0
22.3
ppm

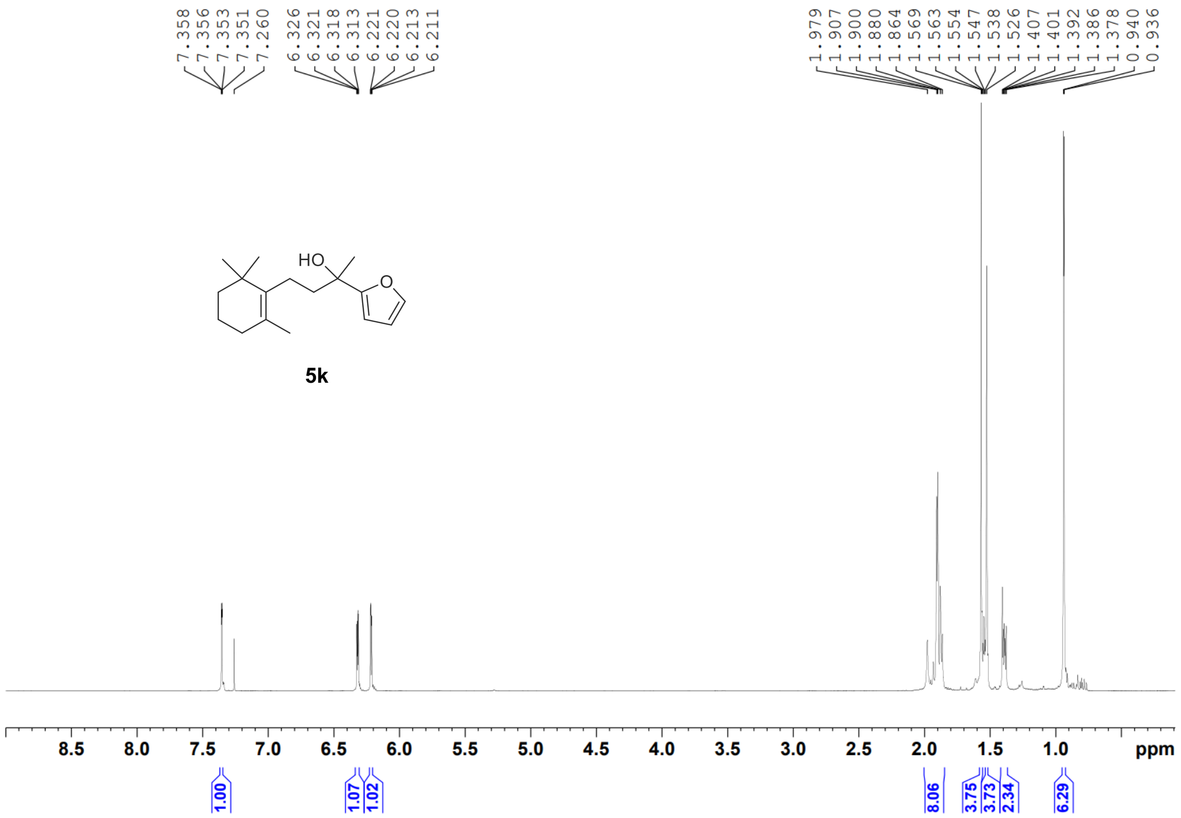
7.358
7.356
7.353
7.351
7.260
6.326
6.321
6.318
6.313
6.221
6.220
6.213
6.211
1.979
1.907
1.900
1.880
1.864
1.569
1.563
1.554
1.547
1.538
1.526
1.407
1.401
1.392
1.386
1.378
0.940
0.936
HO
O
5k
8.5 8.0 7.5 7.0 6.5 6.0 5.5 5.0 4.5 4.0 3.5 3.0 2.5 2.0 1.5 1.0 ppm
1.00
1.07
1.02
8.06
3.75
3.73
2.34
6.29

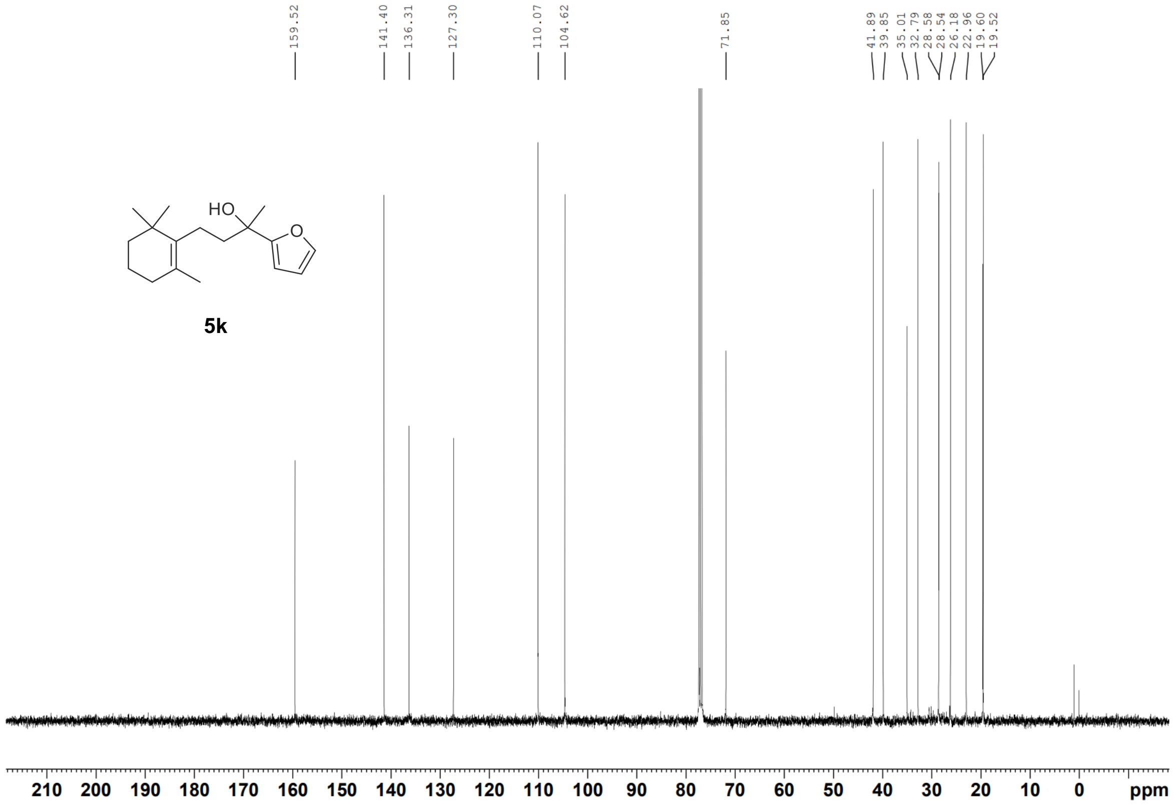
159.52
141.40
136.31
127.30
110.07
104.62
71.85
41.89
39.85
35.01
32.79
28.58
28.54
26.18
22.96
19.60
19.52
HO
O
5k
210 200 190 180 170 160 150 140 130 120 110 100 90 80 70 60 50 40 30 20 10 0 ppm

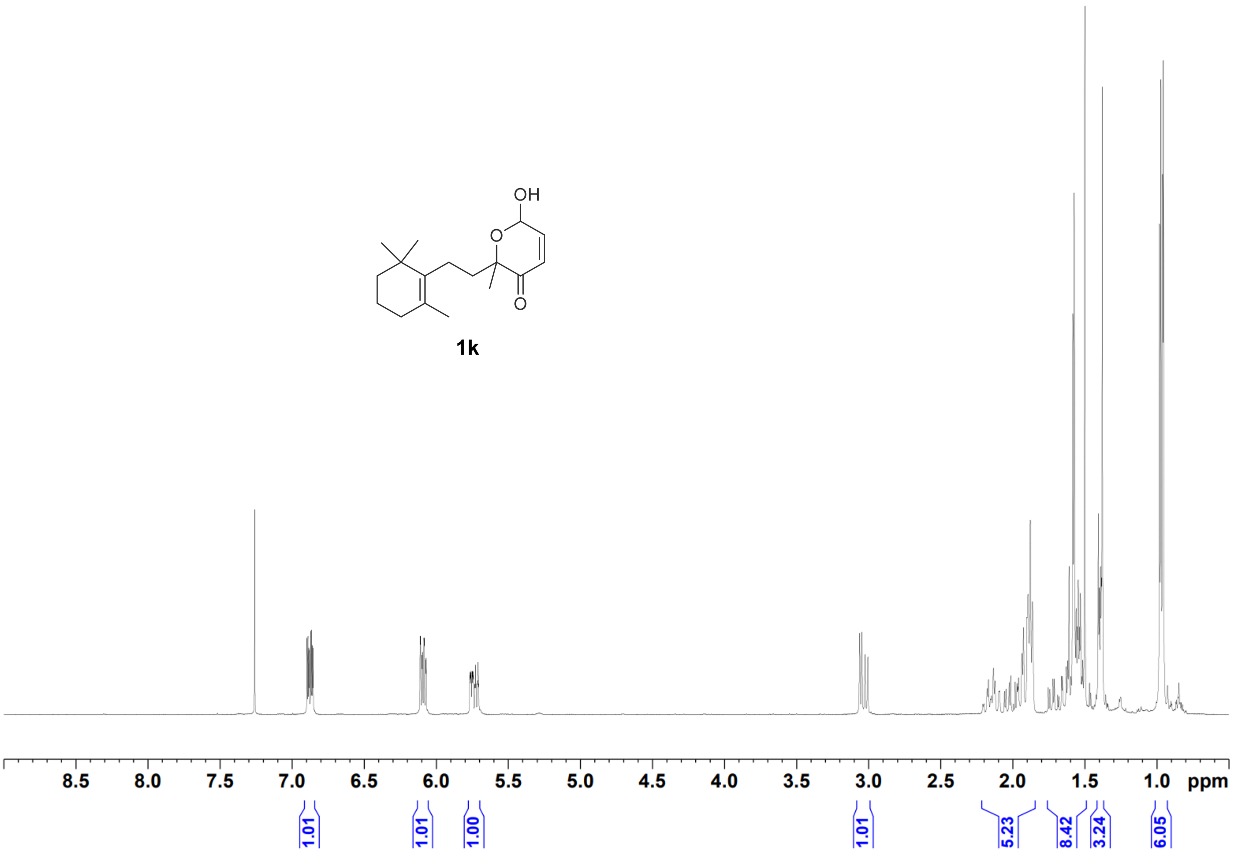
OH
O
O
1k
8.5
8.0
7.5
7.0
6.5
6.0
5.5
5.0
4.5
4.0
3.5
3.0
2.5
2.0
1.5
1.0
ppm
1.01
1.01
1.00
1.01
5.23
8.42
3.24
6.05

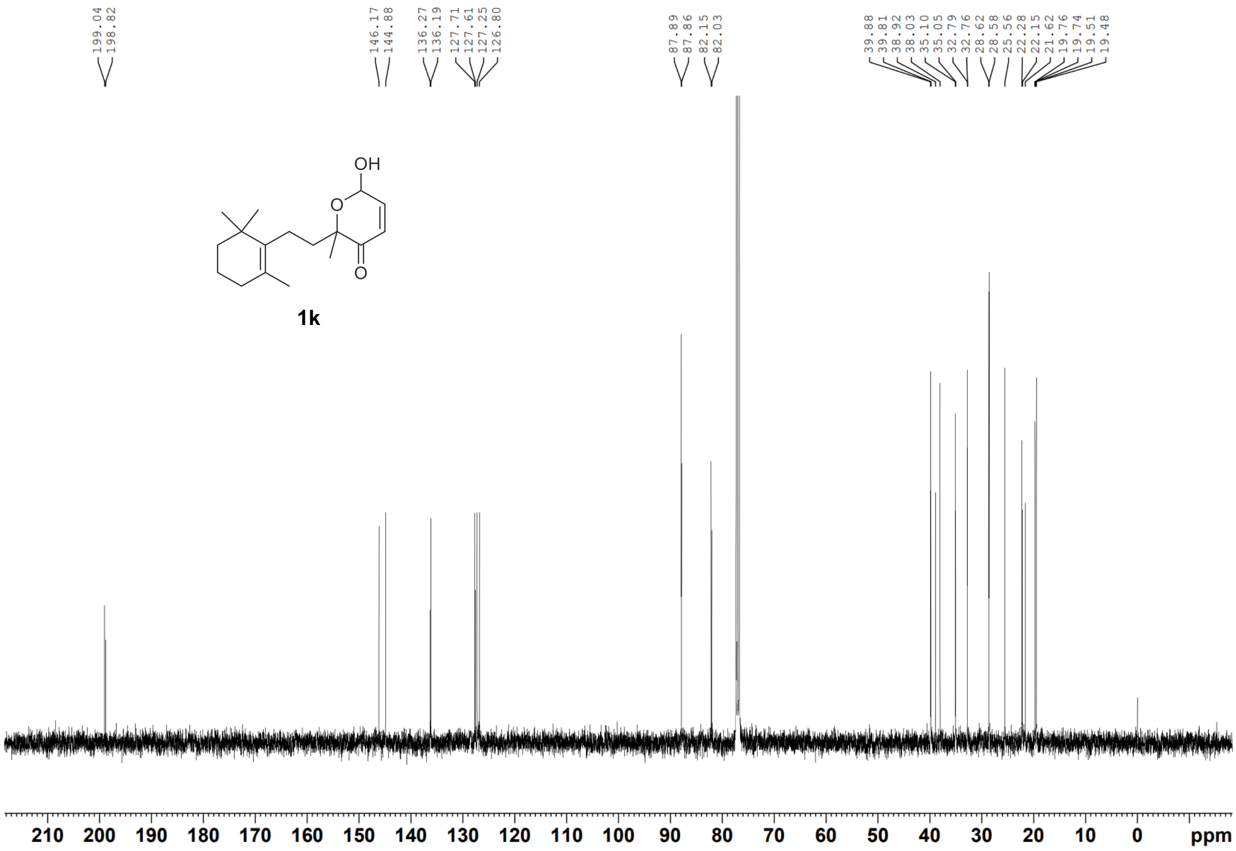
199.04
198.82
146.17
144.88
136.27
136.19
127.71
127.61
127.25
126.80
87.89
87.86
82.15
82.03
39.88
39.81
38.92
38.03
35.10
35.05
32.79
32.76
28.62
28.58
25.56
22.28
22.15
21.62
19.76
19.74
19.51
19.48
OH
O
O
1k
210
200
190
180
170
160
150
140
130
120
110
100
90
80
70
60
50
40
30
20
10
0
ppm

## HPLC and GC traces

```
Data File D:\LCData\Liu\dimethyl 5-lactone_ref_AD_IPA_10%_0.5-2017-10-13-09-02-53.D
Sample Name: dimethyl 5-lactone_ref_AD_IPA_10%_0.5

    =====================================================================
    Acq. Operator   : SYSTEM
    Sample Operator : SYSTEM
    Acq. Instrument : HPLC-1 NP                       Location :   91
    Injection Date  : 13.10.2017 9:04:25
                                                   Inj Volume : 4.000 µl
    Different Inj Volume from Sample Entry!  Actual Inj Volume : 3.000 µl
    Acq. Method     : C:\Chem32\2\Methods\DEF_LC1.M
    Last changed    : 13.10.2017 8:40:12 by SYSTEM
                      (modified after loading)
    Analysis Method : D:\LCDATA\TIURI\Garner_1.M
    Last changed    : 25.9.2017 9:01:33 by SYSTEM
```

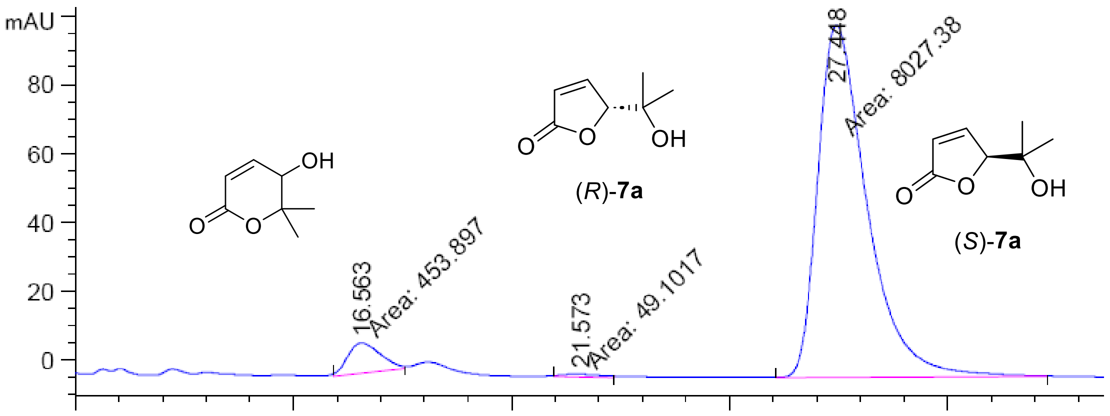


```
Data File D:\LCData\Liu\ADH200+30_Tris_dimethyl_AD_IPA_10%_0.5-2017-10-12-09-19-26.D
Sample Name: ADH200+30_Tris_dimethyl_AD_IPA_10%_0.5

    =====================================================================
    Acq. Operator   : SYSTEM
    Sample Operator : SYSTEM
    Acq. Instrument : HPLC-1 NP                       Location :   51
    Injection Date  : 12.10.2017 9:20:52
                                                   Inj Volume : 4.000 µl
    Different Inj Volume from Sample Entry!  Actual Inj Volume : 5.000 µl
    Acq. Method     : C:\Chem32\2\Methods\DEF_LC1.M
    Last changed    : 12.10.2017 9:03:07 by SYSTEM
                      (modified after loading)
    Analysis Method : D:\LCDATA\TIURI\Garner_1.M
    Last changed    : 25.9.2017 9:01:33 by SYSTEM
    Additional Info : Peak(s) manually integrated
```

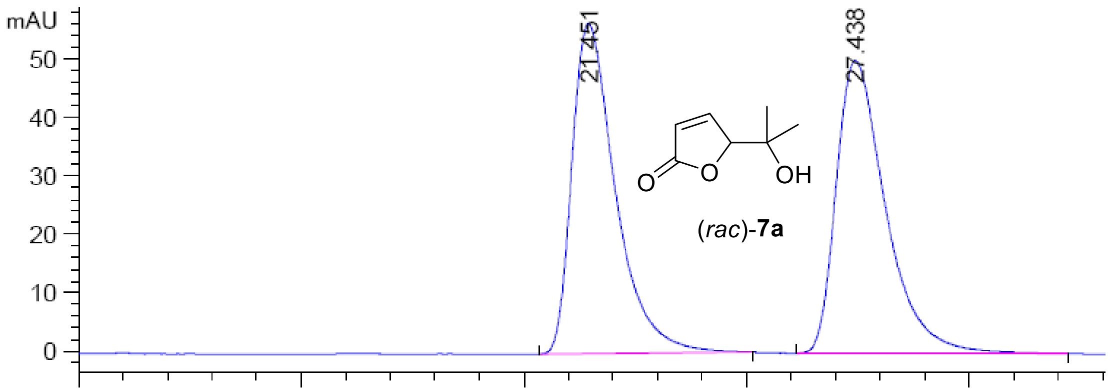

Data File D:\LCData\Liu\p2-G03+adh30_1S_Hexanyl_AD_IPA_10%_0.5-2017-10-09-14-02-38.D
Sample Name: p2-G03+adh30_1S_Hexanyl_AD_IPA_10%_0.5

```
=====================================================================
Acq. Operator   : SYSTEM
Sample Operator : SYSTEM
Acq. Instrument : HPLC-1 NP                       Location :   63
Injection Date  : 9.10.2017 14:04:05
                                                Inj Volume : 5.000 µl
Acq. Method     : C:\Chem32\2\Methods\DEF_LC1.M
Last changed    : 9.10.2017 9:36:09 by SYSTEM
                  (modified after loading)
Analysis Method : D:\LCDATA\TIURI\Garner_1.M
Last changed    : 25.9.2017 9:01:33 by SYSTEM
Additional Info : Peak(s) manually integrated
```

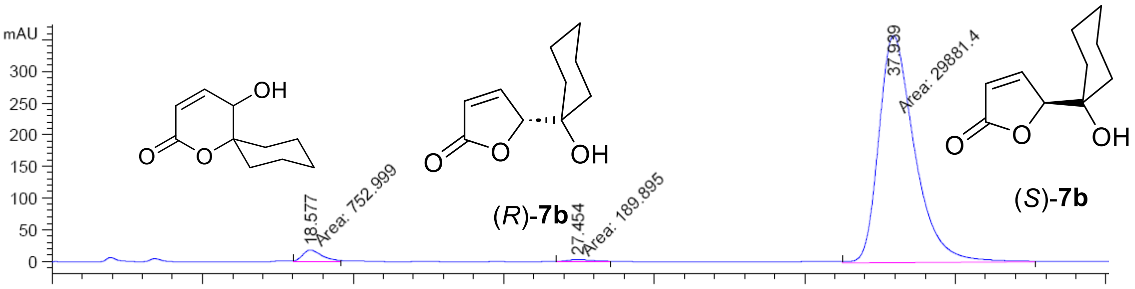


Data File D:\LCData\Liu\Hexanyl 5_lactone_ref_AD_IPA_10%_0.5-2017-10-09-14-58-52.D
Sample Name: Hexanyl 5_lactone_ref_AD_IPA_10%_0.5

```
=====================================================================
Acq. Operator   : SYSTEM
Sample Operator : SYSTEM
Acq. Instrument : HPLC-1 NP                       Location :   95
Injection Date  : 9.10.2017 15:00:19
                                                Inj Volume : 5.000 µl
Different Inj Volume from Sample Entry!  Actual Inj Volume : 4.000 µl
Acq. Method     : C:\Chem32\2\Methods\DEF_LC1.M
Last changed    : 9.10.2017 9:36:09 by SYSTEM
                  (modified after loading)
Analysis Method : D:\LCDATA\TIURI\Garner_1.M
Last changed    : 25.9.2017 9:01:33 by SYSTEM
Additional Info : Peak(s) manually integrated
```

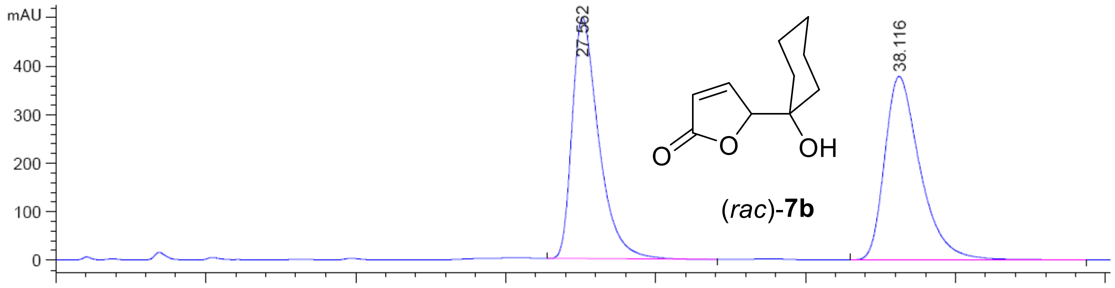

Data File D:\LCData\Liu\p2-G03+adh30_1S_pentanyl_AD_IPA_10%_0.5-2017-10-09-10-13-52.D
Sample Name: p2-G03+adh30_1S_pentanyl_AD_IPA_10%_0.5

=====================================================================
Acq. Operator : SYSTEM
Sample Operator : SYSTEM
Acq. Instrument : HPLC-1 NP Location : 62
Injection Date : 9.10.2017 10:15:21
Inj Volume : 5.000 µl
Acq. Method : C:\Chem32\2\Methods\DEF_LC1.M
Last changed : 9.10.2017 9:36:09 by SYSTEM
(modified after loading)
Analysis Method : D:\LCDATA\TIURI\Garner_1.M
Last changed : 25.9.2017 9:01:33 by SYSTEM
Additional Info : Peak(s) manually integrated

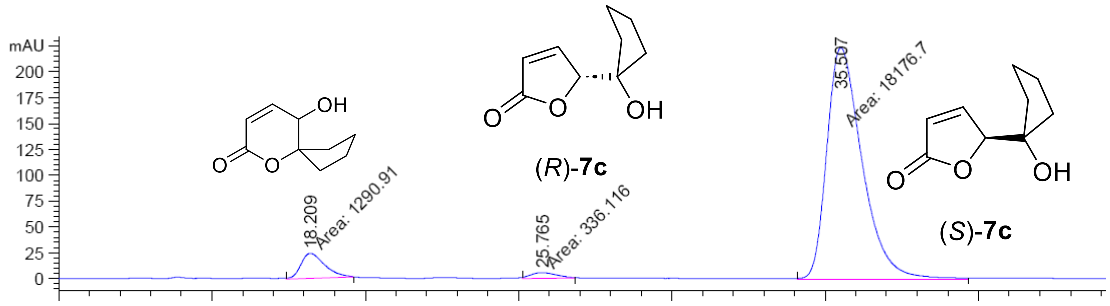


Data File D:\LCData\Liu\Pentanyl 5_lactone_ref_AD_IPA_10%_0.5-2017-10-10-09-33-04.D
Sample Name: Pentanyl 5_lactone_ref_AD_IPA_10%_0.5

=====================================================================
Acq. Operator : SYSTEM
Sample Operator : SYSTEM
Acq. Instrument : HPLC-1 NP Location : 94
Injection Date : 10.10.2017 9:34:32
Inj Volume : 5.000 µl
Different Inj Volume from Sample Entry! Actual Inj Volume : 4.000 µl
Acq. Method : C:\Chem32\2\Methods\DEF_LC1.M
Last changed : 10.10.2017 9:24:20 by SYSTEM
Analysis Method : D:\LCDATA\TIURI\Garner_1.M
Last changed : 25.9.2017 9:01:33 by SYSTEM
Additional Info : Peak(s) manually integrated

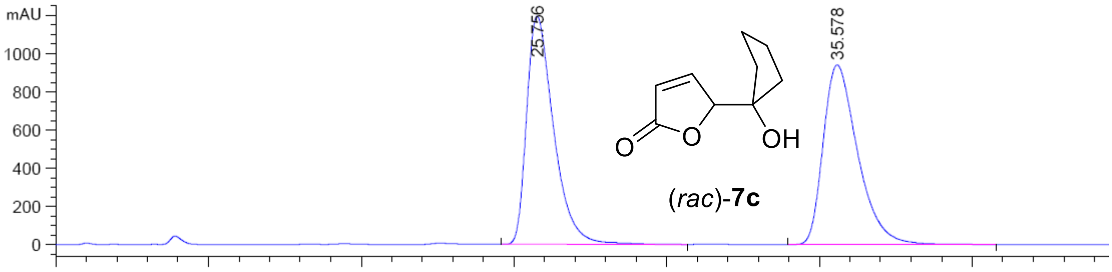

Data File D:\LCData\Liu\p2-g03+adh30_butanyl_2%i_AD_IPA_10%_0.5-2017-10-17-09-50-04.D
Sample Name: p2-g03+adh30_butanyl_2%i_AD_IPA_10%_0.5

```
=====================================================================
Acq. Operator   : SYSTEM
Sample Operator : SYSTEM
Acq. Instrument : HPLC-1 NP                        Location :    52
Injection Date  : 17.10.2017 9:51:35
                                                 Inj Volume : 4.000 µl
Different Inj Volume from Sample Entry!  Actual Inj Volume : 5.000 µl
Acq. Method     : C:\Chem32\2\Methods\DEF_LC1.M
Last changed    : 13.10.2017 8:40:12 by SYSTEM
                  (modified after loading)
Analysis Method : D:\LCDATA\TIURI\Garner_1.M
Last changed    : 17.10.2017 14:09:41 by SYSTEM
                  (modified after loading)
Additional Info : Peak(s) manually integrated
```

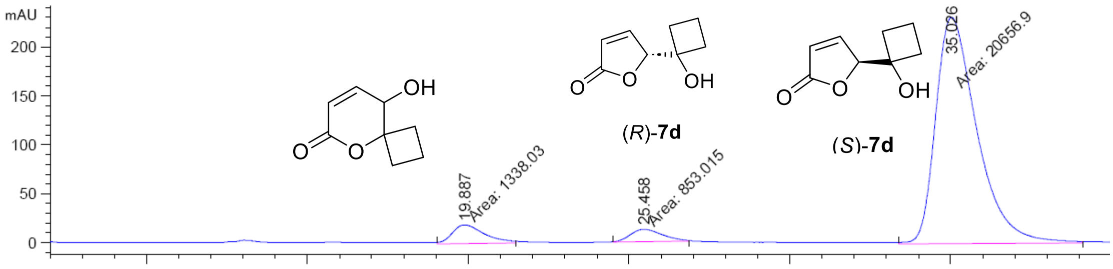


Data File D:\LCData\Liu\butanyl 5-lactone_ref_AD_IPA_10%_0.5-2017-10-17-14-45-35.D
Sample Name: butanyl 5-lactone_ref_AD_IPA_10%_0.5

```
=====================================================================
Acq. Operator   : SYSTEM
Sample Operator : SYSTEM
Acq. Instrument : HPLC-1 NP                        Location :    93
Injection Date  : 17.10.2017 14:47:01
                                                 Inj Volume : 4.000 µl
Different Inj Volume from Sample Entry!  Actual Inj Volume : 2.000 µl
Acq. Method     : C:\Chem32\2\Methods\DEF_LC1.M
Last changed    : 17.10.2017 15:19:50 by SYSTEM
                  (modified after loading)
Analysis Method : D:\LCDATA\TIURI\Garner_1.M
Last changed    : 17.10.2017 14:09:41 by SYSTEM
                  (modified after loading)
Additional Info : Peak(s) manually integrated
```

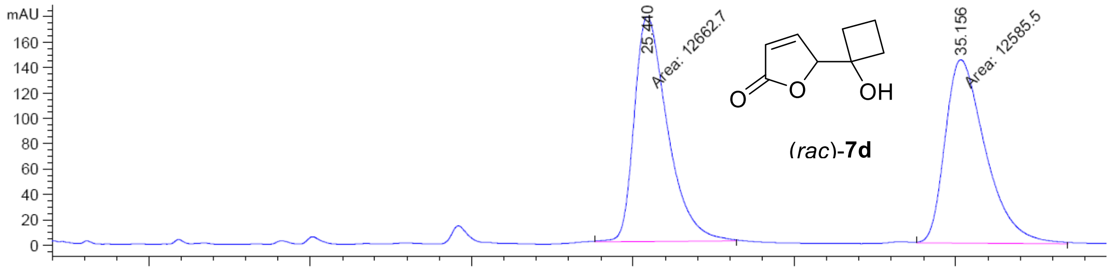

Data File D:\LCData\Liu\P2-G03+ADH30_propanyl_AD-H_IPA_10%_0.5-2017-10-03-09-21-32.D
Sample Name: P2-G03+ADH30_propanyl_AD-H_IPA_10%_0.5

=====================================================================
Acq. Operator : SYSTEM
Sample Operator : SYSTEM
Acq. Instrument : HPLC-1 NP Location : 61
Injection Date : 3.10.2017 9:22:54
Inj Volume : 5.000 µl
Acq. Method : C:\Chem32\2\Methods\DEF_LC1.M
Last changed : 3.10.2017 9:07:12 by SYSTEM
(modified after loading)
Analysis Method : D:\LCDATA\TIURI\Garner_1.M
Last changed : 25.9.2017 9:01:33 by SYSTEM
Additional Info : Peak(s) manually integrated

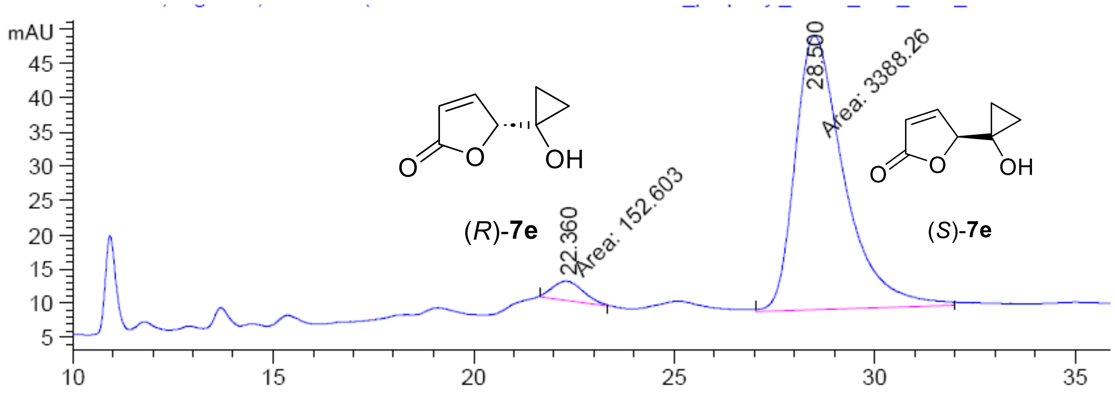


Data File D:\LCData\Liu\cyclopropanyl 5-lactone_AD-H_IPA_10%_0.5-2017-10-05-14-29-11.D
Sample Name: cyclopropanyl 5-lactone_AD-H_IPA_10%_0.5

=====================================================================
Acq. Operator : SYSTEM
Sample Operator : SYSTEM
Acq. Instrument : HPLC-1 NP Location : 71
Injection Date : 5.10.2017 14:30:35
Inj Volume : 3.000 µl
Acq. Method : C:\Chem32\2\Methods\DEF_LC1.M
Last changed : 5.10.2017 13:58:35 by SYSTEM
(modified after loading)
Analysis Method : D:\LCDATA\TIURI\Garner_1.M
Last changed : 25.9.2017 9:01:33 by SYSTEM
Additional Info : Peak(s) manually integrated

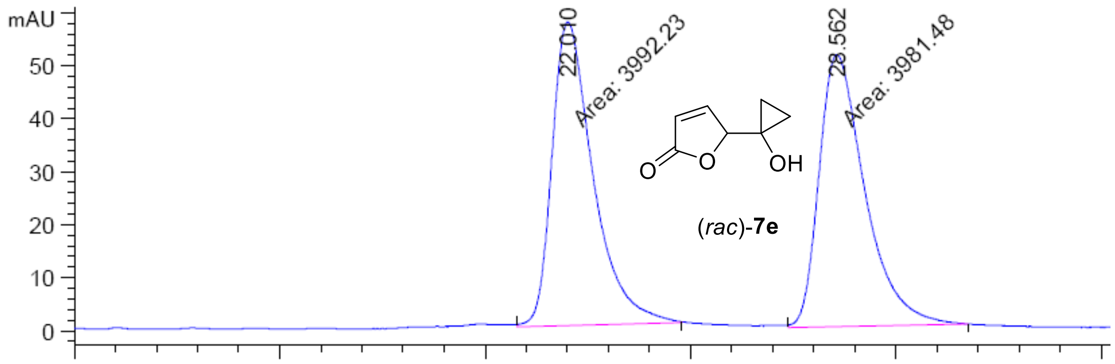

```
Data File D:\LCData\Liu\P2-B02_diethyl lactol_ipa_AD_10%IPA_0.5 2020-06-30 10-29-10.D
Sample Name: P2-B02_diethyl lactol_ipa_AD_10%IPA_0.5

    =====================================================================
    Acq. Operator    : SYSTEM
    Sample Operator  : SYSTEM
    Acq. Instrument  : HPLC-1 NP                         Location :   52
    Injection Date   : 30.6.2020 10:30:42
                                                       Inj Volume : 3.000 µl
    Different Inj Volume from Sample Entry!  Actual Inj Volume : 5.000 µl
    Acq. Method      : C:\Chem32\2\Methods\DEF_LC1.M
    Last changed     : 30.6.2020 8:47:12 by SYSTEM
                       (modified after loading)
    Analysis Method  : C:\Chem32\2\Methods\DEF_LC.M
    Last changed     : 30.6.2020 15:01:39 by SYSTEM
                       (modified after loading)
    Additional Info  : Peak(s) manually integrated
```

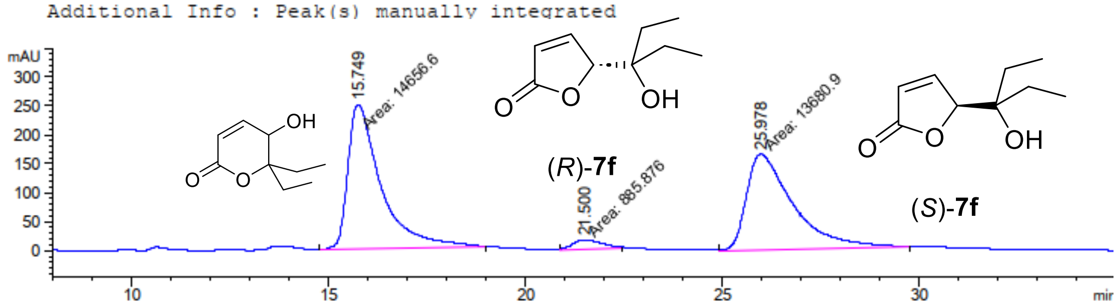


```
Data File D:\LCData\Liu\diethyl 5m lactone_AD_10%IPA_0.5 2020-06-30 14-23-10.D
Sample Name: diethyl 5m lactone_AD_10%IPA_0.5

    =====================================================================
    Acq. Operator    : SYSTEM
    Sample Operator  : SYSTEM
    Acq. Instrument  : HPLC-1 NP                         Location :   61
    Injection Date   : 30.6.2020 14:24:37
                                                       Inj Volume : 3.000 µl
    Acq. Method      : C:\Chem32\2\Methods\DEF_LC1.M
    Last changed     : 30.6.2020 8:47:12 by SYSTEM
                       (modified after loading)
    Analysis Method  : C:\Chem32\2\Methods\DEF_LC.M
    Last changed     : 30.6.2020 15:01:39 by SYSTEM
                       (modified after loading)
    Additional Info  : Peak(s) manually integrated
```

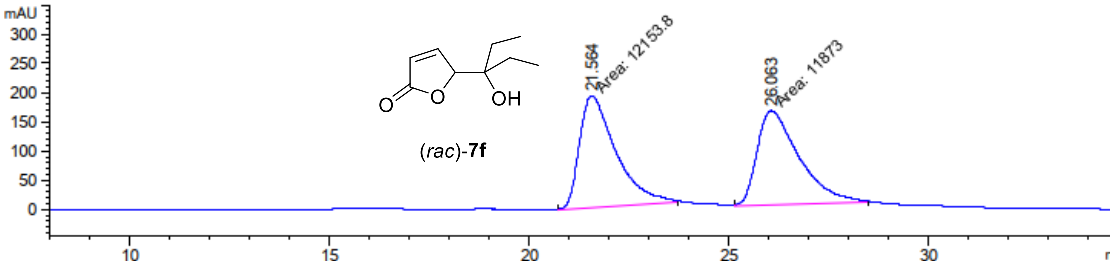

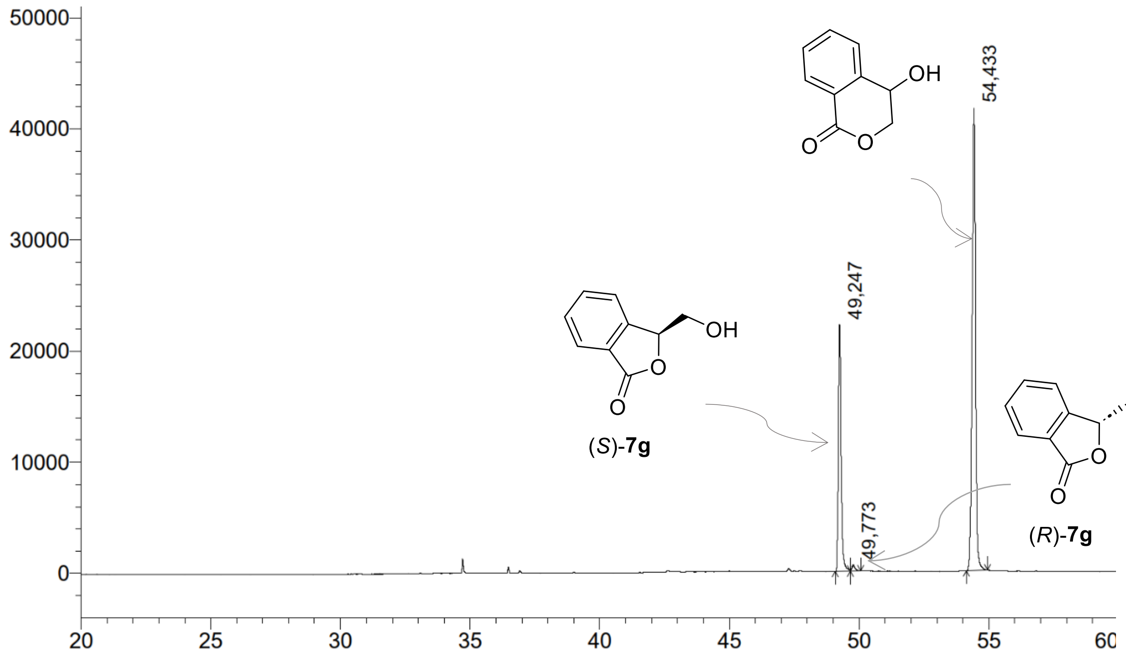


FID1

| Peak# | Ret. Time | Area | Height | Conc. |
|---|---|---|---|---|
| 1 | 49,247 | 160322 | 22195 | 29,391 |
| 2 | 49,773 | 4804 | 548 | 0,881 |
| 3 | 54,433 | 380351 | 41597 | 69,728 |
| Total | | 545476 | 64340 | |

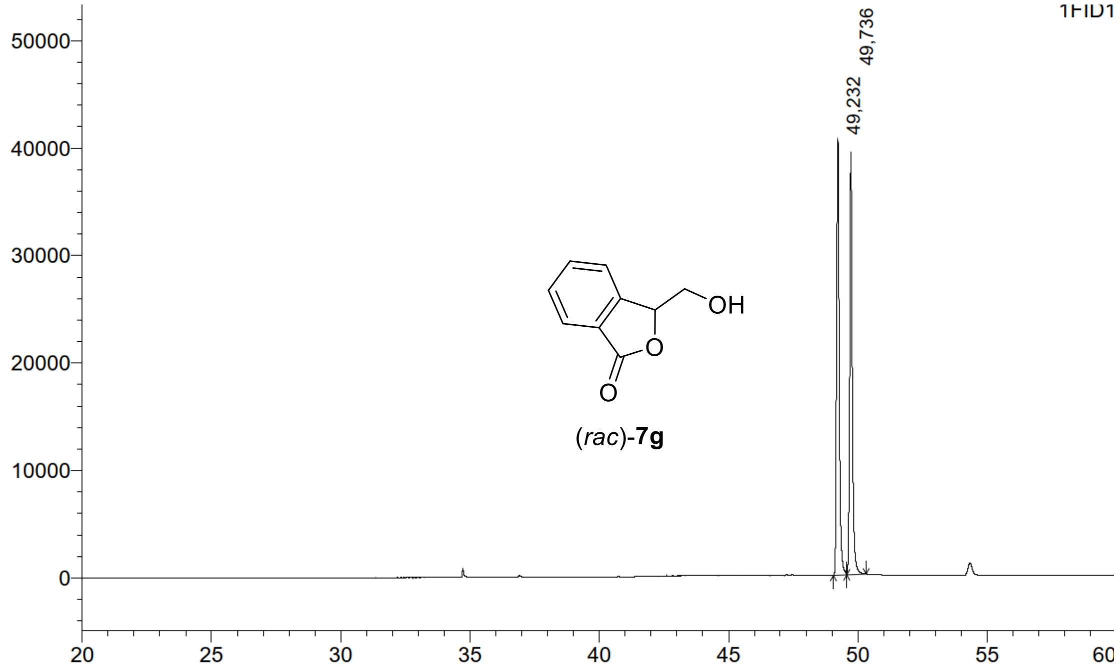


FID1

| Peak# | Ret. Time | Area | Height | Conc. |
|---|---|---|---|---|
| 1 | 49,232 | 280280 | 40476 | 49,247 |
| 2 | 49,736 | 288856 | 39334 | 50,753 |
| Total | | 569136 | 79810 | |

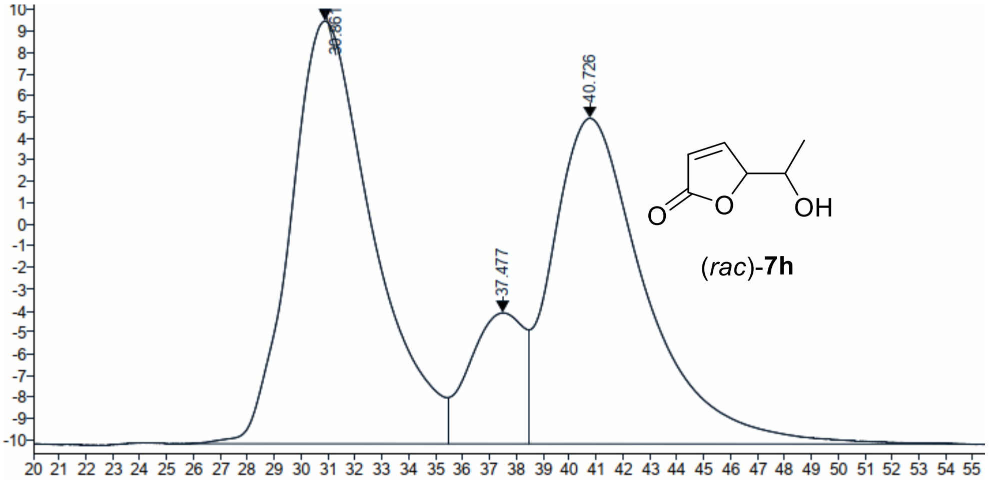


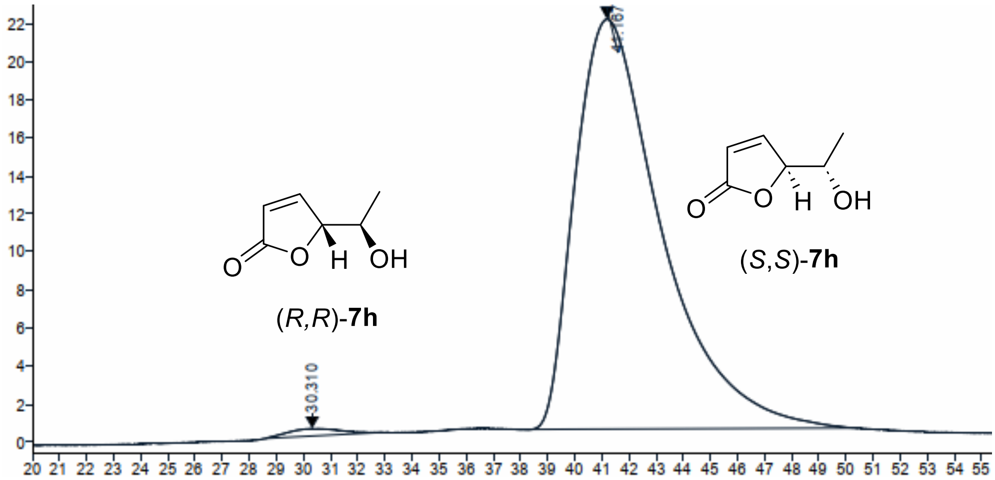


**Signal:** DAD1D,Sig=230,4 Ref=off

| RT [min] | Name | Area | Area% |
|---|---|---|---|
| 30.310 | | 51.5231 | 1.0662 |
| 41.167 | | 4781.0213 | 98.9338 |

HPLC chromatograms

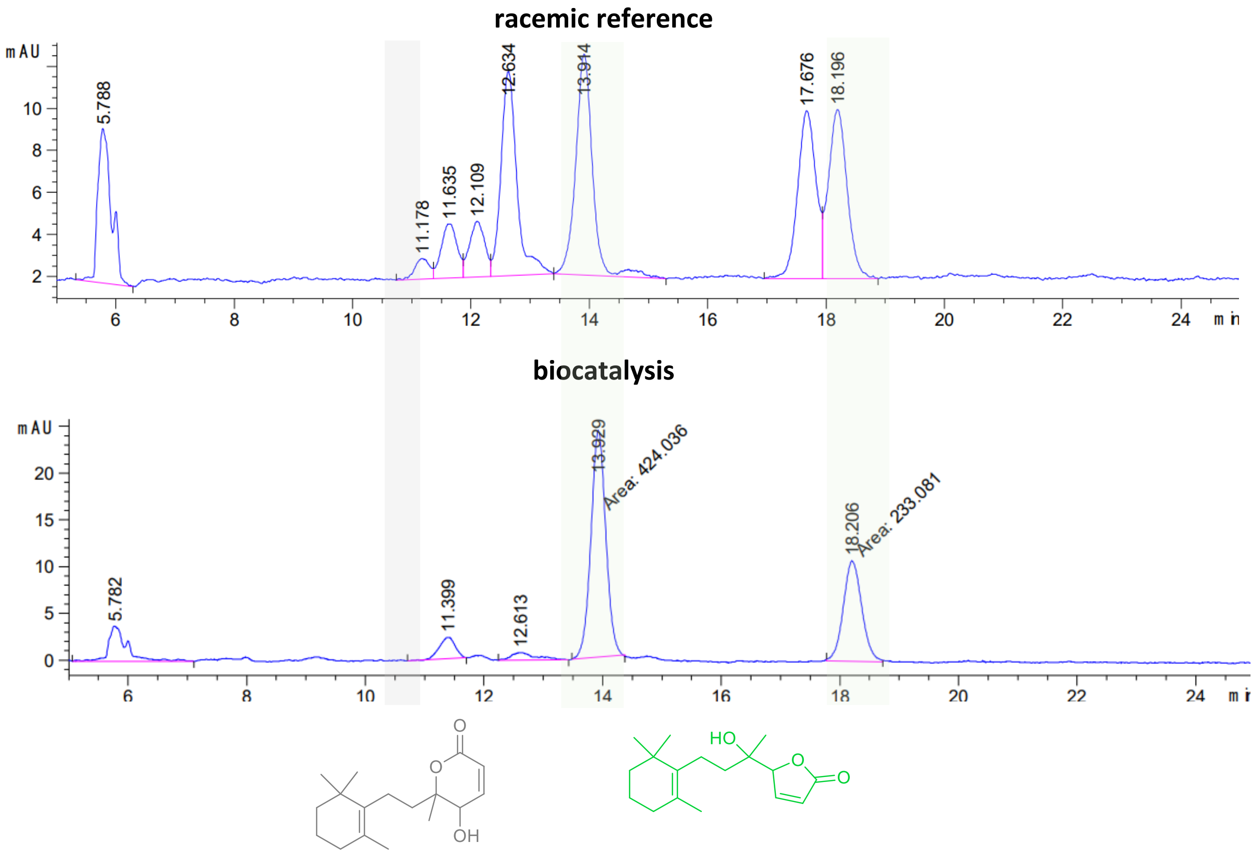

## Supplementary References


1. Liu, Y.-C., Merten, C., Deska, J., *Enantioconvergent biocatalytic redox isomerization. Angew. Chem. Int. Ed.* 2018, **57**, p.12151-12156.
2. Neese, F., *The ORCA program system.* Wiley Interdisciplinary Reviews: Computational Molecular Science, 2012. **2**(1): p. 73-78.
3. Neese, F., *Software update: the ORCA program system, version 4.0.* Wiley Interdisciplinary Reviews: Computational Molecular Science, 2018. **8**(1).
4. Adamo, C. and V. Barone, *Toward reliable density functional methods without adjustable parameters: The PBE0 model.* The Journal of Chemical Physics, 1999. **110**(13): p. 6158-6170.
5. Weigend, F. and R. Ahlrichs, *Balanced basis sets of split valence, triple zeta valence and quadruple zeta valence quality for H to Rn: Design and assessment of accuracy.* Phys Chem Chem Phys, 2005. **7**(18): p. 3297-305.
6. Grimme, S., et al., *A consistent and accurate ab initio parametrization of density functional dispersion correction (DFT-D) for the 94 elements H-Pu.* J Chem Phys, 2010. **132**(15): p. 154104.
7. Grimme, S., S. Ehrlich, and L. Goerigk, *Effect of the damping function in dispersion corrected density functional theory.* J Comput Chem, 2011. **32**(7): p. 1456-65.
8. Neese, F., *An improvement of the resolution of the identity approximation for the formation of the Coulomb matrix.* J Comput Chem, 2003. **24**(14): p. 1740-7.
9. Weigend, F., *Accurate Coulomb-fitting basis sets for H to Rn.* Phys Chem Chem Phys, 2006. **8**(9): p. 1057-65.
10. Neese, F., et al., *Efficient, approximate and parallel Hartree–Fock and hybrid DFT calculations. A 'chain-of-spheres' algorithm for the Hartree–Fock exchange.* Chemical Physics, 2009. **356**(1-3): p. 98-109.
11. Hanwell, M.D., et al., *Avogadro: an advanced semantic chemical editor, visualization, and analysis platform.* J Cheminform, 2012. **4**(1): p. 17.
12. Riplinger, C. and F. Neese, *An efficient and near linear scaling pair natural orbital based local coupled cluster method.* J Chem Phys, 2013. **138**(3): p. 034106.
13. Riplinger, C., et al., *Natural triple excitations in local coupled cluster calculations with pair natural orbitals.* J Chem Phys, 2013. **139**(13): p. 134101.
14. Riplinger, C., et al., *Sparse maps--A systematic infrastructure for reduced-scaling electronic structure methods. II. Linear scaling domain based pair natural orbital coupled cluster theory.* J Chem Phys, 2016. **144**(2): p. 024109.
15. Saitow, M., et al., *A new near-linear scaling, efficient and accurate, open-shell domain-based local pair natural orbital coupled cluster singles and doubles theory.* J Chem Phys, 2017. **146**(16): p. 164105.
16. Guo, Y., et al., *Communication: An improved linear scaling perturbative triples correction for the domain based local pair-natural orbital based singles and doubles coupled cluster method [DLPNO-CCSD(T)].* J Chem Phys, 2018. **148**(1): p. 011101.
17. Hellweg, A., et al., *Optimized accurate auxiliary basis sets for RI-MP2 and RI-CC2 calculations for the atoms Rb to Rn.* Theoretical Chemistry Accounts, 2007. **117**(4): p. 587-597.
18. Marenich, A.V., C.J. Cramer, and D.G. Truhlar, *Universal Solvation Model Based on Solute Electron Density and on a Continuum Model of the Solvent Defined by the Bulk Dielectric Constant and Atomic Surface Tensions.* The Journal of Physical Chemistry B, 2009. **113**(18): p. 6378-6396.
